# A Modular Mathematical Model of the Immune Response for Investigating the Pathogenesis of Infectious Diseases

**DOI:** 10.3390/v17050589

**Published:** 2025-04-22

**Authors:** Maxim I. Miroshnichenko, Fedor A. Kolpakov, Ilya R. Akberdin

**Affiliations:** Department of Computational Biology, Scientific Center for Genetics and Life Sciences, Sirius University of Science and Technology, 354340 Sochi, Russia; kolpakov.fa@talantiuspeh.ru (F.A.K.); akberdin.ir@talantiuspeh.ru (I.R.A.)

**Keywords:** coronavirus, SARS-CoV-2, COVID-19, mathematical model, BioUML, immune response

## Abstract

The COVID-19 pandemic highlighted the importance of mathematical modeling for understanding viral infection dynamics and accelerated its application into immunological research. Collaborative efforts among international research groups yielded a wealth of experimental data, which facilitated model development and validation. This study focuses on developing a modular mathematical model of the immune response, capturing the interactions between innate and adaptive immunity, with an application to SARS-CoV-2 infection. The model was validated using experimental data from middle-aged individuals with moderate COVID-19 progression, including measurements of viral load in the upper and lower airways, serum antibodies, CD4+ and CD8+ T cells, and interleukin-6 levels. Parameter optimization and sensitivity analysis were performed to improve the model accuracy. Additionally, identifiability analysis was conducted to assess whether the data were sufficient for reliable parameter estimation. The verified model simulates the dynamics of moderate, severe, and critical COVID-19 progressions using measured data on lung epithelium damage, viral load, and IL-6 levels as key indicators of disease severity. We also performed a series of validation scenarios to assess whether the model correctly reproduces biologically relevant behaviors under various conditions, such as immunity hyperactivation, co-infection with HIV, and interferon administration as a therapeutic strategy. The model was developed as a component of the Digital Twin project and represents a general immune module that integrates both innate and adaptive immunity. It can be utilized for further COVID-19 research or serve as a foundation for studying other infectious diseases, provided sufficient data are available.

## Author Summary

Despite the progress reached in understanding COVID-19, traditional methods still struggle to analyze and interpret the extensive and sometimes controversial experimental data on SARS-CoV-2 infection. Mathematical and systems biology approaches attempt to address this challenge by developing mathematical models of the immune response. We aimed not only to investigate the disease at a systemic level but also to provide a framework for further research on host–pathogen interactions, both existing and forthcoming. To achieve this, we constructed a model incorporating both innate and adaptive immunity, as well as cellular and humoral components. This together allowed us to conduct a series of in silico experiments, exploring the immune response across various levels and compartments. The results of these investigations offer valuable insights into the complex dynamics of the immune system and can guide future research and therapeutic strategies.

## 1. Introduction

The emergence of severe acute respiratory syndrome coronavirus 2 (SARS-CoV-2) led to one of the largest pandemics in human history, comparable with the Spanish flu and HIV/AIDS in terms of case numbers [1]. SARS-CoV-2 causes coronavirus disease 2019 (COVID-19) and is classified as a positive-sense single-stranded RNA virus [2] that infects human cells via angiotensin-converting enzyme 2 (ACE2). It is a common membrane receptor expressed in most tissues and organs, primarily including the gastrointestinal tract, upper and lower airways, and circulatory system. Additionally, it is present in the brain, kidneys, testis, and other organs [3]. According to the National Institutes of Health, COVID-19 manifestations are categorized into five groups: asymptomatic infection, and mild, moderate, severe, and critical conditions [4]. The distinction between mild and severe forms includes the progression of the virus to the lower respiratory tract and a decline in oxygen saturation level. In severe cases, this leads to more serious symptoms, which, in the most critical cases, may result in respiratory failure and multiple organ dysfunction. The severity of COVID-19 depends on many factors, with age and chronic conditions such as asthma, cancer, or diabetes being among the most significant [5]. It has been shown that the efficiency and quantity of various arms of the immune system diminish with age. These changes include a decrease in the activity of both neutralizing and non-neutralizing antibodies [6], as well as a reduction in the number of circulating T cells, particularly memory and differentiated effector T cells. Additionally, there is a decline in the proportion of T cells capable of producing cytotoxic molecules such as granzymes and perforins, which lead to impaired effector functions of T cells [7]. Similar processes also affect B cell function [8,9]. All of these factors are discussed in detail in Section 3.4.

Mathematical modeling is a common approach for studying various infections and following immune responses. While numerous mathematical models have been developed for various infectious agents, those affecting human health the most, such as influenza virus or HIV, are particularly prevalent. This is evident from the numerous models created in recent decades [10,11,12,13,14,15,16].

There is wide variability in the complexity of existing mathematical models, ranging from simplified ones with only a few ordinary differential equations [15,17] to more complex models composed of dozens or even multiple reactions [12]. As an example, Hacioglu with co-authors [11] investigated the effects of drugs, including interferons, and initial viral load on influenza disease progression. In contrast, Heldt and co-authors [12] delved into the intracellular aspects of influenza, providing a detailed description of replication, transcription, and translation processes.

With the onset of the COVID-19 pandemic, numerous models have emerged, most of which focus on SARS-CoV-2 infecting only the epithelial cells of the human lungs [13,18,19]. Meanwhile, other researchers have explored SARS-CoV-2 infection not just from the perspective of cell interactions but also by considering biochemical processes [20,21,22,23]. They focused on the relationships between intracellular, intercellular, and organism levels, allowing for insights into how the behavior of the entire system changes under varying conditions at each level.

Mathematical models have proven to be effective tools for studying diseases, addressing not only epidemiological aspects but also cell development and their interactions during immune response, intracellular pathogen dynamics, and specific scenarios like airborne transmission [16]. We estimated that over 40 mathematical models related to COVID-19 exist, primarily sourced from the BioModels web repository [24] and through manual searches, highlighting the sustained interest in understanding COVID-19 through mathematical modeling. This may be further facilitated by the availability of experimental data on immune cell and antibody dynamics, as well as biological insights related to SARS-CoV-2, which might be less readily available for other, less well-studied pathogens. Moreover, even after the end of the pandemic, the World Health Organization continues to report registered COVID-19 cases in its weekly epidemiological updates for the last few years [25], thereby demonstrating the residual perturbation of the virus in the population. This persistent presence suggests potential seasonality and possible outbreaks, similar to influenza, and raises concerns about long COVID [26]. Overall, these factors underscore the need for ongoing research in systems biology to broaden our understanding of SARS-CoV-2 infection.

Our research focuses on the mechanisms of immune response development, encompassing both innate and adaptive arms, and considering cellular and humoral components. The experimental data on T and B cell dynamics, viral loads, and antibody and cytokine levels were used for model calibration. Initial values of the model variables and parameters were calculated based on reliable experimental data and published literature (see Section 2.2). After the model calibration, we examined the impact of immune system aging on immune response efficiency (see Section 3.4), which resulted in three COVID-19 progression modes. The developed model was tested on a set of biological hypotheses to validate its accuracy. The model serves as a foundation for studying various pathogens, as it integrates key components of the immune system, including dendritic cells, macrophages, IL-2, IL-6, IL-12, IFNγ, T and B cells, and three classes of antibodies. It can be adapted to specific pathogens by incorporating corresponding experimental data and is particularly relevant for respiratory viral infections.

In addition, we pursued a goal to construct a module designed to align with the Immune Digital Twin (IDT) paradigm [27]. The IDT is a digital representation of a real patient, enabling personalized therapy and in-depth exploration of immune response mechanisms. Although this concept remains largely futuristic at present, existing research has provided encouraging evidence that supports the feasibility of such an approach and offers promising prospects for the future of Digital Twins. Advancements have already been achieved in fields like oncology, cardiology, and pancreatology [28,29,30,31,32,33]. In addition, attempts have been made to model the immune response within the Digital Twin paradigm [34]. We adhered to the foundational principles of IDT development, as outlined in relevant reviews: findability, accessibility, interoperability, and reusability (FAIR) [35,36].

Our model was developed using the open-source BioUML platform, adhering to standards such as the Systems Biology Markup Language (SBML) and Systems Biology Graphical Notation (SBGN). Additionally, it was implemented in Julia, a widely used programming language for computational modeling. All associated files and documentation are freely available in a GitLab repository (see. Section 2.1). The modular design of our framework allows it to function as a standalone component or be integrated into more complex models. It can also serve as a foundation for further extensions, e.g., adding new compartments (such as blood module) or new details (such as Treg cells).

## 2. Materials and Methods

### 2.1. Mathematical Model

To construct a complex multi-scale mathematical model of the immune response to SARS-CoV-2 infection, we employed a modular approach previously that had been used for building various mathematical models [37,38], along with model manual extension and adjustment. At the first step, we integrated manually reviewed and validated models of the immune response to Mycobacterium tuberculosis (MT) [39] and influenza A (IA) virus [14] infections. Both models are two-compartmental and include the lungs and draining lymph nodes as separate compartments. The MT model focuses on the innate immune response, highlighting the roles of antigen-presenting cells and T helper cells, while the IA model predominantly emphasizes adaptive immunity, providing a detailed description of both humoral and cellular components.

The MT model, developed by Marino and Kirschner, describes the infection of resting macrophages ($M_{R}$) in the lungs by bacteria ($B_{T}$), which are categorized into external ($B_{E}$) and internal ($B_{I}$) types. The bacteria trigger the activation of immature dendritic cells (IDC) and resting macrophages, leading to their differentiation into mature dendritic cells (MDC) and activated macrophages ($M_{A}$), respectively. MDCs then drive the activation of naïve T cells ($T_{naive}$) to form precursors ($T_{P}$) in the lymph nodes. It should be noted that T cell species combine both cytotoxic and T helper functions. In the lungs, T cell precursors differentiate into T helper 1 and 2 types (${Th}_{1}$, ${Th}_{2}$), which orchestrate cytokine production and contribute to the elimination of infected macrophages. At the same time, cytokines act as regulatory factors for the vast majority of immune response reactions.

The IA model, built by Lee and co-authors, simulates the infection of epithelial cells ($E_{P}$) in the lungs by the influenza virus (V). Infected epithelial cells (${EP}_{I}$) begin to produce viral particles, which activate IDC, leading to their transport to the lymph nodes, where they mature into mature dendritic cells (MDC). They drive the activation of naïve T helper cells ($H_{N}$), naïve cytotoxic T cells ($T_{N}$), and naïve B cells ($B_{N}$), resulting in their proliferation into effector cells ($H_{E}$, $T_{E}$, and $B_{A}$, respectively). Effector T helper cells regulate the differentiation of B cells into short-lived ($P_{S}$) and long-lived ($P_{L}$) plasma cells. The latter produce antibodies (A) that eliminate free viral particles in the lungs. Eventually, effector cytotoxic T cells migrate to the lungs and eradicate infected epithelial cells.

**Figure 1:**
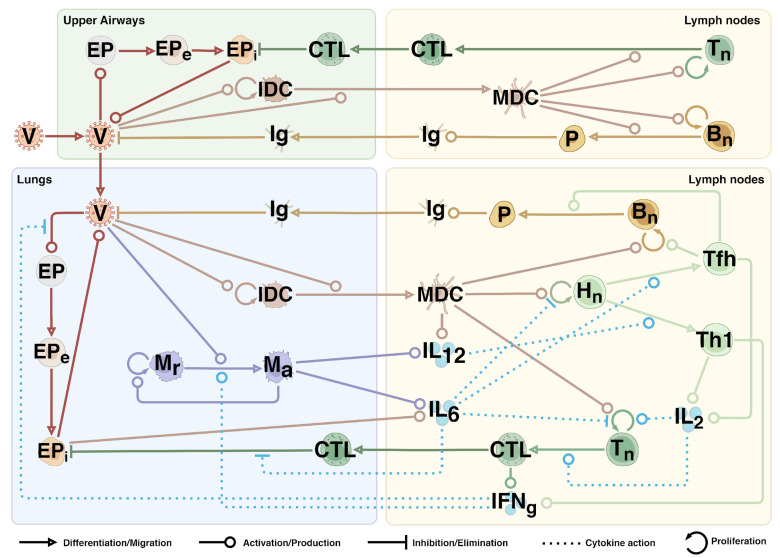
A schematic representation of the built model. The process notations are provided below the diagram. V—virus, EP—epithelial cells (e—exposed, i—infected), DC—dendritic cells (I—immature, M—mature), M—macrophages (r—resting, a—activated), Hn—naïve CD4+ T cells, Tn—naïve CD8+ T cells, Bn—naïve B cells, Th1—T helper 1 type cells, Tfh—T follicular helper cells, CTL—cytotoxic T cells, P—plasma cells, Ig—immunoglobulins (IgA, IgM, IgG), IL—interleukins (2, 6, 12), IFNg—interferon gamma.

Based on the mentioned models, we have developed an extended model consisting of four compartments: the upper airways, lungs, and the corresponding lymph nodes for each, representing the key areas involved in respiratory infection (Figure 1). The model includes a total of 35 differential equations and 112 parameters. Its complete and detailed description is provided in Text S1. The constructed modular model is also available on GitLab at https://gitlab.sirius-web.org/virtual-patient/modular-immune-system (accessed on 1 March 2025) and in the BioModels repository under identifier MODEL2503040001. The model is part of the complex “Virtual Patient” project, which aims to develop a digital twin of a real patient. Currently, models of the cardiovascular system, epilepsy, and muscle metabolism have been developed. These models are also available on GitLab. To address the common issue of model reproducibility in systems biology [40], we additionally reproduced the model using Julia programming language and conducted all equivalent investigations. The Julia version is also available on GitLab.

### 2.2. Experimental Data

To accurately reflect biological reality and successfully verify the model, we used various experimental data, including time-series dynamics of cells and molecules, calculated or estimated parameters, and the initial values for model entities. Some parameter values were taken from existing mathematical models [13,14,21,22,23,39] and are labeled as “sourced.” Other parameters were estimated through model fitting or were independently calculated using constants from the published data. Corresponding references and a brief description of each parameter type are provided in Table S1.

#### 2.2.1. Initial Values

To calculate the number of epithelial cells, we followed two assumptions. First, we considered only those cells that are susceptible to infection and simultaneously express both ACE2 and TMPRSS2 receptors required for coronavirus entry [41,42]. Although the exact distribution of these receptors is difficult to determine, approximate data suggest that only about 1% of cells co-express both receptors and may therefore be infected [43]. Second, we based our estimates on the spatial distribution of cells. We initially assessed the epithelial surface area of the upper airways, including the nasal and oral cavities and the pharynx. The approximate surface areas are as follows: nasal mucosa—170 cm^2^ [44], oral mucosa—200 cm^2^ [45,46], and pharynx, which is composed of the oropharynx, nasopharynx, and hypopharynx—150 cm^2^.

Since complete measurements for the pharynx are unavailable, we roughly estimated its surface area by considering the pharynx as a cylinder without bases. With an approximate length of 13 cm [47] and a lumen diameter of 2 cm [48,49], the pharynx has an estimated surface area of 150 cm^2^. Calculations for individual parts of the pharynx, such as the nasopharynx, verified this estimate and align with experimental data. In particular, the nasopharynx has a length of 4 cm and a lumen diameter of 2 cm [49], yielding an area of about 50 cm^2^, which corresponds to known measurements [50].

Overall, the upper airways have an approximate surface area of 500 cm^2^. Given that the density typical for airway epithelium ciliated cells is roughly 700,000 cells per cm^2^ [51,52], and considering only susceptible cells, we estimated the number of epithelial cells to be around 5.2 × 10^6^ cells. This result is consistent with a similar calculation using the experimentally derived density of susceptible cells in the airway epithelium (approximately 10,000 cells per cm^2^ [53]), which yields about 5.5 × 10^6^ cells. Due to the difficulties in precise measurements, we opted for the higher estimate and used the initial count of 5.5 × 10^6^ susceptible epithelial cells in the upper airways.

We considered the trachea, bronchial tree, and lungs as the lower airways. According to cell counts provided by Hatton and colleagues [54], the approximate total number of ciliated, goblet, and alveolar (both AT1 and AT2) epithelium cells in the lower airways ranges from 3 × 10^10^ to 7 × 10^10^. Taking into account the proportion of vulnerable cells [43], we estimated the initial number of susceptible cells in the lower airways to be 5.5 × 10^8^. We considered both type I (AT1) and type II (AT2) alveolocytes due to emerging evidence about SARS-CoV-2 cell tropism to both of them, which was not clearly understood early in the pandemic [55,56]. Furthermore, we did not account for variability in ACE2 receptor expression, which is influenced by cell type, location, and developmental stage. However, this may potentially affect COVID-19 progression and severity to some extent [57,58].

To determine the initial number of dendritic cells (DCs), we used the known density of DCs in the airway epithelium, which varies widely from roughly 50,000 to 80,000 cells per cm^2^ of the epithelium [59,60,61]. We opted for the lower bound because several subpopulations of DCs are usually distinguished [62], yet the main antigen-presenting function is performed primarily by myeloid dendritic cells [63]. Given the surface area of the airway epithelium is around 500 cm^2^, we calculated the total number of dendritic cells residing in the epithelium to be about 2.5 × 10^7^. To find the concentration of DCs, we first estimated the total volume of the epithelium in the upper airways. With an epithelial thickness ranging from 50 to 300 μm, and a mean value of 200 μm [64,65,66], the total volume is approximately 10 mL. This results in a dendritic cell concentration in the upper airways of 2.5 × 10^6^ cells per mL.

Due to the limited understanding of dendritic cell concentrations in the lungs, we performed calculations similar to those used for the upper airways. The lung surface area (excluding the bronchi) is approximately 70 m^2^, or 7 × 10^5^ cm^2^ [67]. The surface areas of the bronchi and trachea are 2.5 × 10^3^ cm^2^ and 2.4 × 10^3^ cm^2^, respectively. Assuming an average alveolar thickness of 0.3 μm [68] and a mean thickness of 50 μm [65,66,69] for the bronchi and trachea, we estimated the total lung volume to be 50,000 mL. Given that myeloid DCs (mDCs) are the predominant subpopulation in the lungs [62], we opted for the upper bound density of 80,000 cells per cm^2^ of the epithelium [59,60,61]. Thus, we estimated the total number of DCs to be approximately 6 × 10^10^ cells, resulting in a concentration in the lungs of 1.2 × 10^6^ cells per mL.

Given that the known total number of macrophages in the lungs is 2 × 10^10^ cells [54] and taking into account that the volume of the lower airway epithelium is 50,000 mL, we estimated the concentration of alveolar macrophages to be 4 × 10^5^ cells per mL. It is worth noting that the estimated total number of macrophages is also supported by calculations based on a lung mucosal surface area of 100 m^2^, resulting in approximately 1.2 × 10^10^ macrophages, assuming a mean density of 20,000 cells per cm^2^ [70,71].

To accurately determine the initial concentrations of T and B cells, it is essential to consider the lymph nodes, which serve as reservoirs for lymphocytes. First, we evaluated the volume of the lymph nodes draining the upper and lower airways. For the upper airways, which include the head and cervical lymph nodes, the number ranges from 300 to 400 nodes [72,73], with an average volume of 0.3 mL per node [74,75]. This results in a total volume of lymph nodes in the upper airways ranging from 90 to 120 mL. We opted for 90 mL, based on other research indicating a lower mean volume for these nodes [76]. In contrast, the number of lymph nodes draining the lower airways [77], including the lungs, is much smaller and equals approximately 100 nodes [78]. However, they are larger, with an average volume of about 2 mL each [79]. Thus, we estimated the total volume of the lymph nodes draining the lower airways to be around 200 mL.

To evaluate the initial concentrations of naive B and T cells, we used the total number of lymphocytes in the lymph nodes [54], along with the estimated volumes of these nodes. For the lymph nodes in the upper airways, with a total volume of 90 mL, we calculated the concentrations of naive B cells and CD8+ T cells to be 3.3 × 10^4^ cells/mL and 1.6 × 10^4^ cells/mL, respectively. In contrast, for the lymph nodes draining the lower airways, which have an estimated volume of 200 mL, the concentrations were found to be 6 × 10^4^ cells/mL for naïve B cells, 1.0 × 10^5^ cells/mL for naïve CD4+ T cells, and 3.3 × 10^4^ cells/mL for naïve CD8+ T cells. It should be noted that these calculations are based on the proportion of specific naive lymphocytes, which constitute approximately 0.01% of the total pool for B cells [80] and 0.0001% for T cells [81,82].

SARS-CoV-2 is known to spread either through respiratory or airborne routes [83]. The estimated dose transmitted via these pathways varies from several hundred to several thousand virions [84,85]. We selected a value of 1000 virions, which is notably higher than the minimum infectious dose for SARS-CoV-2 of 100 virions [86] and aligns closely with the average infectious dose.

#### 2.2.2. Time-Series Data

**Figure 2:**
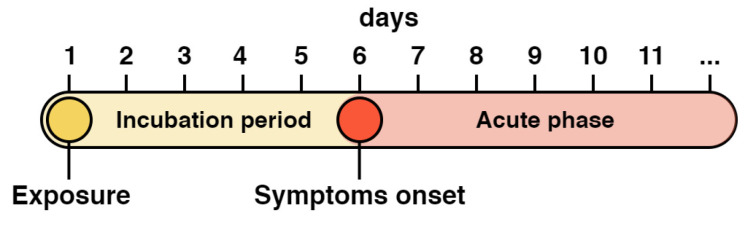
A timeline of the disease progression following exposure to the SARS-CoV-2 virus.

To accurately model the dynamics of viral load in the upper airways, we used time-series data provided by Killingley from a controlled study [87], where volunteers were intranasally inoculated with a wild-type SARS-CoV-2 (SARS-CoV-2/human/GBR/484861/2020). Apart from the opportunity to investigate the infection development from the very beginning, we also had the chance to consider an incubation period, thereby enhancing the biological accuracy of the model. To implement this, we added an average incubation period of 6 days [88,89] to the dynamic experimental data for all entities in the lungs, including the virus and lymphocytes. This adjustment accounts for the fact that the first and subsequent measurement points are typically recorded after the onset of symptoms, which occurs following the incubation period (Figure 2).

Based on selected experimental data, the median peak of viral load in the nose, which represents the upper airways, occurred on the 9th day, with a median of 1.7 × 10^6^ RNA copies/mL. The highest viral load in the approximated data was 9.0 × 10^7^ and occurred on the 6th day. These values are consistent with those observed by Lui and colleagues [90], who estimated the median peak to be on the 9th day, with a median viral load of 2.5 × 10^6^ RNA copies/mL. Table S3 provides additional details and other experimental data.

To model viral dynamics in the lower airways, we used data on viral load in sputum by Wölfel and colleagues [91]. While there is no specific information about the viral strain, the research was conducted near the end of 2020, so we can assume it was one of the dominant strains in the region at that time, such as the Alpha variant [92]. The median peak in the data occurred on the 14th day, with a median viral load of 3.7 × 10^6^ RNA copies/mL. The highest value, 1.2 × 10^8^, was observed on the 11th day. This also corresponds to the theoretical maximum value of viral load in the lungs, estimated by Sender’s group [93].

To accurately model antibody dynamics, we fit the model to measurements of three immunoglobulin classes (IgM, IgA, IgG) specific to the spike protein of the early SARS-CoV-2 strain (GenBank: MN975262) [94], which is known to be the main target for neutralizing antibodies [95]. Using the LOESS function to approximate the raw data, we observed that IgM peaked first on the 27th day after infection, with a maximum value of 15.4 μg/mL. IgA and IgG reached their maximum levels later, on the 29th day and 33rd day, with the highest value being approximately 16.2 μg/mL and 18.5 μg/mL for IgA and IgG, respectively.

We adjusted the T cell dynamics predicted by the model to match experimental data from a retrospective study of HIV/SARS-CoV-2 coinfection, focusing only on the healthy control group, as well as data from a longitudinal study of COVID-19 patients conducted by Bergamaschi and colleagues [96,97]. The studies did not provide any information about SARS-CoV-2 strains, but since both were conducted in the summer of 2020, we hypothesize that they involved one of the earliest viral strains [92]. According to the results, CD8+ T cells reached their peak approximately on the 28th day after infection, with a value of 1.1 × 10^5^ cells/mL. Similarly, CD4+ T helper 1 type cells peaked on the 21st day after exposure to the virus, with a maximum value of 4.1 × 10^5^ cells/mL. Additionally, to fit the model more accurately, we established upper boundary for biologically reliable T cell concentrations during moderate COVID-19 progression, setting it at 10^6^ cells/mL for both CD4+ and CD8+ T cells [98,99,100,101].

Given the limited availability of time-series data for B cells, we established an upper boundary for the maximum biologically reliable concentration of B cells at 10^6^ cells/mL [101], similar to the approach used for T cells. We suggested that the proper dynamics of B lymphocytes is indirectly ensured by fitting the model to the previously described data on immunoglobulin dynamics.

Due to the temporary nature of most cytokines, obtaining appropriate time-series data on their dynamics can be challenging. We used experimental data for IL-6, a central pro-inflammatory cytokine during COVID-19 [102]. As with sputum viral load and T cell dynamics, the authors did not specify the viral strain. However, since the study was carried out in the spring of 2020, we assumed it involved one of the dominant strains at that time [92]. According to the data, the IL-6 level peaked on the 13th day post-infection, reaching a maximum of 170 pg/mL. This value also falls within the biologically plausible range, which spans approximately from 0 to 500 pg/mL [103].

#### 2.2.3. Parameter Selection

We divided the entire set of 112 parameters into 3 groups: 42 calculated parameters, 59 estimated through optimization methods, and 11 collected from other mathematical models or identified during experiments (Table S1).

Among the easiest parameters to calculate are the degradation rates of cells and molecules. We used the half-life of the entity to calculate the elimination rate constant k (Equation (1)), derived from the classical half-life formula [104,105,106].

(1) $$t^{1/2}=\frac{0.693}{k}\;\;\;\;\;\to \;\;\;\;\;k=\frac{0.693}{t^{1/2}}$$

Plasma cells are known for their high productivity, with each releasing approximately 10 to 1000 antibody molecules per second [107,108,109,110,111,112]. The number of secreted antibodies depends on factors such as the immunoglobulin class, the type of plasma cell, the microenvironment, and others [113]. In our calculations, we used immunoglobulin G as the reference molecule and assumed a fixed concentration of 100,000 active plasma cells per milliliter. This value approximates the mean number of short-lived plasma cells typically observed during an infection [101].

Considering the weight of one IgG molecule to be 150,000 Da [82,114,115], we converted the initial range of antibody secreted by plasma cells to 2.15 × 10^−7^ to 2.15 × 10^−5^ μg per cell per day. We then evaluated the antibody secretion rate for plasma cells per mL (using an assumed concentration of 1 × 10^5^ cells per mL), resulting in a range of 0.02 to 2.0 μg per cells/mL per day. To account for antibody dilution in the blood (5000 mL), given that experimental data reflect serum antibody concentrations, we obtained a final range of 4.0 × 10^−6^ to 4.0 × 10^−4^ μg/mL per cells/mL per day. We selected the lower bound of this range, as it enables the precise replication of the antibody dynamics demonstrated in the experimental data. Subsequently, we fine-tuned the values to ensure compatibility across various antibody classes, resulting in 6.0 × 10^−6^ μg/mL per cells/mL per day for $r_{{IgG}_{P}}$, 7.0 × 10^−6^ for $r_{{IgA}_{P}}$, and 7.0 × 10^−6^ for $r_{{IgM}_{P}}$.

To evaluate the rate of virion release by infected epithelial cells ($r_{V_{EPi}}$), we first considered the burst size of SARS-CoV-2, defined as the number of virions produced by a single infected cell over its lifetime, ranging from 10 to 10,000 virions [93,116,117]. We then calculated the lifetime of infected cells based on their decay rate (0.5 day^−1^), obtaining an approximate life duration of 20 h. This calculation yielded a range of 12 to 12,000 virions released per day. Since only about 0.01% of virions are indeed infectious [118,119,120], we initially opted for a rate of 1000 virions per day and then adjusted for the infectious portion, resulting in a final rate of 10 infectious virions per day.

For parameters derived from other models or manually calculated, we estimated confidence intervals (CIs) based on the assumption of their normal distribution. The initially wide confidence intervals obtained for manually calculated parameters were further constrained to more stringent boundaries to ensure stable solutions, as it was observed that physiologically justified intervals hindered the stability of the model simulations.

Since in most cases the standard deviation for sourced parameters was not available, we assumed it to be 10% of the parameter value. Based on this assumption, confidence intervals capturing 95% of the potential parameter values were calculated.

All parameters and their corresponding CIs are provided in the Supplementary Materials (Table S1).

### 2.3. Numerical Simulation

The model was solved using the Java Variable-Coefficient ODE (JVODE) solver, which is a Java implementation of the CVODE solver originally written in C [121]. JVODE is integrated into BioUML, the open-source computational platform for systems biology that we used for model development [122]. In addition to ODEs, the model incorporates delay differential equations (DDEs) to account for time delays, which are crucial for accurately modeling migration processes like the transport of dendritic cells from the lungs to the lymph nodes. Furthermore, to take into consideration population heterogeneity in immune responses and individual immune system characteristics, we utilized a Monte Carlo-based approach to estimate uncertainty intervals for model trajectories [123]. In particular, parameter values were randomly sampled from their respective confidence intervals, and the model was simulated for each set of sampled parameters. Overall, we conducted 10,000 simulations to ensure that the analysis adequately captured the uncertainty. To establish confidence intervals for the model trajectories, we selected the 5th and 95th percentiles of the simulation outputs, defining a 90% confidence interval. This approach implies that the obtaining intervals reflect both the inherent variability of the parameters and the population heterogeneity captured by the model.

Since a substantial number of parameters could not be directly derived from experimental data or calculated, optimization methods were essential for refining the model and improving its predictive performance. Initially, we manually adjusted the model parameters to align the model dynamics with the experimental data (see Table S3). Then, we fitted the entire adjusted model (encompassing all compartments simultaneously) to the experimental data for moderate COVID-19 progression, which is detailed in Section 2.2. The experimental data were approximated using polynomial functions, except for antibodies, for which the LOESS approximation results from the original publication were used, to construct a smooth curve capturing the main trend in the data (see Section 3.3). To identify the subset of parameters to tune, we conducted a sensitivity analysis (see Section 2.4) and selected the most significant unknown parameters.

We employed three optimization algorithms implemented in the BioUML platform, including the stochastic ranking evolution strategy—SRES [124], the multi-objective particle swarm algorithm—MOPSO [125,126], and the multi-objective cellular genetic algorithm—MOCell [127,128]. We conducted three runs for each algorithm and selected the best solution from each method based on the sum of squared errors as the objective function during the fitting process. The overall best result was chosen according to the three best solutions from the optimization methods. To address the varying scales of the variables, weights were applied to ensure balanced contributions of all variables to the optimization process. To incorporate biological constraints, we also added a penalty term based on the squared penalty for constraint violation. This resulted in minimizing the following function (Equation (2)):

(2) $$\underset{x}{\min}(\chi^{2}+\lambda \sum_{i}max{\left(0,\;h_{i}\left(x\right)\right)}^{2})$$

where $\chi^{2}$ is the objective function, $h_{i}(x)$ represents the constraint for the variable x, and λ is the penalty coefficient controlling the influence of the constraint violations.

To compare the solutions obtained through various optimization methods, we calculated a modified Akaike information criterion (AIC) [129]. Since the analysis focused on variations of the same model, where only parameter values differed and the number of estimated parameters K remained constant, K was excluded from the calculation (Equation (3)):

(3) $$AIC=\ln\left(\chi^{2}\right)+\lambda *\ln\left(P\right)$$

Here, $\chi^{2}$ represents the objective function, P is the penalty function, and λ is a weighting coefficient determining the contribution of the penalty term. In our calculations, we set λ=0.5.

### 2.4. Sensitivity Analysis

To evaluate the impact of various parameters on the progression of COVID-19, we performed a local sensitivity analysis [130]. We used the cumulative viral load throughout the course of infection as a measure of COVID-19 severity:

(4) $$V_{AUC}=\int_{T_{1}}^{T_{2}}V\left(t\right)dt,$$

where $T_{1}$ represents the time of virus appearance and $T_{2}$ represents the time of complete virus clearance.

To calculate the local sensitivity indices, we computed partial derivatives with respect to the vector of model parameters using the finite difference approximation method, which was implemented in the BioUML environment:

(5) $$\frac{\partial V}{\partial \alpha_{j}}=\frac{V\left(a_{j}+∆a_{j}\right)-V(a_{j})}{∆a_{j}},$$

where $∆a_{j}$ represents a small perturbation to the local parameter $a_{j}$, and $V\left(a_{j}+∆a_{j}\right)$ and $V\left(a_{j}\right)$ correspond to the solutions of the algebraic systems $f\left(V,\;a_{j}+∆a_{j}\right)=0$ and $f\left(V,\;a_{j}\right)=0$, respectively.

Due to the variability in model parameters spanning eighteen orders of magnitude, we normalized the obtained values by multiplying them by a normalization factor $\frac{a_{j}}{V}$. The sensitivity analysis was conducted on a model fitted to moderate COVID-19 progression.

### 2.5. Identifiability Analysis

To determine the uncertainties and establish confidence intervals for the fitted model parameters, we conducted an identifiability analysis using the method proposed by Raue and coauthors [131], which is based on the estimation of parameter profile likelihood. In this approach, the parameter of interest is varied incrementally, and for each fixed value, the remaining parameters are re-optimized to minimize the objective function. The points where the profile likelihood met the threshold value were used to define the bounds of the confidence interval. The threshold $\chi_{threshold}^{2}$ was set to be 5% above the minimum value of $\chi_{min}^{2}$, which was determined as the objective function value obtained from the best optimization solution.

A parameter is considered practically identifiable if it has a unique minimum in the likelihood function and a finite confidence interval. If a minimum exists, but the likelihood-based confidence region extends infinitely in one or both directions, the parameter is classified as partially identifiable. A flat or nearly flat profile likelihood indicates the non-identifiability of the parameter, suggesting that the experimental data do not provide sufficient information to estimate its value.

## 3. Results

### 3.1. Local Sensitivity Analysis

**Figure 3:**
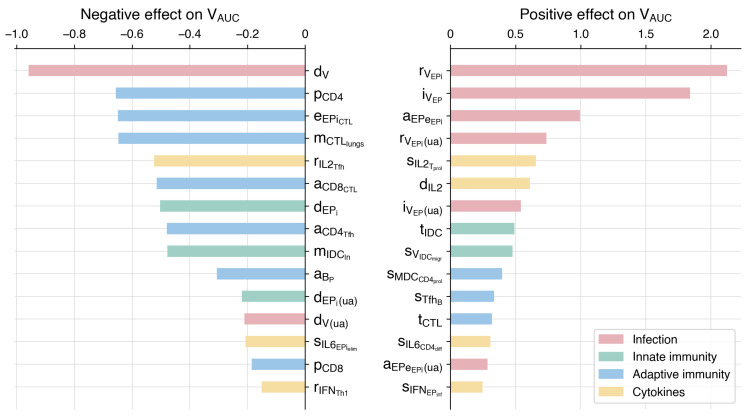
Scaled sensitivity coefficients that have negative (**left**) and positive (**right**) effects on the cumulative viral load.

To assess the model’s response to parameter perturbations, we performed a local sensitivity analysis and split the resulting values into two groups: those that negatively affect *V_AUC_* (Section 2.4) and those with a positive influence. The values were ranked, and the first quartile was selected for each group, with both groups collectively containing the 30 most influential parameters out of a total of 112 parameters. These parameters were then categorized into four groups based on their biological roles: infection, innate immunity, adaptive immunity, and cytokines (Figure 3). The analysis results for the remaining parameters are provided in Table S4.

We found that the cumulative viral load, which represents the total amount of virus over the course of infection (i.e., the area under the viral load curve), positively correlates with parameters primarily associated with the virus and the infection process. These include the rate of virion release by infected epithelial cells ($r_{V_{EPi}}$) in both the upper airways and the lungs, the rate of epithelial cell infection by the virus ($i_{V_{EP}}$), and the parameter $a_{{EPe}_{EPi}}$, which is related to the incubation period of infected epithelial cells. On the other hand, a negative correlation was observed with parameters associated with the immune response. The most significant parameters are related to adaptive immunity, including the rate of naïve CD4+ T cell proliferation ($p_{CD4}$), the elimination of infected cells by CTLs ($e_{{EPi}_{CTL}}$), and the migration rate of CTLs to the lungs ($m_{{CTL}_{lungs}}$). Another crucial parameter pertains to the maturation and migration of immature dendritic cells to the lymph nodes ($m_{{IDC}_{ln}}$), emphasizing the role of innate immunity. Collectively, these findings highlight the importance of both innate and adaptive components of the immune response in regulating the progression of the infection.

**Figure 4:**
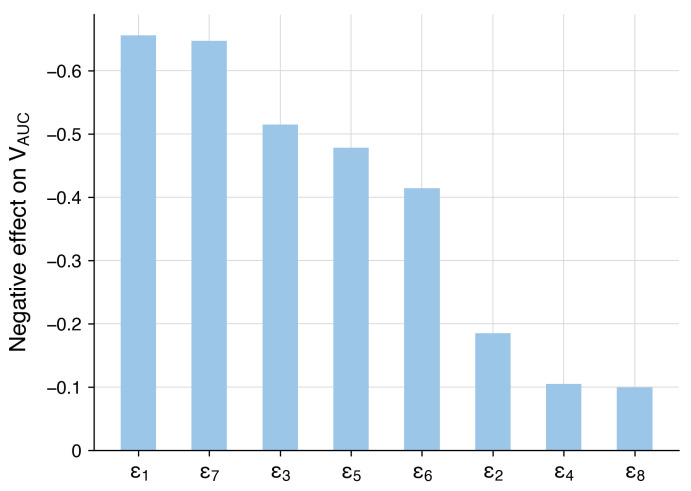
Scaled sensitivity coefficients for ***ε*** parameters for cumulative viral load.

Additionally, we assessed the sensitivity coefficients for epsilon parameters, which determine the efficacy of age-related processes (Figure 4). Details on the epsilon parameters are provided in Section 3.3. As expected, a decrease in the values of epsilon parameters, reflecting aging, resulted in increased viral load. The most significant parameters are related to the proliferation of naïve CD4+ T cells ($\varepsilon_{1}$), the elimination of infected epithelial cells by CTLs ($\varepsilon_{7}$), the differentiation of naïve CD8+ T cells to CTLs ($\varepsilon_{3}$), and the activation, migration, and maturation of immature dendritic cells ($\varepsilon_{5}$).

### 3.2. Parameter Identifiability Analysis

The studied parameters were classified into four groups based on the results of the identifiability analysis: fully identifiable, left-identifiable, right-identifiable, and non-identifiable. Out of the 59 analyzed parameters, 37 were fully identifiable, while 8 and 13 were left- and right-identifiable, respectively. Since the identifiability is relevant only for unknown parameters, we considered exclusively those estimated through model optimization (59 of 112 parameters). Notably, the analysis results for $e_{V_{IgA}}$, $e_{V_{IgM}}$, and $e_{V_{IgG}}$ were consolidated into a single variable $e_{V_{Ig}}$, as they yielded identical values. One parameter, $S_{V_{{IFN}_{prod}}}$, was classified as non-identifiable. This outcome is consistent with its role in the model: The parameter primarily controls the decay of interferon after the elimination of the virus. Since this process occurs when the main immune response is no longer active, the parameter does not influence the fit to experimental data and, therefore, is classified as non-identifiable.

Upon closer examination of the analysis results, we observe that the lung compartment (including the corresponding lymph nodes) contains 33 fully identifiable parameters out of a total of 41, while the upper airway compartment has only 4 fully identifiable parameters out of 18. However, most of the parameters in the upper airways are either left- or right-identifiable (14 out of 18), which suggests a lack of sufficient experimental data for this compartment, as we only have data for viral load dynamics in the corresponding compartment. The similarity in the model structure between the upper airways and the lungs—where most parameters are identifiable—supports the assumption that the lack of data is the primary cause for the limited identifiability in the upper airways. Since the main purpose of the upper airways is to model the correct viral entry into the lungs, the partial identifiability of parameters in this compartment is not a major concern. The upper airways serve a supporting role in the overall model, which is primarily focused on capturing the initial stages of the viral entry, which subsequently affect the dynamics in the lungs. On the other hand, the lungs module is crucial in the model, with the majority of the analysis focused on it.

Due to the non-identifiability of the parameters in one direction, the confidence intervals for partially identifiable parameters were determined by using two values: the point where the profile likelihood intersects the threshold of the objective function on one side, and the last value before the likelihood function flattens or changes insignificantly. Beyond this point, further changes no longer affect the fit.

In the lung compartment, including the corresponding lymph nodes, 80% of the parameters were found to be fully identifiable, while the remaining parameters were partially identifiable. In addition to this, the sensitivity analysis revealed a total of 30 parameters with the most significant impact on the dynamic behavior, 22 of which were estimated through the model fitting. The subsequent identifiability analysis showed that 86% of these parameters are fully identifiable. This demonstrates a high level of confidence in the parameter estimates, highlighting both the robustness of the model and the adequacy of the experimental data. A summary table of the analysis results, along with the corresponding profile likelihood plots, are provided in the Supplementary Materials (File S2).

### 3.3. Baseline Model and Optimization

**Figure 5:**
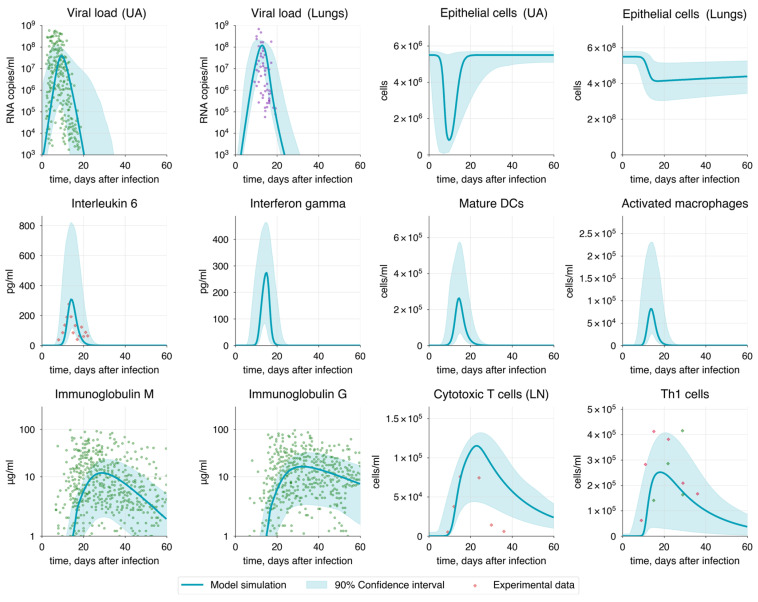
Baseline solution of the model. Lung variables are plotted, except for epithelial cells and viral load, which are shown for both compartments: upper airways (UA) and lungs. Extended versions of all subsequent plots are given in File S3 (Figures S1 and S2). The blue shaded area represents the 90% confidence interval, while dots are experimental data ([84,88,91,93,94,99]) and the curve is the simulation result.

To select the optimal solution, we compared the Akaike information criterion values computed for each optimization method, as well as for the initial manually adjusted model (Table 1):

Based on the results, the solution obtained using the MOPSO method was selected. This set of parameter values provided a stable and biologically plausible solution, with most parameters being identifiable, which is essential for ensuring the robustness and reliability of the model predictions. This solution will be referred to as the baseline model (Figure 5). Details on the parameters and initial variable values for this baseline model are provided in Section 2.2, Tables S1 and S2.

Analyzing the baseline model solution, we observed that the peak of the viral load in the upper airways and lungs fell on the 9th and 13th days, with values of 3.95 × 10^7^ and 1.2 × 10^8^ RNA copies/mL, respectively. These results not only match clinical data but also correspond to the theoretically calculated values by Sender and colleagues [93]. The complete elimination of the virus from the body occurred by the 24th day after infection, which corresponds to the median SARS-CoV-2 clearance by the 22nd day after infection [132].

**Figure 6:**
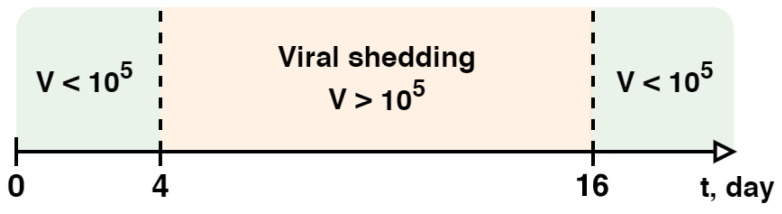
Viral shedding duration in the baseline model.

Viral shedding began about 2 days before symptom onset (on the 6th day after infection) [132,133], which corresponds to the 4th day after infection in our model. Based on this, we considered the viral load in the upper airways on the 4th day (10^5^ RNA copies/mL) as an approximate threshold for the onset and end of shedding (Figure 6). Consequently, we observed that the viral shedding period lasted from 4 to 16 days after infection, resulting in a total duration of 12 days, which is consistent with the median viral shedding duration of 11 days for moderate COVID-19 [134].

Since lung damage is a key indicator of the disease severity, we also tracked the numbers of healthy and infected epithelial cells. At the peak of the infection, the number of infected cells reached a maximum of 1.4 × 10^7^ cells, corresponding to the theoretical peak calculated by Sender and co-authors [93]. Furthermore, the total alveolar epithelial damage reached approximately 20%, which corresponds to 1.1 × 10^8^ cells. These results are consistent with the criteria for moderate COVID-19 progression (see Table 2).

The major components of the humoral immune response, IgM, IgA, and IgG, peaked on the 28th, 30th, and 32nd days after infection, with the highest values reaching 12.0 μg/mL, 14.5 μg/mL, and 16.3 μg/mL, respectively. Additionally, the seroreversion time—the period required for antibodies in the serum to completely disappear or decrease to undetectable levels—was consistent with clinical data [94], being 68, 85, and 140 days for IgM, IgA and IgG correspondingly. The growth of antibodies was driven by the development of plasma cells, which peaked on the 23nd day after infection.

CD4+ T cells peaked on the 18th and 17th days, reaching 2.5 × 10^5^ cells/mL and 1.9 × 10^5^ cells/mL for ${Th}_{1}$ and Tfh, respectively. In contrast, CTLs peaked on the 21st day, with a maximum value of 1.1 × 10^5^ cells/mL. The median values for ${Th}_{1}$ cells (1.0 × 10^5^ cells/mL) and CTLs (5.0 × 10^4^cells/mL), along with the median peak times (19th and 23rd days, respectively), closely align well with the experimental data discussed in Section 2.2.

The IL-6 levels we observed peaked on the 14th day at a maximum of 300 pg/mL. Other cytokines, IL-2, IL-12, and IFNγ, peaked on the 18th, 14th, and 15th days, with concentrations of 6.5 pg/mL, 50 pg/mL, and 270 pg/mL, respectively.

Macrophages and dendritic cells begin to activate almost immediately after the virus became detectable, bridging the innate and adaptive immunity. Activated macrophages peaked on the 14th day, with a value of 8.2 × 10^4^ cells/mL, while mature dendritic cells reached their peak on the 15th day, at 2.5 × 10^5^ cells/mL.

**Figure 7:**
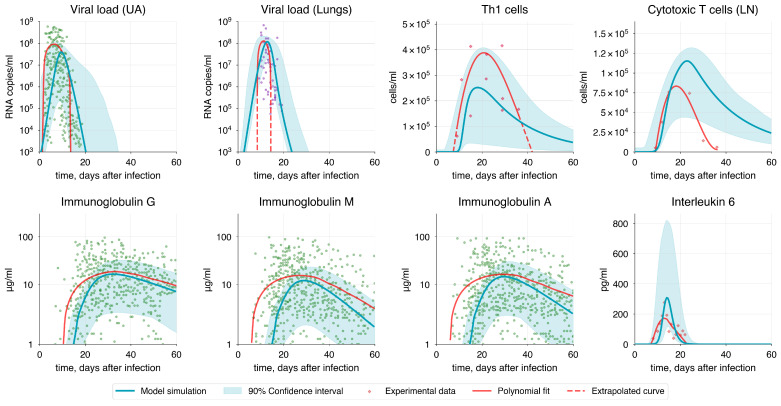
Baseline solution of the model with experimental data and approximation curves. The blue shaded area represents the 90% confidence interval. A polynomial fit is applied to all data except for the LOESS curve for immunoglobulins.

A comparison between the baseline model simulation and basic trends in the experimental data shows that the magnitude and timing of variable peaks are comparable to the approximated curves, especially considering the confidence interval of the baseline solution (Figure 7). However, due to insufficient data, it is difficult to reliably establish the proper decline curves for CTLs, ${Th}_{1}$, and V in the lungs.

### 3.4. Modes of the Immune Response During COVID-19

The progression of COVID-19 is significantly influenced by risk factors such as age, obesity, and diabetes [135]. Among these, age is shown to be a key predictor of severe or critical COVID-19 outcomes due to gradual changes in the immune system associated with aging [136,137]. Immunosenescence, the aging of the immune system, begins around age 20 and progresses, becoming significantly noticeable by age 60 [137]. Senescence affects various aspects of the immune system, with a predominant impact on adaptive immunity. It has been demonstrated that in elderly individuals the stimulation of naïve B cells by dendritic cells is reduced by up to 70%, which impairs B cell proliferation and, consequently, the formation of antibodies [138,139]. This is further complicated by age-related impairments in germinal center (GC) response, which involves reduction in both the size and the function of GCs [140]. Similar changes affect naïve T cells, leading to diminished expansion and differentiation capabilities [141,142,143,144]. Additionally, the ability of cytotoxic T cells to eliminate defective cells also declines with age [145], thereby reducing the overall efficiency of the immune response and contributing to a longer and more severe disease progression. Moreover, studies on animal models show that antibodies produced by aged mice not only have lower affinity and avidity compared to those from younger mice but also exhibit reduced protective efficacy against pathogens [146]. These findings also have been confirmed in humans [9].

However, a dramatic decline in the number of naïve T and B cells remains a primary age-related impairment of the immune system [147,148,149]. This significantly affects the body’s protective abilities due to a reduced number of antigen-specific immune cells, which are essential for mounting a robust immune response. Furthermore, immunosenescence affects not only adaptive immunity but also innate immunity. Research suggests that dendritic cells and macrophages have a diminished ability to be activated by processing antigens and to stimulate other immune cells. Additionally, impaired expression of homing factors in lymph nodes, which are essential for the transfer of antigen-presenting cells to lymph nodes, significantly reduces the migration potential of dendritic cells [141,149].

Following the National Institutes of Health guidelines [4], we categorized potential COVID-19 progressions into three modes: moderate, severe, and critical. We used the moderate progression as the baseline model, with detailed information provided in Section 3.2. Since the severity of COVID-19 is heavily influenced by risk factors mentioned earlier, we incorporated all identified age-related immune system alterations into a parameter vector $\varepsilon =(\varepsilon_{1},\;\varepsilon_{2},\;\varepsilon_{3},\;\varepsilon_{4},\;\varepsilon_{5},\;\varepsilon_{6},\;\varepsilon_{7},\;\varepsilon_{8})$ defining the effectiveness of these processes. Each parameter initially has a value of 1, representing no change, which is standard for the baseline model. Detailed description of the processes associated with each parameter is provided in Table 3. By reducing these parameters from 1 (baseline) to 0 and decreasing the initial populations of naive T and B cells in both the lungs and upper airways, we adjusted the model to simulate various disease progression scenarios by aligning it with the specified criteria for viral load, epithelial damage, and IL-6 level (Table 2).

**Figure 8:**
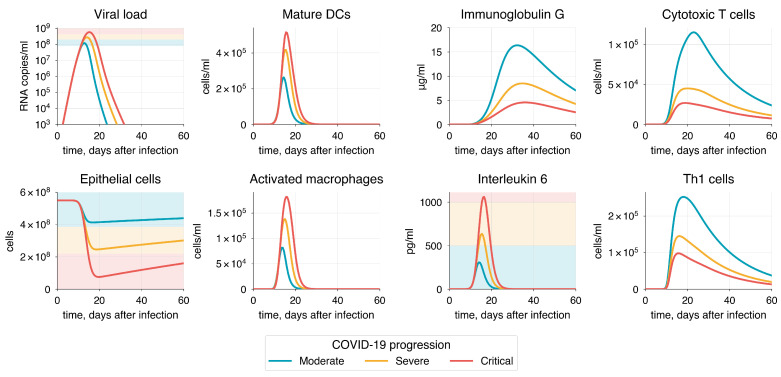
The simulation of moderate, severe, and critical COVID-19 progressions. Shaded areas in some plots indicate severity levels: green for moderate, yellow for severe, and red for critical progression.

The selected indicators (viral load, IL-6 concentration, epithelial damage) were identified as key determinants of COVID-19 severity, which is supported by numerous studies [151,152,153,154,155,156,157,158,159,160,161] demonstrating their strong correlation with disease progression, as discussed below. The simulation of COVID-19 scenarios is presented in Figure 8.

Diffuse alveolar damage (DAD) is a frequent histological hallmark of COVID-19, often necessitating mechanical ventilation and potentially leading to death [151]. This occurs because DAD disrupts normal gas exchange within the alveoli, triggering inflammation and subsequent fibrosis. Post-mortem studies suggest a high probability of fatal outcomes when lung involvement exceeds 60% [152,153]. Therefore, to differentiate disease severity, we established lung damage thresholds, as detailed in Table 2.

Studies have shown that viral load is significantly higher in patients with severe and critical conditions compared to those with moderate disease [154,155]. Severe patients typically have a viral load about two to three times greater than that of moderate cases [156,157]. Based on this, we assumed that median viral load in severe and critical states would be approximately 3 × 10^8^ and 7.5 × 10^8^ RNA copies/mL, respectively, since the highest viral load in the baseline model was 1.2 × 10^8^ RNA copies/mL. We set the viral load of 2.0 × 10^8^ and 4.3 × 10^8^ as a threshold between severity states. These values also correspond to the extent of epithelial cell infection and damage. Additionally, we considered viral shedding in relation to disease severity, as evidence suggests that patients with more severe symptoms may experience a longer duration of viral shedding compared to those with milder COVID-19 [158,159,160], as discussed in Section 3.2.

One of the most significant hallmarks of severe COVID-19 is interleukin-6, which has been shown to strongly correlate with disease severity [160]. In most cases, IL-6 levels exceeding 1000 pg/mL are associated with a higher risk of death [161]. We established the interval for the severe state based on the baseline IL-6 values specific to moderate progression and the value corresponding to critical progression (>1000 pg/mL).

To achieve the specific COVID-19 mode, we adjusted the vector of parameters ε, as previously discussed, focusing on the criteria established for each severity state (Table 4). It is important to note that achieving a particular disease progression can occur through various combinations of epsilon parameter values and the initial number of naïve T and B cells. This variability reflects the heterogeneity in the immunosenescence process among individuals in the population. Here, we propose one option for each disease severity state.

### 3.5. Validation of the Model

An essential step in further model development involves the analysis of its behavior under controlled parameter modifications and assessing its ability to provide accurate predictions in varied biological scenarios. To explore how the model predicts the viral dynamics and immune response to different conditions, such as specific cell type depletion or drug administration, we conducted several dozen simulations for each scenario (see Table 5), depending on the model’s sensitivity to specific parameters. Parameter values were incrementally changed beyond their confidence intervals, and the solutions were observed.

To evaluate the credibility of the results, we compared the mean at each time point across all experimental simulations with the baseline simulation and its standard deviation (Section 2.3). We assumed that if the mean of the experimental simulations lies outside one standard deviation of the baseline model, the effect of the parameter change is considered significant. The derived results are presented in Table 5 as follows: if a mean of the corresponding experiment deviates from the standard deviation, it is marked as either “increased” or “decreased,” with a green background indicating a positive influence on the immune response and a red one indicating a negative effect.

It should be noted that this approach has multiple limitations. These include a limited number of parameter adjustment steps, which may restrict the resolution of the analysis; confidence intervals in the baseline model that might not be sufficiently strict or accurate, potentially leading to significant changes in experiments appearing insignificant, as they remain fully within the standard deviation range; and potential biases arising from assumptions about the distribution of baseline model outputs.

Despite these constraints, this method allows for testing the model under different conditions and provides insights into its predictive capabilities for understanding and predicting COVID-19 progression and severity. Further study could refine this approach by using more rigorous confidence region definitions or incorporating advanced statistical techniques to better distinguish significant parameter-induced effects from baseline variability.

#### 3.5.1. Immune Response

**Figure 9:**
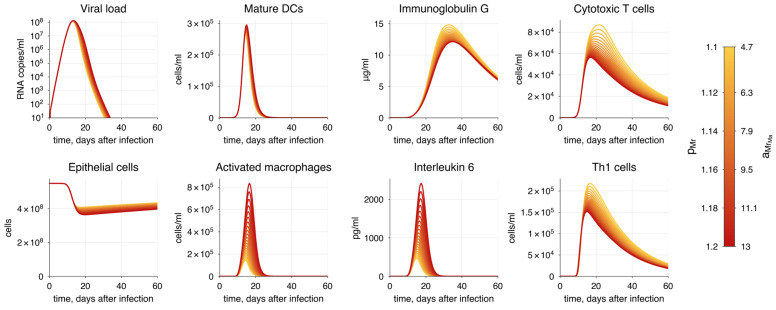
The baseline model solution with macrophage hyperactivation. The varying parameters represent the rates of macrophage recruitment and activation. The transition from yellow to red indicates increasing severity of COVID-19.

One of the most significant hallmarks of severe COVID-19 is the hyperactivation of the innate immune response, which leads to a substantial increase in the release of pro-inflammatory cytokines [162,163]. This results in prolonged inflammation and suppression of the adaptive immunity, particularly affecting T cells. Interleukin-6 (IL-6) is a key player in this process [160,164], as evidenced by the correlation between IL-6 levels and disease severity. To simulate this, we gradually increased the rates of macrophage activation $a_{{Mr}_{Ma}}$ and macrophage recruitment $p_{Mr}$ (Figure 9).

We observed an increase in the duration of viral shedding, indicating a worse disease outcome. We also noted that CTL and antibody responses are impaired by significantly elevated levels of IL-6. This suggests that macrophage hyperactivation is directly proportional to the severity of COVID-19. However, the model captures the contribution of macrophage activation to disease severity only to a limited extent, because the magnitude of viral load does not change significantly.

**Figure 10:**
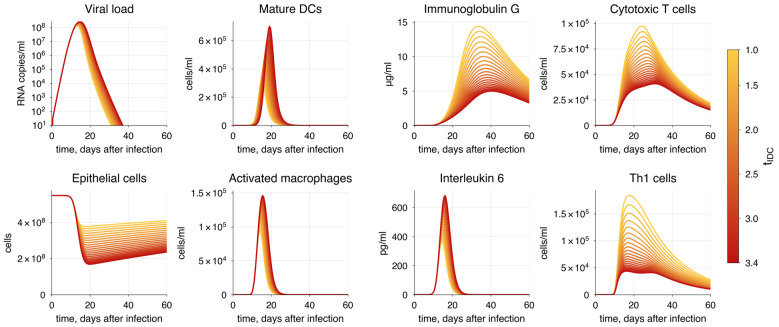
The baseline model solution with innate immune response delay. The varying parameter represents the delay of dendritic cell maturation and migration to lymph nodes. The transition from yellow to red indicates increasing severity of COVID-19.

In addition to hyperactivation of the innate immune response, a study revealed a strong correlation between COVID-19 severity and delay in the innate immune response [165]. To test this, we increased the delay in IDC maturation and migration to the lymph nodes ($t_{IDC}$) by up to 3.5 times. We observed an increased viral load and greater alveolar epithelium damage (Figure 10). Additionally, we observed a corresponding shift in the peaks of T cell and antibody responses; however, their magnitudes remained within the confidence interval. This delay enables the virus to replicate while more unhindered during the initial infection phase.

#### 3.5.2. Immunosuppression

We identified and examined two types of immunosuppression: one resulting from HIV infection and the other from immunosuppressive therapy, employed in organ transplantation and autoimmune diseases [166,167]. The first type involves the depletion of CD4+ T cells [168], weakening the adaptive immune response and making the body dramatically vulnerable to infectious agents like SARS-CoV-2. In the second case, immunosuppression is achieved through medication primarily aimed at reducing T cell proliferation, resulting in substantially lower T cell concentrations, sometimes leading to their complete depletion [169]. Studies indicate a significantly higher risk of poor COVID-19 outcomes in patients with solid organ transplantation [170,171], placing immunosuppressed individuals in a high-risk group.

**Figure 11:**
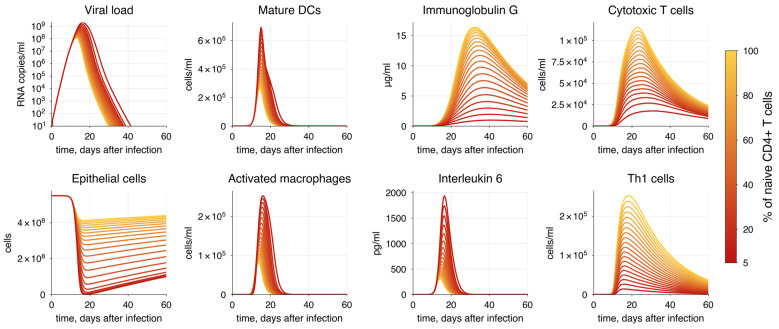
The baseline model solution with naive CD4+ T cell depletion. The transition from yellow to red indicates increasing severity of COVID-19.

To simulate the first scenario, we gradually decreased the initial concentration of naïve CD4+ T cells from 100% to 5% of the initial value. This reflects the progression of HIV infection, ultimately leading to the AIDS state characterized by the complete depletion of CD4+ T cells [172]. The simulation demonstrated a predictable increase in viral load and epithelium damage, resulting from the development CD4+ T cell lymphopenia, which affects both cellular and humoral immune responses (Figure 11). This corresponds to the research findings reporting impaired plasma cell formation and exhaustion of CD8+ T cells, which, combined with SARS-CoV-2 infection, contribute to critical conditions where the emerging immune response is insufficient to combat the virus [173,174,175]. According to the criteria for critical lung tissue damage incompatible with life (Table 3), we conclude that HIV-positive patients with a reduction in CD4+ T cell counts of more than 60% from the initial level are unlikely to survive COVID-19.

To simulate the second scenario, we chose corticosteroids as an example of a drug representing a classical strategy for immunosuppression therapy. Corticosteroids significantly impact adaptive immunity, particularly affecting T cell development [176,177], whereas they do not substantially decrease B cells [178]. Thus, we gradually reduced the proliferation rates of both CD4+ ($p_{CD4}$) and CD8+ ($p_{CD8}$) T cells.

**Figure 12:**
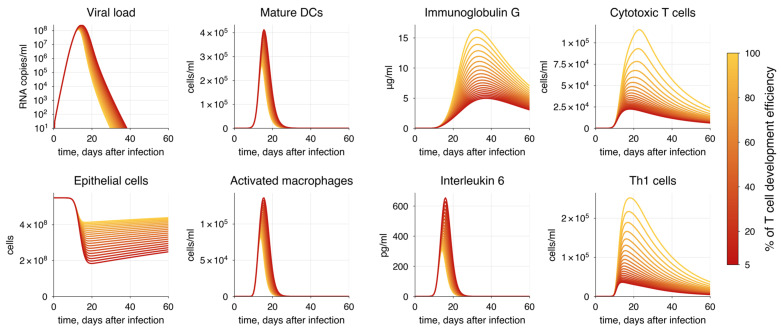
The baseline model solution with impaired T cell development due to immunosuppression. The varying parameters represent the rates of CD4+ and CD8+ T cell proliferation. The transition from yellow to red indicates increasing severity of COVID-19.

A significant reduction in T cell proliferation rates resulted in the inability of the immune system to completely eliminate the virus from the body (Figure 12). When T cell proliferation efficiency fell below 70% of initial values, it led to virus persistence and a dramatic decline in the number of alveolar cells as the immune response waned. At the same time, we observed a predictable worsening of the disease outcome, which was directly proportional to the extent of T cell proliferation impairment.

Thus, this raises important issues regarding the need for a more careful approach to immunosuppressive cases and underscores the importance of developing and implementing new treatment methods.

#### 3.5.3. SARS-CoV-2 Infectivity

Since the onset of the pandemic, many variants of SARS-CoV-2 have emerged. These new viral strains commonly exhibit increased transmissibility and lead to more severe disease compared to the wild type. Beyond that, the virus has shown the ability to evade human immunity, particularly targeting the humoral immune response, resulting in a reduction in antibody neutralization activity of more than 50% in some cases. These characteristics seem to be valid for all previously dominant SARS-CoV-2 variants—Alpha, Beta, Gamma, and Delta [179,180,181,182]—but have changed in more recent variants such as Omicron. In contrast, it reliably causes less severe symptoms [183,184] while exhibiting significantly higher contagiousness [185], which altogether allowed it to become the dominant SARS-CoV-2 variant.

Since the experimental data we utilized are not linked to a specific viral strain, we focused on analyzing the impact of varying parameters related to cell infection and antibody evasion. We treated these parameters as defining characteristics of the viral strain.

**Figure 13:**
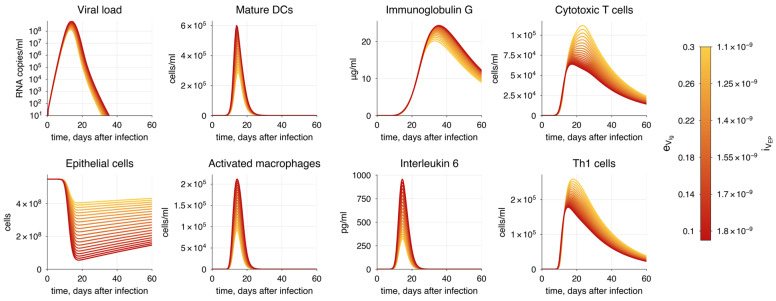
The baseline model solution with enhanced viral infectivity and immune evasion. The varying parameters represent the rates of epithelial cell infection by the virus and virion neutralization by antibodies. The transition from yellow to red indicates increasing severity of COVID-19.

We examined the concurrent increase in the epithelial cell infection rate ($i_{V_{EP}}$) and the reduction in antibody neutralization activity ($e_{V_{Ig}}$), which is related to the virus’s ability to evade immune responses. During the experiment, we observed a higher viral load and damage to the alveolar epithelium (Figure 13). Moreover, the duration of viral shedding expanded as well, indicating not only a more severe progression of COVID-19 but also enhanced virus transmissibility.

#### 3.5.4. Treatment Strategies

**Figure 14:**
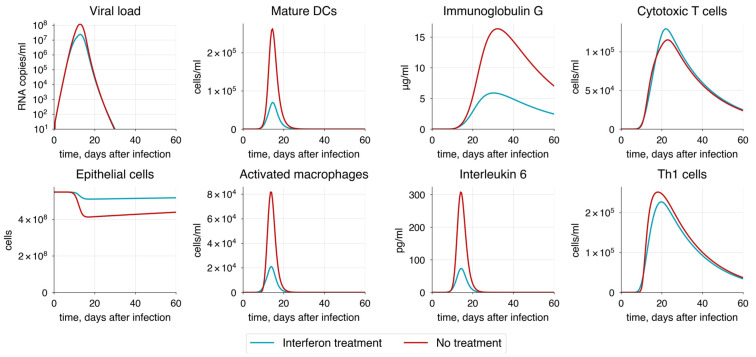
The baseline model solution with interferon administration. Interferon is administered at a concentration of 2000 pg/mL daily for five days post symptom onset.

Interferons are well known for their role as key inducers of intracellular immunity, helping cells combat pathogens before the activation of the primary immune response [186,187,188]. We implemented this mechanism through IFN-dependent inhibition of epithelial cell infection (see File S1). To study the dynamic changes in immune response resulting from IFN action, we followed a treatment protocol [189] in which patients received daily doses of interferon at a concentration of 2000 pg/mL for five days, starting immediately after the symptom onset. Compared to patients who did not receive any treatment, those who did experienced a substantial reduction in viral load and a predictable decrease in disease severity (Figure 14).

**Figure 15:**
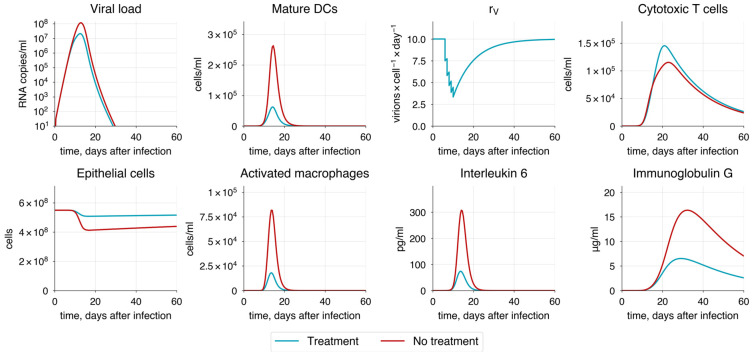
The baseline model solution with inhibited viral replication. The drug is administered daily for five days post symptom onset.

Another key target for antiviral drugs is the viral replication process, aiming to inhibit it and reduce the viral spread. Examples of such drugs include amodiaquine, atovaquone, bedaquiline, and others [190]. Using the developed model, we studied the inhibition effect of daily drug injections on viral replication within infected cells, simulating the drug administration. We assumed that the replication rate ($r_{V_{EPi}}$) changed dynamically during the course of treatment before returning to its baseline value, reflecting the temporary nature of the drug’s action. During drug administration, we observed a significant reduction in viral load and damage to epithelial cells, demonstrating the high efficacy of such drugs and their potential for treating COVID-19 (Figure 15).

## 4. Conclusions

Herein, we present a developed modular model of the human immune response to SARS-CoV-2 infection. The model structure consists of two main parts: the upper airways (including the nasal cavity, oral cavity, and pharynx) and the lower airways (including the trachea, bronchial tree, and lungs). Each structural module is connected to a draining lymph node compartment, overall forming four interconnected modules. The model incorporates innate immunity, involving macrophages and dendritic cells, and adaptive immunity, comprising humoral (antibodies) and cellular (CTLs, Th1, and Tfh cells) components. Cellular interactions in the model are facilitated by direct contacts such as antigen presentation or by cytokine signaling via IL-2, IL-6, IL-12, and IFNγ.

To estimate the model parameters, we used time-series clinical data on moderate disease progression, including concentrations of IL-6, CD4+ and CD8+ T cells, IgA, IgM, IgG, and viral load in both the upper and the lower respiratory tracts. Additionally, we considered age-related changes in the immune system to model severe and critical disease states. Severity state was determined by the extent of lung tissue damage, elevated levels of IL-6 and viral load, and the observed reasonable decline in concentrations of neutralizing antibodies and CTLs. To validate the model, we conducted a series of in silico experiments: infectivity and evasion mechanisms of SARS-CoV-2, innate immune response delay and hyperactivation, HIV/SARS-CoV-2 coinfection, and interferon administration during acute COVID-19 phase.

To investigate the impact of immune system aging to COVID-19 progression (see Section 3.4), we defined a set of parameters (ε) representing key age-related processes, including the number of naïve lymphocytes, their proliferation and differentiation rates, and the efficacy of innate immunity activation. Simulation results show that a more than 30% overall decrease in the immune response efficacy leads to critical COVID-19 progression, characterized by significant alveolar epithelium damage, higher viral load, and an approximately 40% increase in viral shedding duration compared to moderate progression. Additionally, we tried to incorporate the cytokine storm mechanism through IL-6 action, a hallmark of critical COVID-19 cases often requiring mechanical ventilation [160,164]. While IL-6 promotes B cell development, its elevated level inhibits proper T cell formation and suppresses the cytotoxic activity of CTLs [191]. These highlight the complex interplay between aging and immune response in the progression of severe COVID-19.

To investigate the contribution of the innate immune response to the infection, we separately examined the hyperactivation of macrophages and the delay in dendritic cell maturation and migration. These factors contribute to a worse disease outcome, significantly increasing the likelihood of a cytokine storm, as confirmed by substantially elevated IL-6 levels (>1000 pg/mL). Furthermore, a sensitivity analysis (see Figure 3) of the constructed model highlights the important role of dendritic cell migration, showing high sensitivity to delay in IDC migration to lymph nodes. This underscores the significance of innate immunity, especially in activating the adaptive immune response.

Patients with chronic diseases like HIV are at higher risk of developing respiratory illnesses such as COVID-19 or influenza, which can lead to co-infection with multiple pathogens [192]. Although modeling coinfection is challenging due to its complexity, we can simulate the co-infection by focusing on the main consequences of chronic infection. For instance, in the case of HIV, the key change is the depletion of CD4+ T cells, which severely impairs the immune system’s ability to mount a strong response. The reconstructed model predicts significantly worse disease progression in these patients. Specifically, a reduction in CD4+ T cells of more than 60% leads to a critical state of COVID-19, causing serious alveolar epithelium damage and thus requiring hospitalization and mechanical ventilation.

Currently, many approaches to COVID-19 therapy have been developed, with vaccination being the most common. Although vaccination primarily serves as a preventive measure, there is a need for effective treatment strategies to mitigate the severity of infection in patients who are already experiencing symptoms. One promising approach is interferon administration [188], which may improve the condition of critically ill patients and prevent the development of more severe progression of COVID-19. To verify the effect of interferon administration on the immune response, we focused on protocols used by healthcare providers that involve daily injections of interferon gamma for 5–6 days [189], starting immediately after symptom onset. According to the model predictions, the treatment not only reduces viral load and alveolar epithelium damage by more than 50% but also raises baseline interferon levels even after the treatment ends, leading to a milder course of COVID-19 and ensuring faster recovery.

The developed model, like most mathematical models, relies on a number of assumptions and limitations. We acknowledge that the complexity of the proposed model, which describes an immune system with multiple interacting compartments (upper airways, lungs, and lymph nodes), numerous variables (viral load, epithelial cells, macrophages, lymphocytes, and cytokines), and nonlinear interactions makes a comprehensive analytical solution currently infeasible. This is compounded by the limited availability of precise experimental data for some model parameters and the absence of exact knowledge on mechanisms for certain molecular–genetic processes. As a result, we relied on numerical methods to simulate the system’s dynamics and calibrate the model using available experimental data (viral load, antibodies, CD4+ and CD8+ T cells, and interleukin-6 levels). However, in future work, we plan to explore analytical approaches.

Furthermore, our model does not explicitly include a blood compartment, which may introduce some biases. Since most experimental data (such as antibody levels and viral load) are derived from patient blood, and key transport processes (e.g., immune cell migration) occur through the bloodstream, this omission could affect the accuracy of species dynamics. To address this, we incorporated delay differential equations to implicitly model blood transport by accounting for the time required for these processes. However, explicitly defining a blood module remains an important step for future model refinement.

Another significant limitation is the availability of experimental quantitative data, particularly for the upper airway compartment, which may lead to a low reliability of parameter values, as demonstrated by the identifiability analysis. While the primary focus of our model is the lungs, and most experimental investigations are centered on it, we pursue the goal to expand the upper airway compartment by adding new species and reactions. To validate the dynamic behavior in the upper airway, temporal data are required, which are still scarce. Additionally, although 80% of the parameters in the lung compartment are fully identifiable, further extending the model by including key cells and molecules, such as Treg cells or interleukin-10, may lead to challenges related to parameter values and their availability. Furthermore, the model does not account for gender differences, preventing an assessment of sex-specific effects on disease progression. The model also focuses solely on the acute phase of COVID-19 and does not consider immune responses associated with long COVID.

Taking into account the identified limitations, we have outlined directions for further model development. Including cytokines and T helper cells in the upper airway compartment, as well as adding an explicitly formulated blood system module, appears to be essential for a more accurate representation of the upper airways and the transport processes within the body. Subsequent areas of interest include the consideration of long COVID and enhancing the model’s stochasticity by directly incorporating the effects of gender and age differences. As a result, the constructed model can serve as a framework, as it incorporates fundamental immunological processes, encompassing both adaptive and innate immune responses, such as the dynamics of macrophages and dendritic cells, as well as processes involving T and B cells. Applying the model to study other pathogens, such as the influenza virus or a completely novel agent in case of new pandemic, required modifying the model by incorporating key missing species if necessary and adjusting specific parameters (e.g., the viral infection rate) to values derived for the selected pathogen. Although obtaining reliable data in the early stages of a new pandemic can be challenging, certain approaches, such as using preliminary experimental data or data from related pathogens, can be effective in facilitating timely model adaptation.

A distinctive feature of the model is the integration of interconnected modules for the upper airways and the lungs, enabling a more natural representation of respiratory infections by capturing the process of pathogen migration from the upper to the lower airways. The collected and calculated data for both initial conditions and parameter values are relatively extensive compared to other mathematical models (Table 6), although many parameters still had to be estimated due to the model’s complexity. Despite the relatively large number of parameters, our analysis shows that most of them are fully identifiable in the lung compartment, but only partially identifiable in the upper airways (see Section 3.2). This indicates that the available data are sufficient for reliable parameter estimation in the lungs.

To compare the developed model with major existing within-host SARS-CoV-2 models, we summarized key characteristics such as the number of parameters and types of data used for model calibration (Table 6). The number of parameters estimated via optimization, when specified in the original publication, is shown in parentheses. It should be noted that this comparison does not reflect the scientific value of the models and is intended solely to provide an overview of current models’ structure.

Taken together, these results support the validity of the described model and its suitability for various types of analysis, as well as its potential for extension or adaptation to other pathogen infections.

## Figures and Tables

**Table 1:** Comparison of the optimal solutions provided by the used optimization methods.

Method	$\chi^{2}$	P	AIC
MOPSO	5.7 × 10^9^	3 × 10^3^	26.4
SRES	7.3 × 10^9^	2 × 10^9^	33.4
MOCell	7.5 × 10^9^	1 × 10^4^	27.3
Initial model	2 × 10^10^	8.1 × 10^10^	36.3

**Table 2:** Criteria for disease severity modes.

Severity Mode	Epithelial Damage (Healthy Cells, ×10^8^)	Epithelial Damage (Healthy Cells, %)	Viral Load (RNA Copies/mL, ×10^8^)	IL-6 (pg/mL)
Moderate	>3.85	>70	<2.0	<500
Severe	2.2–3.85	40–70	2.0–4.3	500–1000
Critical	<2.2	<40	>4.3	>1000

**Table 3:** Age-related processes and associated parameters.

Parameter	Description of the Process	References
$\varepsilon_{1}$	Proliferation of naïve CD4+ T cells	[135,136,137,144]
$\varepsilon_{2}$	Proliferation of naïve CD8+ T cells	
$\varepsilon_{3}$	Naïve CD8+ T cell differentiation into CTL	
$\varepsilon_{4}$	Proliferation of naïve B cells	[132,133]
$\varepsilon_{5}$	Immature dendritic cell maturation and migration to the lymph nodes	[135]
$\varepsilon_{6}$	Virion neutralization by immunoglobulins (IgA, IgG, IgM)	[140]
$\varepsilon_{7}$	Infected epithelial cell elimination by CTLs	[139]
$\varepsilon_{8}$	Activation of resting macrophages	[143,150]

**Table 4:** Parameter values for disease severity modes.

Parameter	Severity Mode		
	Moderate (100%)	Severe (90–80%)	Critical (80–70%)
$\varepsilon_{1}-\varepsilon_{8}$	1	0.9	0.8
$B_{n}$, cell/mL	60,000	48,000	42,000
$T_{n}$, cell/mL	33,000	26,400	23,100
$H_{n}$, cell/mL	100,000	80,000	70,000
$B_{n}$(ua), cell/mL	16,000	12,800	11,200
$T_{n}$(ua), cell/mL	33,000	26,400	23,100

**Table 5:** Validation scenarios.

Scenario Name	Model Adjustments	Results				
		Viral Load	Epithelium Damage	CTLs Response	IgG Response	IL-6
Macrophage hyperactivation	$a_{{Mr}_{Ma}}\;$(4.7, 14.5) $p_{Mr}$ (1.1, 3.1)	-	Increase	Decrease	-	Increase
Dendritic cell migration delay	$t_{IDC}$ (0.8, 1.8)	Increase	Increase	Decrease	Decrease	Increase
CD4+ T cell depletion	$H_{n}$ (100%, 5%)	Increase	Increase	Decrease	Decrease	Increase
Impaired T cell development	$p_{CD4},\;p_{CD8}$ (100%, 5%)	Increase	Increase	Decrease	Decrease	-
Enhanced viral infectivity and immune evasion	$i_{V_{EP}}$ (1.1 × 10^−9^, 1.8 × 10^−9^) $e_{V_{Ig}}$ (0.3, 0.1)	Increase	Increase	Decrease	-	Increase
Interferon administration	2000 pg/mL of ${IFN}_{g}$ for 5 days after symptom onset	Decrease	Decrease	Increase	Decrease	Decrease
Inhibited viral replication	Reduce the efficiency of viral replication by 25% each day for 5 days after the onset of symptoms	Decrease	Decrease	Increase	Decrease	Decrease

The yellow background denotes a negative influence of the corresponding variable on the immune response, while the green background indicates a positive effect. A dash represents a non-significant change in the variable.

**Table 6:** Comparison of SARS-CoV-2 models.

Model	DE	Parameters	Modules	Adaptive immunity	Innate Immunity	Experimental Data and Dataset Sizes
Current model	35	112 (59)	4	+	+	V (UA: 232, L: 56), CD4/8 (12, 6), Ig (A: 677, G: 695, M: 676), IL-6: 14 B* (CD4, CD8, B)
Leander et al., 2021 [13]	14	39 (10)	1	-	+	V (UA): 11
Du at al., 2020 [19]	3	21 (21)	1	+	-	-
Wang et al., 2021 [20]	7	40 (31)	3	+	+	T: 410
Grebennikov et al., 2021 [22]	12	54	1	+	+	V (UA): 38 B* (IFN, Ig, CD8)
Zhou et al., 2023 [23]	32	181 (129)	1	+	+	B* (Cytokines)
Palsson et al., 2013 [34]	55	171 (103)	4	+	+	-

DE—differential equation, B*—boundary for the specified entities, V—viral load, CD8—CD8+ T cells, CD4—CD4+ T cells, T—T cells, Ig—Immunoglobulins, B—B cells, IL-6—interleukin 6, IFN—interferons, UA—upper airway, L—lungs.

## Data Availability

The multi-scale mathematical model of the immune response to SARS-CoV-2 infection as well as all simulation results described in the manuscript are available through the web interface of the BioUML software at the GitLab project: https://gitlab.sirius-web.org/virtual-patient/modular-immune-system. It is worth noting that the simulation results are presented via Jupyter Notebooks for the independent validation of the model simulations and further numerical analysis. Furthermore, to simplify the model simulations, we have added the widget to the notebook for different simulation scenarios (see readme in the GitLab project).

## Supplementary Materials

The following supporting information can be downloaded at: https://www.mdpi.com/article/10.3390/v17050589/s1, File S1: Mathematical Model Description, File S2: Identifiability Analysis, File S3: Model Simulations, File S4: Mathematical Model (.omex), Table S1: Model Parameters, Table S2: Initial Values, Table S3: Experimental Data, Table S4: Sensitivity Analysis.
