# Supplementary material for: A Modular Mathematical Model of the Immune Response for Investigating the Pathogenesis of Infectious Diseases
Source: Viruses. 2025 Apr 22;17(5):589. doi: 10.3390/v17050589 (PMC12115727; doi:10.3390/v17050589)
Supplement: Supplementary file 1 [file viruses-17-00589-s001.zip › viruses-3549703-supplementary/Supplementary File S1. Mathematical Model Description-tracked.pdf]

The developed model consists of four compartments: the upper airways, lungs, and lymph nodes draining each module separately. In total, it includes 35 differential equations and 112 parameters: 8 equations and 29 parameters for the lungs, 15 equations and 48 parameters for the lung lymph nodes, 6 equations and 16 parameters for the upper airways, and 6 equations and 19 parameters for the lymph nodes of the upper airways**.**

Upper Airways and Lymph Nodes

The infection process begins when the nasal and oral cavities are exposed to free viral particles (V) present in inhaled air droplets (S1 Text. Figure 1). The amount of virus sufficient to initiate an infection, known as the infectious dose, is detailed in Section 2.2. The viral infection usually spreads quickly through the upper airways, comprising the nasal and oral cavities and pharynx. Because of this rapid spread, we overlook minor differences in infection times in these areas and consider them one compartment, which we designated as the "upper airways (UA)." We considered the moment of exposure to the virus as the starting point (t = 0) for the model simulation. Inhaled SARS-CoV-2 becomes activated and disrupts the equilibrium state in which the organism was before the infection, thereby mounting the appropriate immune response.

Upon entering the body, virions infect susceptible (see Section 2.2) epithelial cells (EP) at a rate of $i_{V_{EP}}$ (Equation (1)). However, infected cells do not immediately begin releasing new virions. For SARS-CoV-2, this process typically occurs approximately 12 to 36 hours later [1]. To model this intracellular incubation period, we introduced exposed epithelial cells (EP_e), which act as intermediates between healthy and infected cells (EP_i) that are capable of producing virions. Healthy epithelial cells remain in a stationary state if there is no infection. Therefore, we defined the regeneration and decay processes as $d_{EP} * (EP_0 - [EP])$, where EP_0 represents the initial number of healthy epithelial cells and d_{EP} represents the death rate. The transition from exposed to infected cells occurs at a rate of $a_{EPe_{EPi}}$ (Equation (2)), which, together with the rate $i_{V_{EP}}$, defines the number of cells in the incubation phase. Thus, the combination of $i_{V_{EP}}$and $a_{EPe_{EPi}}$ determines the duration of incubation. Infected epithelial cells naturally decay at a rate of d_{EPi} along with the elimination by cytotoxic T cells (CTL_{ua}) at a rate of $e_{EPi_{CTL}}$ (Equation (3)).

$$\frac{d(EP)}{dt} = -i_{V_{EP}} * [\text{V}] * [EP] + d_{EP} * EP_0 - d_{EP} * [EP] \tag{1}$$

$$\frac{d(EP_e)}{dt} = i_{V_{EP}} * [\text{V}] * [EP] - a_{EPe_{EPi}} * [EP_e] \tag{2}$$

$$\frac{d(EP_i)}{dt} = a_{EPe_{EPi}} * [EP_e] - d_{EPi} * [EP_i] - e_{EPI_{CTL}} * [EP_i] * [CTL_{ua}] \tag{3}$$

As epithelial cells become infected, they begin to produce and release virions at a rate of $r_{V_{EPi}}$ (Equation (4)). These virions naturally decay at a rate of d_V. Antibodies produced during the immune response neutralize free viral particles at a rate of $e_{V_{Ig}}$. By dividing the model into two complex modules, we can simulate the migration of the virus from the upper airways through the respiratory tract to the lungs. We designated the rate of this process as $m_{V_{lungs}}$.

$$\frac{d(V)}{dt} = r_{V_{EPi}} * [EP_i] - e_{V_{Ig}} * [V] * [Ig] - d_V * [V] - m_{V_{lungs}} * [\text{V}] \tag{4}$$

The emerging virus activates the resident immature dendritic cells (IDC) at a rate of $m_{IDC_{ln}}$ (Equation (5)). This process depends on the presence of the virus, which is described by the saturation constant $s_{V_{IDC_{migr}}}$. After activation, IDCs migrate to the nearest lymph nodes, where they undergo maturation and eventually differentiate into mature dendritic cells (MDC). Inflammation resulting from the infection promotes the recruitment of new dendritic cells at a rate of p_{IDC}. We assume the extent of inflammation depends on the viral load, and thus we introduced this process with the saturation of the virus equaling $s_{V_{IDC_{recr}}}$. Similar to epithelial cells, dendritic cells remain in a stationary state in the absence of infection. Therefore, we describe this process as $d_{IDC} * (IDC_0 - [IDC])$, where IDC_0 represents the number of immature dendritic cells at the initial time, and d_{IDC} represents their death rate. Activated and differentiated dendritic cells naturally decay at a rate of d_{MDC} (Equation (6)).

$$\frac{d(IDC)}{dt} = p_{IDC} * [\text{IDC}] * \frac{[\text{V}]}{s_{V_{IDC_{recr}}} + [\text{V}]} - m_{IDC_{ln}} * [\text{IDC}] * \frac{[\text{V}]}{s_{V_{IDC_{migr}}} + [\text{V}]} + d_{IDC} * IDC_0 - d_{IDC} * [\text{IDC}] \tag{5}$$

$$\frac{d(MDC)}{dt} = m_{IDC_{ln}} * [\text{IDC}] * \frac{[\text{V}]}{s_{V_{IDC_{migr}}} + [\text{V}]} - d_{MDC} * [MDC] \tag{6}$$

In turn, MDCs stimulate naive B (B_n) and T (T_n) lymphocytes residing in the head and neck lymph nodes. Antigen presentation by dendritic cells leads to the proliferation of lymphocytes at rates of p_B and p_{CD8} for B and T cells, respectively (Equations (7), (8)). Since the process directly depends on the presence of activated dendritic cells, we used the Michaelis–Menten equation with MDC saturation constants $s_{MDC_{B_{prol}}}$ and $s_{MDC_{CD8_{prol}}}$. Furthermore, both naive T and B cells maintain an equilibrium state in the absence of infection. Therefore, we described this state similarly to epithelial and dendritic cells, with d_B and d_{CD8} corresponding to the death rates of B and T cells, respectively.

$$\frac{d(B_n)}{dt} = p_B * [B_n] * \frac{[MDC]}{s_{MDC_{B_{prol}}} + [MDC]} - a_{B_P} * [B_n] * \frac{[MDC]}{s_{MDC_{B_{diff}}} + [MDC]} + d_B * B_0 - d_B * [B_n] \quad (7)$$

$$\frac{d(T_n)}{dt} = p_{CD8} * [T_n] * \frac{[MDC]}{s_{MDC_{CD8_{prol}}} + [MDC]} - a_{CD8_{CTL}} * [T_n] * \frac{[MDC]}{s_{MDC_{CD8_{diff}}} + [MDC]} + d_{CD8} * CD8_0 - d_{CD8} * [T_n] \quad (8)$$

The evolving infection causes the activation of naive lymphocytes. Since the UA compartment is a simplified version of the lung compartment, we do not include CD4+ T cells and their subpopulations, or a pool of cytokines, implying their action indirectly. Naive T cells differentiate into cytotoxic T cells (CTL) at a maximum rate of $a_{CD8_{CTL}}$ (Equation (9)), with dendritic cell saturation of this reaction set to $s_{MDC_{CD8_{diff}}}$. Effector T cells decay at a rate of d_{CTL}. Since only about 15% of CTLs migrate to the site of infection [2], we introduced an additional entity (CTL_{ua}) to represent the population of CTLs in the upper airways and implemented a displacement time delay t_{CTL} of 0.5 day. The rate of this reaction is equal to $m_{CTL_{ua}}$ (Equation (10)).

$$\frac{d(CTL)}{dt} = a_{CD8_{CTL}} * [T_n] * \frac{[MDC]}{s_{MDC_{CD8_{diff}}} + [MDC]} - d_{CTL} * [CTL] \quad (9)$$

$$\frac{d(CTL_{ua})}{dt} = m_{CTL_{ua}} * [CTL][t - t_{CTL}] - d_{CTL} * [CTL_{ua}] \quad (10)$$

On the other hand, B cells differentiate into plasma cells (P), which are the primary producers of antibodies (Ig), at a maximum rate of a_{B_P} (Equation (11)). Dendritic cells play a critical role in the activation of B cells, with MDC saturation in the reaction denoted by $s_{MDC_{B_{diff}}}$. In the UA module, we consider antibodies in a generalized form without categorizing them into classes. Like other cells, plasma cells naturally decay at rate of d_P. Antibodies are secreted at a rate of r_{Ig} and gradually decrease over time at a rate of d_{Ig} (Equation (12)).

$$\frac{d(P)}{dt} = a_{B_P} * [B_n] * \frac{[MDC]}{s_{MDC_{B_{diff}}} + [MDC]} - d_P * [P] \tag{11}$$

$$\frac{d(Ig)}{dt} = r_{Ig} * [P] - d_{Ig} * [Ig] \tag{12}$$

Overall, the upper airways consists of 6 ODEs and 16 parameters, and the corresponding lymph nodes consist of 6 equations and 19 parameters. The dynamics of viral load in the UA was approximated to the corresponding experimental data [3], thus facilitating more precise modeling of the infection process in the lungs.

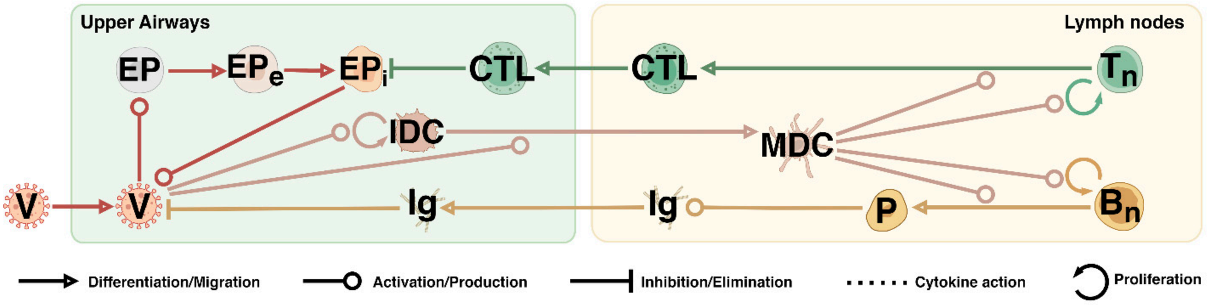

S1 Text. Figure 1. *A schematic representation of the upper airway compartment of the model. The process notations are provided below the diagram. V – virus, EP – epithelial cells (e – exposed, i – infected), DC – dendritic cells (I – immature, M - mature), Tn – naïve CD8+ T cells, Bn – naïve B cells, CTL – cytotoxic T cells, P – plasma cells, Ig – immunoglobulins.*

Lungs

As the viral load in the upper airways (UA) increases, SARS-CoV-2 gradually begins to infect the lower respiratory tract, represented by the lungs in our model (S1 Text, Figure 2). This process occurs at a rate of $m_{V_{lungs}}$ (Equation (13)), indicating both the influx of the virus to the lungs and the outflow from the UA (Equation (4)).

$$\frac{d(V)}{dt} = m_{V_{lungs}} * [V_{ua}] + r_{V_{EPi}} * [EP_i] - e_{V_{IgG}} * [V] * [IgG] - e_{V_{IgA}} * [V] * [IgA] - e_{V_{IgM}} * [V] * [IgM] - d_V * [V] \quad (13)$$

At the end of the SARS-CoV-2 incubation period, infected epithelial cells begin releasing new viral particles at a rate of $r_{V_{EPi}}$. Due to natural degradation processes, the virus decay at a rate of d_V. In addition to this, virions are neutralized by immunoglobulins of three main classes (IgG, IgA, IgM) at rates of $e_{V_{IgG}}$, $e_{V_{IgA}}$, and $e_{V_{IgM}}$, respectively. The antibodies are secreted by plasma cells and then transferred to the lungs, reflecting the humoral immune response.

In the lungs, we modeled the infection process of epithelial cells similarly to the upper airways: healthy epithelial cells (EP) are infected by SARS-CoV-2 and become exposed (EP_e). They represent the incubation period of the virus and do not release virions during their lifetime. Eventually, cells become completely infected (EP_i) and start to produce new virions. Similar to the UA, healthy alveolar cells remain in a stationary state in the absence of infection. This process is described by the term $d_{EP} * (EP_0 - [EP])$, where d_{EP} represents the death rate of epithelial cells and EP_0 denotes the initial number of epithelial cells in the lungs. Interferons are known to play a crucial role in intracellular immunity by employing various mechanisms such as blocking viral entry into host cells, inhibiting viral replication, or promoting the proteolysis of viral proteins [4–7]. We modeled the interferon action by introducing inhibition of viral entry during the infection process, with the constant $s_{IFN_{EP_{inf}}}$ representing interferon saturation (Equation (14)). Infected epithelial cells are eliminated by cytotoxic T cells, which migrate to the lungs from lymph nodes (Equation (26)) at a rate denoted as $e_{EPI_{CTL}}$. Interleukin 6 is known not only to facilitate the development of lymphocytes but also to impair the proliferation and cytotoxic activities of cytotoxic T cells. This might significantly contribute to severe COVID-19 [8]. We incorporated this IL-6 effect by modeling its inhibitory action on epithelial cell elimination, using the IL-6 saturation constant $s_{IL6_{EPi_{elim}}}$.

$$\frac{d(EP)}{dt} = -i_{V_{EP}} * [\mathrm{V}] * [EP] * \frac{s_{IFN_{EP_{inf}}}}{s_{IFN_{EP_{inf}}} + [IFN_g]} + d_{EP} * EP_0 - d_{EP} * [EP] \quad (14)$$

$$\frac{d(EP_e)}{dt} = i_{V_{EP}} * [\mathrm{V}] * [EP] * \frac{s_{IFN_{EP_{inf}}}}{s_{IFN_{EP_{inf}}} + [IFN_g]} - a_{EPe_{EPi}} * [EP_e] \tag{15}$$

$$\frac{d(EP_i)}{dt} = a_{EPe_{EPi}} * [EP_e] - d_{EPi} * [EP_i] - e_{EPI_{CTL}} * [EP_i] * [CTL_{lungs}] * \frac{s_{IL6_{EPi_{elim}}}}{s_{IL6_{EPi_{elim}}} + [IL_6]} \tag{16}$$

The presence of virions in the lungs and local inflammation stimulates the production of various chemokines (which are not included in the model), inducing recruitment of new dendritic cells to the site of infection [9–11] at a maximum rate of p_{IDC} and a half-saturation of virus particles $s_{V_{IDC_{prol}}}$ (Equation (17)). Following exposure to the virus, tissue-resident immature dendritic cells (IDC), which normally reside in the lungs alongside macrophages [9], undergo activation and subsequent maturation [12]. Throughout the process, they amplify the expression of MHC II molecules, produce costimulatory molecules on the cell surface, and reduce antigen internalization. At the same time, they migrate from the lungs to the lymph nodes [11]. In our model, these complex processes are encapsulated in the $m_{IDC_{ln}}$ constant and depend on the number of free virions in the lungs, with their half-saturation equal to $s_{V_{IDC_{migr}}}$. To account for the time required for migration and maturation, we introduced a delay function for dendritic cells and viral saturation, with a time delay set to t_{IDC}. IDCs decay at a rate of d_{IDC} and regenerate at a rate of $d_{IDC} * IDC_0$, where IDC_0 represents the initial number of immature dendritic cells in the lungs.

$$\frac{d(IDC)}{dt} = p_{IDC} * [IDC] * \frac{[V]}{s_{V_{IDC_{prol}}} + [V]} - m_{IDC_{ln}} * [IDC](t - t_{IDC}) * \frac{[V](t - t_{IDC})}{s_{V_{IDC_{migr}}} + [V](t - t_{IDC})} + d_{IDC} * IDC_0 - d_{IDC} * [IDC] \tag{17}$$

Another crucial component of innate immunity is macrophages. We introduced two types of these cells: resting and activated. Resting resident macrophages (M_r) undergo natural turnover at a constant rate of $d_{Mr} * M_0$, where M_0 represents the initial number of resting macrophages in the lungs. Their natural decay occurs at a rate of d_{Mr}, allowing resting

macrophages to remain in equilibrium throughout the absence of infection. During infection, new monocytes are recruited into the lungs and subsequently differentiate into alveolar macrophages [13]. This recruitment occurs at a rate of p_{Mr} and strongly depends on the number of activated (M_a) macrophages, which serve as the main source of chemokines that play a chemoattractant role [14]. When the virus appears in the lungs, resident resting macrophages become activated through the phagocytosis of infected and dying epithelial cells, followed by Toll-like receptor (TLR) activation by viral antigens. This process has been observed in cases of COVID-19 [15] and is implied, though not directly implemented in the model. Activation of resting macrophages occurs at a constant rate of $a_{Mr_{Ma}}$, with a half-saturation constant for free virus particles in the lungs set to $s_{V_{Mr_{act}}}$, highlighting the significant role of the virus in the activation process (Equation (19)). Additionally, the reaction is regulated by elevated levels of interferon gamma (IFN_g) induced by the infection, which primes macrophages to mount a pro-inflammatory response against intracellular pathogens [16]. It was believed that lung-resident macrophages primarily do not function as antigen-presenting cells for CD4+ T cell stimulation in lymph nodes, thus assigning the role of antigen presentation to dendritic cells [17]. However, recent studies elucidated that activated macrophages can transport antigens to lymph nodes and contribute to the adaptive immune response [18,19]. Despite these findings, we did not implement this in our model and considered dendritic cells as the only antigen-presenting cells. In our model, macrophages participate in the immune response primarily by secreting cytokines, particularly IL-12 and IL-6. Macrophage activation, in addition to being influenced by the virus, heavily depends on the presence of interferon gamma, with the saturation constant for IFN_g in this reaction set to $s_{IFN_{Mr_{act}}}$. Furthermore, in contrast to activation, M_a undergo deactivation back to the resting macrophages at a max rate of $a_{Ma_{Mr}}$, which is significantly lower than the activation level. Besides this, activated macrophages naturally decay at a corresponding rate of d_{Ma}.

$$\frac{d(M_r)}{dt} = p_{Mr} * [M_a] + a_{Ma_{Mr}} * [M_a] - a_{Mr_{Ma}} * [M_r] * \frac{[IFN_g]}{s_{IFN_{Mr_{act}}} + [IFN_g]} * \frac{[V]}{s_{V_{Mr_{act}}} + [V]} + d_{Mr} * M_0 - d_{Mr} * [M_r] \quad (18)$$

$$\frac{d(M_a)}{dt} = a_{Mr_{Ma}} * [M_r] * \frac{[IFN_g]}{s_{IFN_{Mr_{act}}} + [IFN_g]} * \frac{[V]}{s_{V_{Mr_{act}}} + [V]} - a_{Ma_{Mr}} * [M_a] - d_{Ma} * [M_a] \quad (19)$$

Thus, the lung compartment consists of 8 ODEs, including CTLs in the lungs, and 29 kinetic parameters. It represents the activation of innate immunity (macrophages and dendritic cells) in response to the virus exposure, the infection of epithelial cells, and their turnover, together with SARS-CoV-2 replication. Additionally, it encompasses the immune response involving antibodies and cytotoxic T-cells.

Lymph Nodes

Lymph nodes play a key role in the adaptive immunity, orchestrating both B and T cell responses. Their activation takes place when mature dendritic cells (MDC) interact with lymphocytes in the lymph nodes, stimulating their proliferation and differentiation. Dendritic cells migrate and mature after internalizing and processing the virus in the lungs. The equation for MDCs follows the same pattern as in Equation (17). Additionally, mature dendritic cells decay at a rate of d_{MDC}.

$$\frac{d(MDC)}{dt} = m_{IDC_{ln}} * [IDC](t - t_{IDC}) * \frac{[V](t - t_{IDC})}{s_{V_{IDC_{migr}}} + [V](t - t_{IDC})} - d_{MDC} * [MDC] \quad (20)$$

As noted earlier in Section 2.1.2, dendritic cells are considered solely as antigen-presenting cells (APCs) in the model. Consequently, their influx into lymph nodes results in the presentation of viral antigens to naïve B cells (B_n) in the cortex region of lymph nodes and to CD4+ (H_n) and CD8+ (T_n) naive T cells in the paracortex [20,21]. Given that naive T cells inherently undergo different development and express distinct receptors, we consider naive CD4+ and CD8+ T cells as separate entities in the model. In the absence of infection, both types of naïve T cells are maintained in a steady state, described by the term $d_T * (CD4_0 - [H_n])$ for H_n, where $CD4_0$ represents the initial number of CD4+ T cells and d_T is the natural death rate, which is similar for both types of T cells (Equation (21)). CD8+ naive T cells follow the same pattern. The first step in T cell development in response to infection involves antigen recognition through interactions with APCs. This process includes robust engagement of the T-cell receptor with the epitope

presented in complex with MHC molecules on mature dendritic cells, along with simultaneous binding of the CD28 molecule on T cells to its ligands, CD80 and CD86, on APCs [22,23]. These events lead to the clonal expansion of activated T cells, resulting in a 100- to 1000-fold increase in specific lymphocytes, depending on their lineage [24,25]. We incorporated all these molecular processes into the reaction of naïve T cell proliferation, which occurs at maximum rates p_{CD4} and p_{CD8} for CD4+ and CD8+ T cells. Mature dendritic cells drive this process, with their half-saturation set to $s_{MDC_{CD4_{prol}}}$ and $s_{MDC_{CD8_{prol}}}$, respectively. A notably feature of severe COVID-19 is the markedly elevated level of IL-6, which strongly correlates with disease severity [26,27]. Although the exact mechanisms are not fully understood, it is strictly found out that IL-6 may contribute to T cell depletion, potentially leading to lymphopenia [28–32]. To implement this dependency, we included the inhibitory effect of IL-6 on T cell development. This effect is significant at high interleukin-6 concentrations (>1000 pg/ml) but negligible at lower levels, thereby reflecting normal immune function during moderate progression. The half-saturation of IL-6 is the same for both CD4+ and CD8+ T cell proliferation and equals $s_{IL6_{T_{prol}}}$. To underscore the relationship between T helper cells and cytotoxic T cells, we incorporated interleukin-2 [33,34]. This cytokine is crucial for the proper development of CD8+ T cells, so we included its activation effect in the reaction of CD8+ T cell proliferation, with IL-2 half-saturation constant set to $s_{IL2_{T_{prol}}}$.

$$\frac{d(H_n)}{dt} = p_{CD4} * [H_n] * \frac{[MDC]}{s_{MDC_{CD4_{prol}}} + [MDC]} * \frac{s_{IL6_{T_{prol}}}}{s_{IL6_{T_{prol}}} + [IL_6]} - a_{CD4_{Th1}} * [H_n] * \frac{[IL_{12}]}{s_{IL12_{CD4_{diff}}} + [IL_{12}]} - a_{CD4_{Tfh}} * [H_n] * \frac{[IL_6]}{s_{IL6_{CD4_{diff}}} + [IL_6]} + d_T * CD4_0 - d_T * [H_n] \quad (21)$$

$$\frac{d(T_n)}{dt} = p_{CD8} * [T_n] * \frac{[MDC]}{s_{MDC_{CD8_{prol}}} + [MDC]} * \frac{s_{IL6_{T_{prol}}}}{s_{IL6_{T_{prol}}} + [IL_6]} * \frac{[IL_2]}{s_{IL2_{T_{prol}}} + [IL_2]} - a_{CD8_{CTL}} * [T_n] * \frac{[IL_2]}{s_{IL2_{T_{diff}}} + [IL_2]} + d_T * CD8_0 - d_T * [T_n] \quad (22)$$

Clonal expansion is followed by the differentiation of T cells into effector and memory types. Effector T cells are responsible for eliminating infected cells during the acute phase of the disease and gradually decline as the infection resolves. Memory T cells, on the other hand, are capable of mounting a rapid and robust immune response if the infection recurs [35,36]. In our model, we focus solely on the immune response during the acute phase, without considering long-term protection mechanisms provided by memory lymphocytes. Consequently, naïve CD4+ T cells differentiate only into effector subtypes: helper 1 type ($Th1$) and follicular helper (Tfh) cells, which are known to be prevalent CD4+ T cells phenotypes during COVID-19 [37]. Similarly, CD8+ T cells differentiate into cytotoxic T cells (CTL). Together, these subsets represent the two main arms of the cellular immune response during viral infection [36]. Differentiation of CD4+ T cells into effector types is strongly influenced by the cytokine environment, which is different for $Th1$ and Tfh [35]. The most important cytokine for the promotion of naïve T cells into T helper 1 type is interleukin 12 (I_{12}), which is mostly secreted by dendritic cells and macrophages after their activation [38,39]. Commitment to $Th1$ occurs at a maximum rate of $a_{CD4_{Th1}}$ with a half-saturation of IL-12 equal to $s_{IL12_{CD4_{diff}}}$. In contrast, differentiation into Tfh cells primarily relies on IL-6 as an activator of the reaction [35,40], occurring at a maximum rate of $a_{CD4_{Tfh}}$ with a half-saturation of IL-6 denoted as $s_{IL6_{CD4_{diff}}}$.

$$\frac{d(Th1)}{dt} = a_{CD4_{Th1}} * [H_n] * \frac{[IL_{12}]}{s_{IL12_{CD4_{diff}}} + [IL_{12}]} - d_{Th1} * [Th1] \tag{23}$$

$$\frac{d(Tfh)}{dt} = a_{CD4_{Tfh}} * [H_n] * \frac{[IL_6]}{s_{IL6_{CD4_{diff}}} + [IL_6]} - d_{Tfh} * [Tfh] \tag{24}$$

As mentioned earlier, IL-2 is crucial for CTL development, including differentiation. This process occurs at a maximum rate of $a_{CD8_{CTL}}$ with a half-saturation constant for IL-2 denoted as $s_{IL2_{T_{diff}}}$.

$$\frac{d(CTL)}{dt} = a_{CD8_{CTL}} * [T_n] * \frac{[IL_2]}{s_{IL2_{T_{diff}}} + [IL_2]} - d_{CTL} * [CTL] \tag{25}$$

Effector T cells naturally decay at rates of d_{Th1}, d_{Tfh}, and d_{CTL} for Th_1, T_{fh}, and CTL, respectively. This decline generally results from reduced viral concentration and the subsequent fading of the immune response. We did not explicitly model dependencies on viral load or the concentration of antigen-presenting cells, which indirectly reflect viral abundance. Instead, we assumed that the decline in T cells is obliquely regulated by activation and proliferation reactions, which heavily depend on the presence of mature dendritic cells. Once effector cytotoxic T cells are formed, they lose their ability to harbor in the lymph nodes and begin to spread throughout the body, including the lungs, the primary infection site in our model [41]. This migration is delayed in time, described by a constant t_{CTL}, and occurs at a rate of $m_{CTL_{lungs}}$, resulting in a gradual increase in CD8+ T cells in the lungs. It is important to note that CTLs in the lungs represent only about 15% of the total pool of effector cells [2]. In the lungs, effector T cells perform cytotoxic activities and eliminate infected epithelial cells, as described in Equation (16).

$$\frac{d\left(CTL_{lungs}\right)}{dt} = m_{CTL_{lungs}} * [CTL_{ln}](\mathrm{t} - t_{CTL}) - d_{CTL} * [CTL_{lungs}] \quad (26)$$

The primary role of effector CD4+ T cells, which arise from the differentiation of naive T cells, is to orchestrate the immune response through cellular interactions and cytokine production. In our model, we include cytokines IL-2, IL-6, IL-12, and IFNγ, based on COVID-19 pathogenesis research. Interleukin 6 (IL_6), a key pro-inflammatory cytokine, is primarily produced by monocytes, macrophages, and alveolar epithelial cells [42] in response to tissue damage and infection [40,43]. During COVID-19, lung epithelial cells are a major source of IL-6 [44]. The importance of IL-6 during SARS-CoV-2 infection was demonstrated by a strong positive correlation between serum levels of interleukin 6 and disease severity [26,27]. Therefore, in our model, IL-6 production is performed by infected epithelial cells and activated macrophages at rates of $r_{IL6_{EPi}}$ and $r_{IL6_{Ma}}$, respectively.

$$\frac{d(IL_6)}{dt} = r_{IL6_{EPi}} * [\mathrm{EPi}] + r_{IL6_{Ma}} * [M_a] - d_{IL6} * [IL_6] \quad (27)$$

IL-12 (IL_{12}) is another important cytokine, primarily secreted by macrophages and dendritic cells. It acts as a key stimulus for $Th1$ development from naïve T cells, thereby promoting a cellular immune response as a pro-inflammatory cytokine [45–47]. The role of IL-12

in CD4+ T cell differentiation is outlined in Equation (24). We describe its dynamics using linear equations with rate constants of $r_{IL12_{Ma}}$ and $r_{IL12_{MDC}}$ for activated macrophages and dendritic cells, respectively.

$$\frac{d(IL_{12})}{dt} = r_{IL12_{Ma}} * [M_a] + r_{IL12_{MDC}} * [MDC] - d_{IL12} * [IL_{12}] \tag{28}$$

Interferon gamma (I_{gamma}) belongs to type II interferons and is predominantly secreted by T helper 1 type cells (Th1), CD8+ T lymphocytes [48], and, to a lesser extent, natural killer (NK) and natural killer T (NKT) cells [49], macrophages, B cells, eosinophils, and neutrophils [50]. In the model, we implemented the secretion of interferon gamma solely by $Th1$ and CTL, given their major role in cytokine production. The rates of these processes are denoted by $r_{IFN_{Th1}}$ and $r_{IFN_{CTL}}$, respectively.

$$\frac{d(IFN_g)}{dt} = r_{IFN_{Th1}} * [\mathrm{Th1}] * \frac{[V]}{s_{V_{IFN_{prod}}} + [V]} + r_{IFN_{CTL}} * [CTL] * \frac{[V]}{s_{V_{IFN_{prod}}} + [V]} - d_{IFN} * [IFN_g] \tag{29}$$

Furthermore, we included reactions for the production and degradation of IL-2 (IL_2), which is crucial for CD8+ T cell development during the immune response [33,34]. IL_2 is primarily produced by effector CD4+ T cells [51], highlighting the dependence of CTLs on helper cells. Secretion of IL-2 occurs at rates of $r_{IL2_{Th1}}$ and $r_{IL2_{Tfh}}$ for T helper 1 type and T follicular helper cells.

$$\frac{d(IL_2)}{dt} = r_{IL2_{Th1}} * [Th1] + r_{IL2_{Tfh}} * [\mathrm{Tfh}] - d_{IL2} * [IL_2] \tag{30}$$

In addition, each cytokine undergoes natural decay at rates of d_{IL6}, d_{IL12}, d_{IL2}, and d_{IFN} for IL_6, IL_{12}, IL_2, and IFN_g, respectively.

It is known that B cells can be activated through two primary pathways. One involves soluble antigens [52] that enter and circulate through the lymph, where they can either directly interact with B cells or localize at their primary sites: the spleen, lymph nodes, and tonsils [53]. The other pathway involves direct interaction with antigen-presenting cells [54]. In our model,

we focused on the latter pathway, which is carried out by mature dendritic cells. This APC-dependent mechanism is more significant and effective compared to antigen stimulation alone [55,56]. During interaction with antigen-presenting cells, B cells form an immunological synapse, enabling them to acquire bound antigens. This process is followed by the enzymatic activity of B cell lysosomes on the bonds between the APC and antigen, resulting in its uptake and processing by B cells. Additionally, if the affinity between the B cell receptor (BCR) and the antigen is sufficiently high, the BCR can mechanically pull the antigen from the APC and invaginate it into the B cell [25,57,58]. Antigen-presenting cells provide the initial signal necessary for B cell activation, but a second signal is required through interaction with T helper cells, especially T follicular helper cells, which are crucial for B cell activation [59,60]. Initially, activated B cells proliferate and then differentiate into short-lived plasma cells (SLPCs) at extrafollicular sites within the lymph node, giving rise to early-induced antibodies [61–63]. As germinal centers form within the lymph nodes follicles, activated B cells undergo proliferation and differentiate into long-lived plasma cells (LLPCs), which reside in the bone marrow and contribute to long-lasting immunity [64,65]. Given our focus on the acute phase of the immune response, which typically lasts for weeks, we considered only short-lived plasma cells in our model. T follicular helper cells, a subtype of CD4+ T cells, significantly regulate B cell development. We incorporated their influence explicitly and implicitly (through cytokines) in the processes of naive B cell proliferation and plasma cell formation.

To implement the outlined biological features, we introduced two types of B cells: naïve B cells (B_n), which reside in the lymph nodes and do not secrete antibodies, and short-lived (P) plasma cells, which are responsible for antibody production but incapable of proliferation. In the absence of infection, naive B cells maintain a steady state, characterized by their constant influx into the lymph nodes, given by $d_B * B_0$, where B_0 denotes the initial number of naive B cells. B_n also naturally decay at a rate of d_B. The activation and subsequent proliferation of B cells are described by a single equation, with the maximum rate of a reaction denoted as p_B. This rate depends on the activating effects of both mature dendritic cells (through antigen presentation) and T follicular helper cells (through direct interaction with B cells). The concentration of MDC at

which the reaction rate is half of the maximum is represented by $s_{MDC_{B_{prol}}}$, while the half-saturation of Tfh in the reaction is equal to s_{Tfh_B}.

$$\frac{d(B_n)}{dt} = p_B * [B_n] * \frac{[MDC]}{s_{MDC_{B_{prol}}} + [MDC]} * \frac{[Tfh]}{s_{Tfh_B} + [Tfh]} - a_{B_P} * [B_n] * \frac{[Tfh]}{s_{Tfh_B} + [Tfh]} + d_B * B_0 - d_B * [B_n] \quad (31)$$

The differentiation of naive B cells into plasma cells is regulated by the presence of T follicular helper cells and occurs at a rate of a_{B_P}, with the half-saturation constant for Tfh set to s_{Tfh_B}. In addition to their formation, plasma cells also undergo natural decay at a rate of d_P.

$$\frac{d(P)}{dt} = a_{B_P} * [B_n] * \frac{[Tfh]}{s_{Tfh_B} + [Tfh]} - d_P * [P] \quad (32)$$

Plasma cells are terminally differentiated B cells responsible for antibody production. In our model, we implemented short-lived plasma cells that produce antibodies of three main classes (IgM, IgA, IgG) during the acute phase of the primary immune response [25,66]. The production of antibodies is described by linear equations with rate constants r_{IgM_P}, r_{IgG_P}, and r_{IgA_P} for IgM, IgG, and IgA, respectively. Additionally, antibodies undergo natural degradation at rates of d_{IgM} for IgM, d_{IgG} for IgG, and d_{IgA} for IgA.

$$\frac{d(IgM)}{dt} = r_{IgM_P} * [\mathrm{P}] - d_{IgM} * [IgM] \quad (33)$$

$$\frac{d(IgA)}{dt} = r_{IgA_P} * [\mathrm{P}] - d_{IgA} * [IgA] \quad (34)$$

$$\frac{d(IgG)}{dt} = r_{IgG_P} * [\mathrm{P}] - d_{IgG} * [IgG] \quad (35)$$

Functionally active antibodies eliminate free virions in the lung compartments, as described in Equation (13), thereby reflecting the humoral arm of the immune response.

Overall, the lymph node compartment consists of 15 ODEs and 48 kinetic parameters. It considers the final stages of innate immunity activation, encompassing the formation of mature dendritic cells along with the initiation of the cytokine response. This leads to the activation of both cellular and humoral components of adaptive immunity, including the specialization of T

and B cells, as well as the antibody production. Consequently, these events result in the mounting of a robust immune response that eliminates the pathogen.

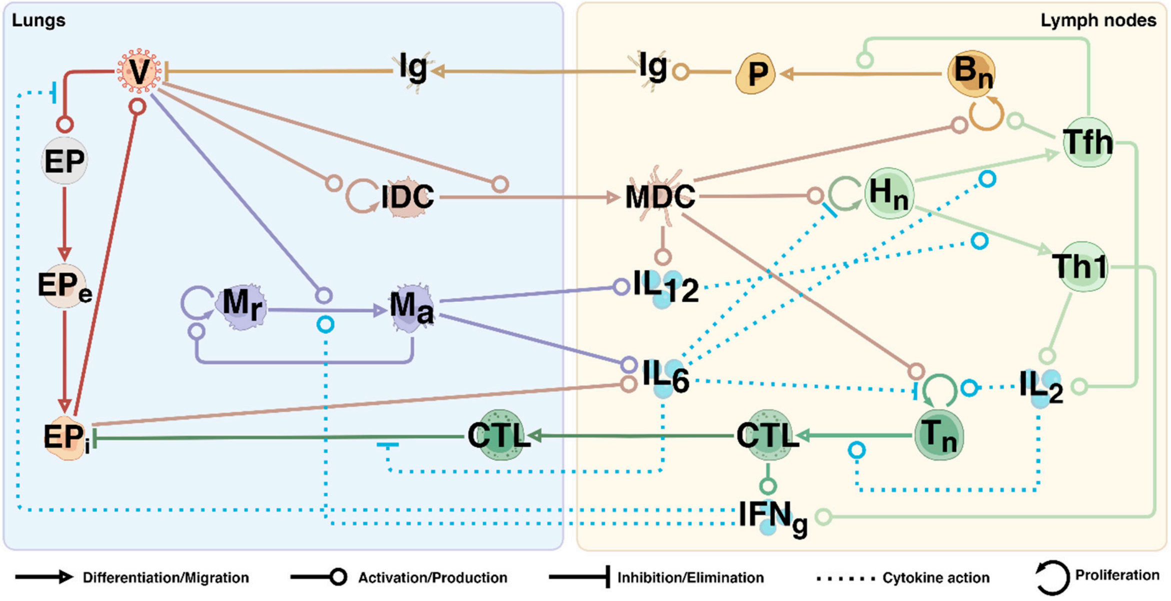

S1 Text. Figure 2. *A schematic representation of the lung compartment of the model. The process notations are provided below the diagram. V – virus, EP – epithelial cells (e – exposed, i – infected), DC – dendritic cells (I – immature, M - mature), M – macrophages (r – resting, a – activated), Hn – naïve CD4+ T cells, Tn – naïve CD8+ T cells, Bn – naïve B cells, Th1 – T helper 1 type cells, Tfh – T follicular helper cells, CTL – cytotoxic T cells, P – plasma cells, Ig – immunoglobulins (IgA, IgM, IgG), IL – interleukins (2, 6, 12), IFNg – interferon gamma.*

References

1. Bar-On YM, Flamholz A, Phillips R, Milo R. SARS-CoV-2 (COVID-19) by the numbers. eLife. 2020 Apr 2;9:e57309.

2. Lee HY, Topham DJ, Park SY, Hollenbaugh J, Treanor J, Mosmann TR, et al. Simulation and Prediction of the Adaptive Immune Response to Influenza A Virus Infection. J Virol. 2009 Jul 15;83(14):7151–65.

3. Killingley B, Mann AJ, Kalinova M, Boyers A, Goonawardane N, Zhou J, et al. Safety, tolerability and viral kinetics during SARS-CoV-2 human challenge in young adults. Nat Med. 2022 May;28(5):1031–41.

4. Hidalgo LG, Urmson J, Halloran PF. IFN-γ Decreases CTL Generation by Limiting IL-2 Production: A Feedback Loop Controlling Effector Cell Production. American Journal of Transplantation. 2005 Apr;5(4):651–61.

5. Kang S, Brown HM, Hwang S. Direct Antiviral Mechanisms of Interferon-Gamma. Immune Netw. 2018;18(5):e33.

6. Mihaescu G, Chifiriuc MC, Filip R, Bleotu C, Ditu LM, Constantin M, et al. Role of interferons in the antiviral battle: from virus-host crosstalk to prophylactic and therapeutic potential in SARS-CoV-2 infection. Front Immunol. 2024 Jan 15;14:1273604.

7. Tewari K, Nakayama Y, Suresh M. Role of Direct Effects of IFN-γ on T Cells in the Regulation of CD8 T Cell Homeostasis. The Journal of Immunology. 2007 Aug 15;179(4):2115–25.

8. Elahi R, Karami P, Heidary AH, Esmaeilzadeh A. An updated overview of recent advances, challenges, and clinical considerations of IL-6 signaling blockade in severe coronavirus disease 2019 (COVID-19). International Immunopharmacology. 2022 Apr;105:108536.

9. Chen K, Wang JM, Yuan R, Yi X, Li L, Gong W, et al. Tissue-resident dendritic cells and diseases involving dendritic cell malfunction. International Immunopharmacology. 2016 May;34:1–15.

10. Feng M, Zhou S, Yu Y, Su Q, Li X, Lin W. Regulation of the Migration of Distinct Dendritic Cell Subsets. Front Cell Dev Biol. 2021 Feb 19;9:635221.

11. Xiao Q, Xia Y. Insights into dendritic cell maturation during infection with application of advanced imaging techniques. Front Cell Infect Microbiol. 2023 Mar 2;13:1140765.

12. Al-Ashmawy GMZ. Dendritic Cell Subsets, Maturation and Function. In: Chapoval SP, editor. Dendritic Cells [Internet]. InTech; 2018 [cited 2023 Nov 9]. Available from: http://www.intechopen.com/books/dendritic-cells/dendritic-cell-subsets-maturation-and-function

13. Arish M, Sun J. Monocyte and macrophage function in respiratory viral infections. Animal Diseases. 2023 Sep 14;3(1):30.

14. Arango Duque G, Descoteaux A. Macrophage Cytokines: Involvement in Immunity and Infectious Diseases. Front Immunol [Internet]. 2014 Oct 7 [cited 2023 Nov 9];5. Available from: http://journal.frontiersin.org/article/10.3389/fimmu.2014.00491/abstract

15. García-Nicolás O, Godel A, Zimmer G, Summerfield A. Macrophage phagocytosis of SARS-CoV-2-infected cells mediates potent plasmacytoid dendritic cell activation. Cell Mol Immunol. 2023 May 30;20(7):835–49.

16. Martinez FO. Regulators of macrophage activation. Eur J Immunol. 2011 Jun;41(6):1531–4.

17. Itano AA, Jenkins MK. Antigen presentation to naive CD4 T cells in the lymph node. Nat Immunol. 2003 Aug;4(8):733–9.

18. Bissonnette EY, Lauzon-Joset JF, Debley JS, Ziegler SF. Cross-Talk Between Alveolar Macrophages and Lung Epithelial Cells is Essential to Maintain Lung Homeostasis. Front Immunol. 2020 Oct 15;11:583042.

19. Kirby AC, Coles MC, Kaye PM. Alveolar Macrophages Transport Pathogens to Lung Draining Lymph Nodes. The Journal of Immunology. 2009 Aug 1;183(3):1983–9.

20. Hughes CE, Benson RA, Bedaj M, Maffia P. Antigen-Presenting Cells and Antigen Presentation in Tertiary Lymphoid Organs. Front Immunol [Internet]. 2016 Nov 7 [cited 2023 Nov 10];7. Available from: http://journal.frontiersin.org/article/10.3389/fimmu.2016.00481/full

21. Willard-Mack CL. Normal Structure, Function, and Histology of Lymph Nodes. Toxicol Pathol. 2006 Aug;34(5):409–24.

22. Pennock ND, White JT, Cross EW, Cheney EE, Tamburini BA, Kedl RM. T cell responses: naïve to memory and everything in between. Advances in Physiology Education. 2013 Dec;37(4):273–83.

23. Punt J. Adaptive Immunity. In: Cancer Immunotherapy [Internet]. Elsevier; 2013 [cited 2024 Jul 21]. p. 41–53. Available from: https://linkinghub.elsevier.com/retrieve/pii/B978012394296800004X

24. Miller RA, Stutman O. T cell repopulation from functionally restricted splenic progenitors: 10,000-fold expansion documented by using limiting dilution analyses. The Journal of Immunology. 1984 Dec 1;133(6):2925–32.

25. Punt J, Stranford SA, Jones PP, Owen JA. Kuby immunology. Eighth edition. New York: Macmillan Learning; 2019. 1 p.

26. Chen X, Zhao B, Qu Y, Chen Y, Xiong J, Feng Y, et al. Detectable serum SARS-CoV-2 viral load (RNAaemia) is closely associated with drastically elevated interleukin 6 (IL-6) level in critically ill COVID-19 patients [Internet]. Infectious Diseases (except HIV/AIDS); 2020 Mar [cited 2023 Nov 15]. Available from: http://medrxiv.org/lookup/doi/10.1101/2020.02.29.20029520

27. Herold T, Jurinovic V, Arnreich C, Lipworth BJ, Hellmuth JC, Von Bergwelt-Baildon M, et al. Elevated levels of IL-6 and CRP predict the need for mechanical ventilation in COVID-19. Journal of Allergy and Clinical Immunology. 2020 Jul;146(1):128-136.e4.

28. Alahdal M, Elkord E. Exhaustion and over-activation of immune cells in COVID-19: Challenges and therapeutic opportunities. Clinical Immunology. 2022 Dec;245:109177.

29. Fathi N, Rezaei N. Lymphopenia in COVID-19: Therapeutic opportunities. Cell Biology International. 2020 Sep;44(9):1792–7.

30. Jafarzadeh A, Jafarzadeh S, Nozari P, Mokhtari P, Nemati M. Lymphopenia an important immunological abnormality in patients with COVID-19: Possible mechanisms. Scand J Immunol. 2021 Feb;93(2):e12967.

31. Korn T, Hiltensperger M. Role of IL-6 in the commitment of T cell subsets. Cytokine. 2021 Oct;146:155654.

32. Zhou X, Ye G, Lv Y, Guo Y, Pan X, Li Y, et al. IL-6 drives T cell death to participate in lymphopenia in COVID-19. International Immunopharmacology. 2022 Oct;111:109132.

33. Boyman O, Sprent J. The role of interleukin-2 during homeostasis and activation of the immune system. Nat Rev Immunol. 2012 Mar;12(3):180–90.

34. Hashimoto M, Im SJ, Araki K, Ahmed R. Cytokine-Mediated Regulation of CD8 T-Cell Responses During Acute and Chronic Viral Infection. Cold Spring Harb Perspect Biol. 2019 Jan;11(1):a028464.

35. Luckheeram RV, Zhou R, Verma AD, Xia B. CD4+T Cells: Differentiation and Functions. Clinical and Developmental Immunology. 2012;2012:1–12.

36. Santana MA, Esquivel-Guadarrama F. Cell Biology of T Cell Activation and Differentiation. In: International Review of Cytology [Internet]. Elsevier; 2006 [cited 2023 Nov 13]. p. 217–74. Available from: https://linkinghub.elsevier.com/retrieve/pii/S0074769606500063

37. Sekine T, Perez-Potti A, Rivera-Ballesteros O, Strålin K, Gorin JB, Olsson A, et al. Robust T Cell Immunity in Convalescent Individuals with Asymptomatic or Mild COVID-19. Cell. 2020 Oct;183(1):158-168.e14.

38. Iwasaki A, Medzhitov R. Toll-like receptor control of the adaptive immune responses. Nat Immunol. 2004 Oct;5(10):987–95.

39. Steinman RM, Hawiger D, Nussenzweig MC. Tolerogenic Dendritic Cells. Annu Rev Immunol. 2003 Apr;21(1):685–711.

40. Tanaka T, Narazaki M, Kishimoto T. IL-6 in Inflammation, Immunity, and Disease. Cold Spring Harbor Perspectives in Biology. 2014 Oct 1;6(10):a016295–a016295.

41. Weninger W, Manjunath N, Von Andrian UH. Migration and differentiation of CD8+ T cells. Immunological Reviews. 2002 Aug;186(1):221–33.

42. Crestani B, Cornillet P, Dehoux M, Rolland C, Guenounou M, Aubier M. Alveolar type II epithelial cells produce interleukin-6 in vitro and in vivo. Regulation by alveolar macrophage secretory products. J Clin Invest. 1994 Aug 1;94(2):731–40.

43. Jones SA, Jenkins BJ. Recent insights into targeting the IL-6 cytokine family in inflammatory diseases and cancer. Nat Rev Immunol. 2018 Dec;18(12):773–89.

44. Xiong Y, Liu Y, Cao L, Wang D, Guo M, Jiang A, et al. Transcriptomic characteristics of bronchoalveolar lavage fluid and peripheral blood mononuclear cells in COVID-19 patients. Emerging Microbes & Infections. 2020 Jan 1;9(1):761–70.

45. Brunda MJ. Interleukin-12. Journal of Leukocyte Biology. 1994 Feb 1;55(2):280–8.

46. Heufler C, Koch F, Stanzl U, Topar G, Wysocka M, Trinchieri G, et al. Interleukin-12 is produced by dendritic cells and mediates T helper 1 development as well as interferon-γ production by T helper 1 cells. Eur J Immunol. 1996 Apr;26(3):659–68.

47. Ma X, Yan W, Zheng H, Du Q, Zhang L, Ban Y, et al. Regulation of IL-10 and IL-12 production and function in macrophages and dendritic cells. F1000Res. 2015 Dec 17;4:1465.

48. Farrar MA, Schreiber RD. The Molecular Cell Biology of Interferon-gamma and its Receptor. Annu Rev Immunol. 1993 Apr;11(1):571–611.

49. Jorgovanovic D, Song M, Wang L, Zhang Y. Roles of IFN-γ in tumor progression and regression: a review. Biomark Res. 2020 Dec;8(1):49.

50. Ethuin F, Gérard B, Benna JE, Boutten A, Gougereot-Pocidalo MA, Jacob L, et al. Human neutrophils produce interferon gamma upon stimulation by interleukin-12. Laboratory Investigation. 2004 Oct;84(10):1363–71.

51. Bachmann MF, Oxenius A. Interleukin 2: from immunostimulation to immunoregulation and back again. EMBO Reports. 2007 Dec;8(12):1142–8.

52. Huang NN, Han SB, Hwang IY, Kehrl JH. B Cells Productively Engage Soluble Antigen-Pulsed Dendritic Cells: Visualization of Live-Cell Dynamics of B Cell-Dendritic Cell Interactions. The Journal of Immunology. 2005 Dec 1;175(11):7125–34.

53. Pieper K, Grimbacher B, Eibel H. B-cell biology and development. Journal of Allergy and Clinical Immunology. 2013 Apr;131(4):959–71.

54. Wykes M, Pombo A, Jenkins C, MacPherson GG. Dendritic Cells Interact Directly with Naive B Lymphocytes to Transfer Antigen and Initiate Class Switching in a Primary T-Dependent Response. The Journal of Immunology. 1998 Aug 1;161(3):1313–9.

55. Heath WR, Kato Y, Steiner TM, Caminschi I. Antigen presentation by dendritic cells for B cell activation. Current Opinion in Immunology. 2019 Jun;58:44–52.

56. Heesters BA, Van Der Poel CE, Das A, Carroll MC. Antigen Presentation to B Cells. Trends in Immunology. 2016 Dec;37(12):844–54.

57. Natkanski E, Lee WY, Mistry B, Casal A, Molloy JE, Tolar P. B Cells Use Mechanical Energy to Discriminate Antigen Affinities. Science. 2013 Jun 28;340(6140):1587–90.

58. Yuseff MI, Pierobon P, Reversat A, Lennon-Duménil AM. How B cells capture, process and present antigens: a crucial role for cell polarity. Nat Rev Immunol. 2013 Jul;13(7):475–86.

59. Harwood NE, Batista FD. Early Events in B Cell Activation. Annu Rev Immunol. 2010 Mar 1;28(1):185–210.

60. Ma CS, Deenick EK, Batten M, Tangye SG. The origins, function, and regulation of T follicular helper cells. Journal of Experimental Medicine. 2012 Jul 2;209(7):1241–53.

61. Elsner RA, Shlomchik MJ. Germinal Center and Extrafollicular B Cell Responses in Vaccination, Immunity, and Autoimmunity. Immunity. 2020 Dec;53(6):1136–50.

62. Nutt SL, Hodgkin PD, Tarlinton DM, Corcoran LM. The generation of antibody-secreting plasma cells. Nat Rev Immunol. 2015 Mar;15(3):160–71.

63. Oracki SA, Walker JA, Hibbs ML, Corcoran LM, Tarlinton DM. Plasma cell development and survival. Immunological Reviews. 2010 Sep;237(1):140–59.

64. Minges Wols HA. Plasma Cells. In: Encyclopedia of Life Sciences [Internet]. 1st ed. Wiley; 2006 [cited 2024 Jul 21]. Available from: https://onlinelibrary.wiley.com/doi/10.1038/npg.els.0004030

65. Nguyen DC, Lamothe PA, Woodruff MC, Saini AS, Faliti CE, Sanz I, et al. COVID -19 and plasma cells: Is there long-lived protection?*. Immunological Reviews. 2022 Aug;309(1):40–63.

66. Ionescu L, Urschel S. Memory B Cells and Long-lived Plasma Cells. Transplantation. 2019 May;103(5):890–8.
