# Supplementary material for: A Modular Mathematical Model of the Immune Response for Investigating the Pathogenesis of Infectious Diseases
Source: Viruses. 2025 Apr 22;17(5):589. doi: 10.3390/v17050589 (PMC12115727; doi:10.3390/v17050589)
Supplement: Supplementary file 1 [file viruses-17-00589-s001.zip › viruses-3549703-supplementary/Supplementary File S2. Identifiability Analysis-tracked.pdf]

S2 Appendix. Parameter Identifiability Analysis

The model simulation with the baseline parameter value and its corresponding objective function is represented by a red dot. Grey dots represent model simulations with varying parameter values and their estimated objective functions. The threshold is indicated by a dashed red line.

Out of the 59 analyzed parameters, 37 were fully identifiable, while 8 and 13 were left- and right-identifiable, respectively. Since the identifiability is relevant only for unknown parameters, we considered exclusively those estimated through model optimization (59 of 112 parameters). Notably, the analysis results for $e_{V_{IgA}}$, $e_{V_{IgM}}$, and $e_{V_{IgG}}$ were consolidated into a single variable $e_{V_{Ig}}$, as they yielded identical values.

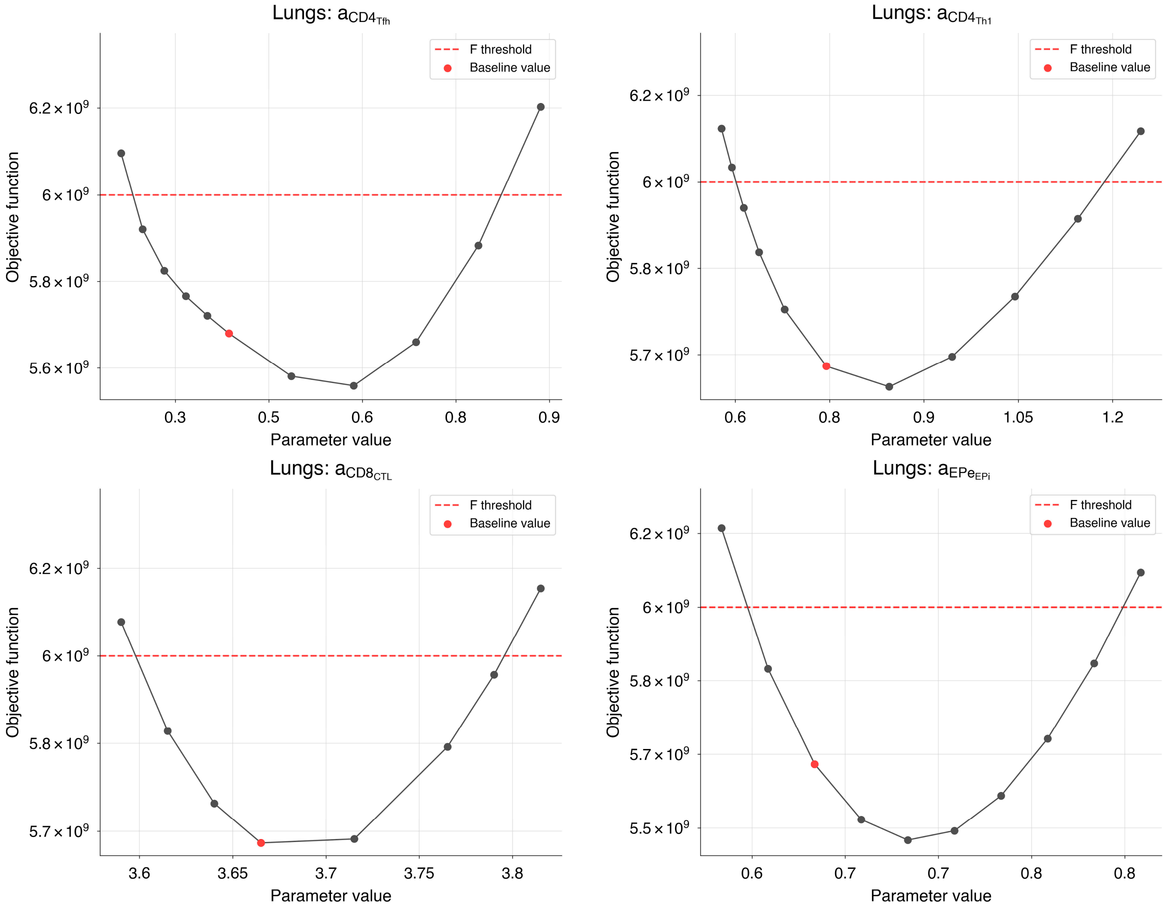

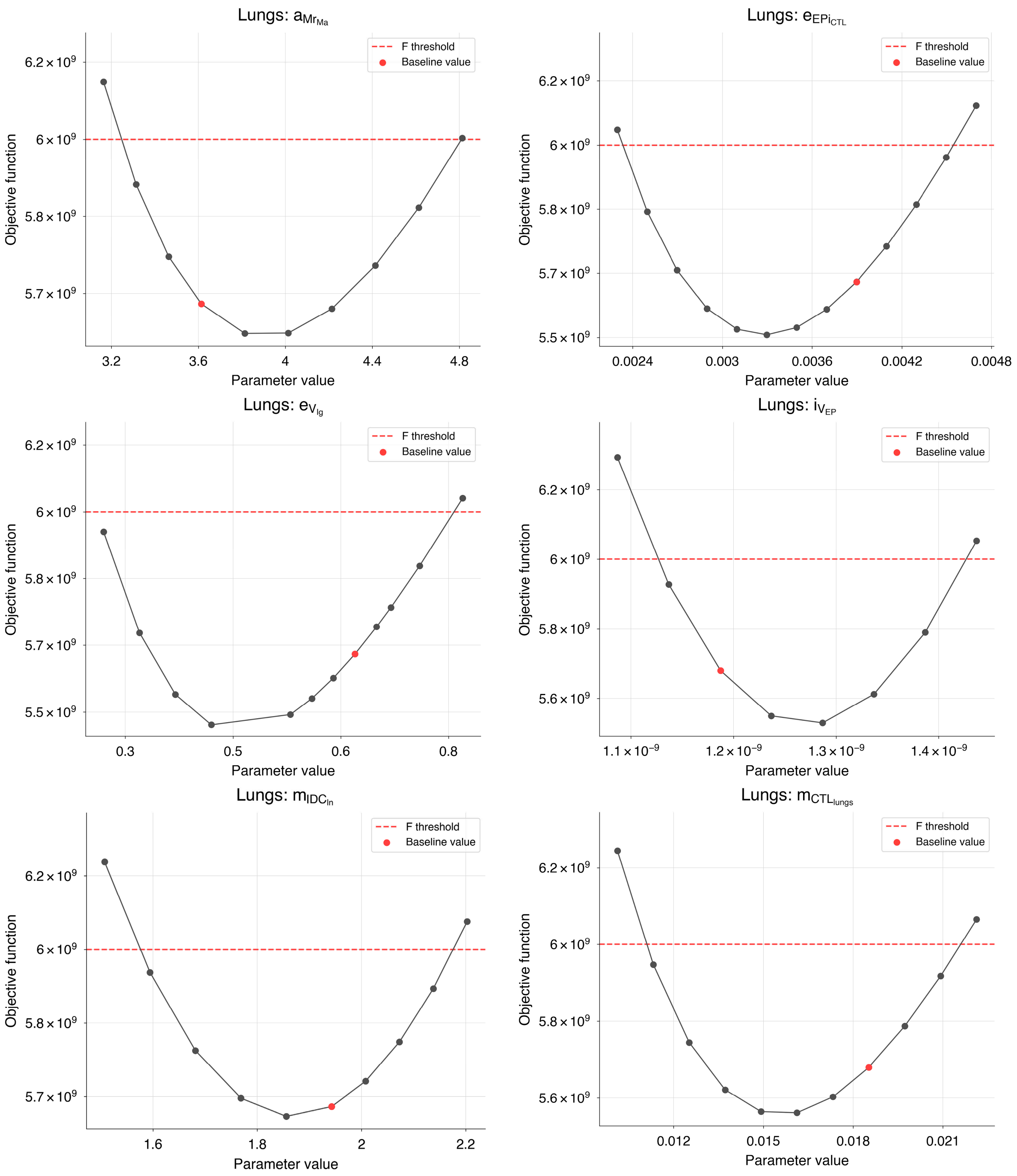
Lungs: $a_{Mr_{Ma}}$
F threshold
Baseline value
6.2×10^9
6×10^9
5.8×10^9
5.7×10^9
Objective function
3.2
3.6
4
4.4
4.8
Parameter value
Lungs: $e_{EPi_{CTL}}$
F threshold
Baseline value
6.2×10^9
6×10^9
5.8×10^9
5.7×10^9
5.5×10^9
Objective function
0.0024
0.003
0.0036
0.0042
0.0048
Parameter value
Lungs: $e_{V_{lg}}$
F threshold
Baseline value
6.2×10^9
6×10^9
5.8×10^9
5.7×10^9
5.5×10^9
Objective function
0.3
0.5
0.6
0.8
Parameter value
Lungs: $i_{V_{EP}}$
F threshold
Baseline value
6.2×10^9
6×10^9
5.8×10^9
5.6×10^9
Objective function
1.1×10^{-9}
1.2×10^{-9}
1.3×10^{-9}
1.4×10^{-9}
Parameter value
Lungs: $m_{IDC_{ln}}$
F threshold
Baseline value
6.2×10^9
6×10^9
5.8×10^9
5.7×10^9
Objective function
1.6
1.8
2
2.2
Parameter value
Lungs: $m_{CTL_{lungs}}$
F threshold
Baseline value
6.2×10^9
6×10^9
5.8×10^9
5.6×10^9
Objective function
0.012
0.015
0.018
0.021
Parameter value

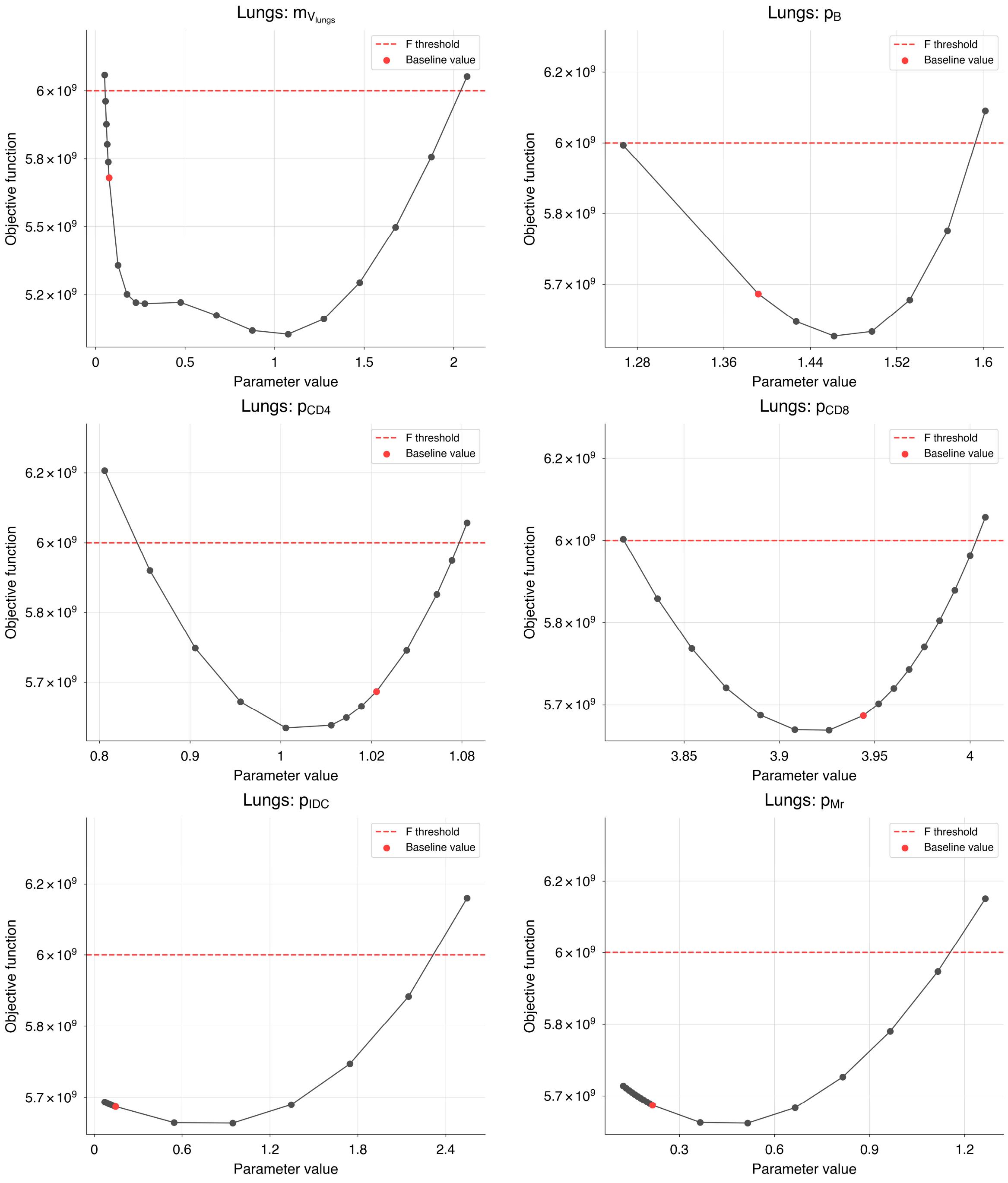

Lungs: $m_{V_{lungs}}$
Lungs: p_B
Lungs: p_{CD4}
Lungs: p_{CD8}
Lungs: p_{IDC}
Lungs: p_{Mr}
F threshold
Baseline value
Objective function
Parameter value
5.2×10^9
5.5×10^9
5.7×10^9
5.8×10^9
6×10^9
6.2×10^9
0
0.5
1
1.5
2
1.28
1.36
1.44
1.52
1.6
0.8
0.9
1
1.02
1.08
3.85
3.9
3.95
4
0
0.6
1.2
1.8
2.4
0.3
0.6
0.9
1.2

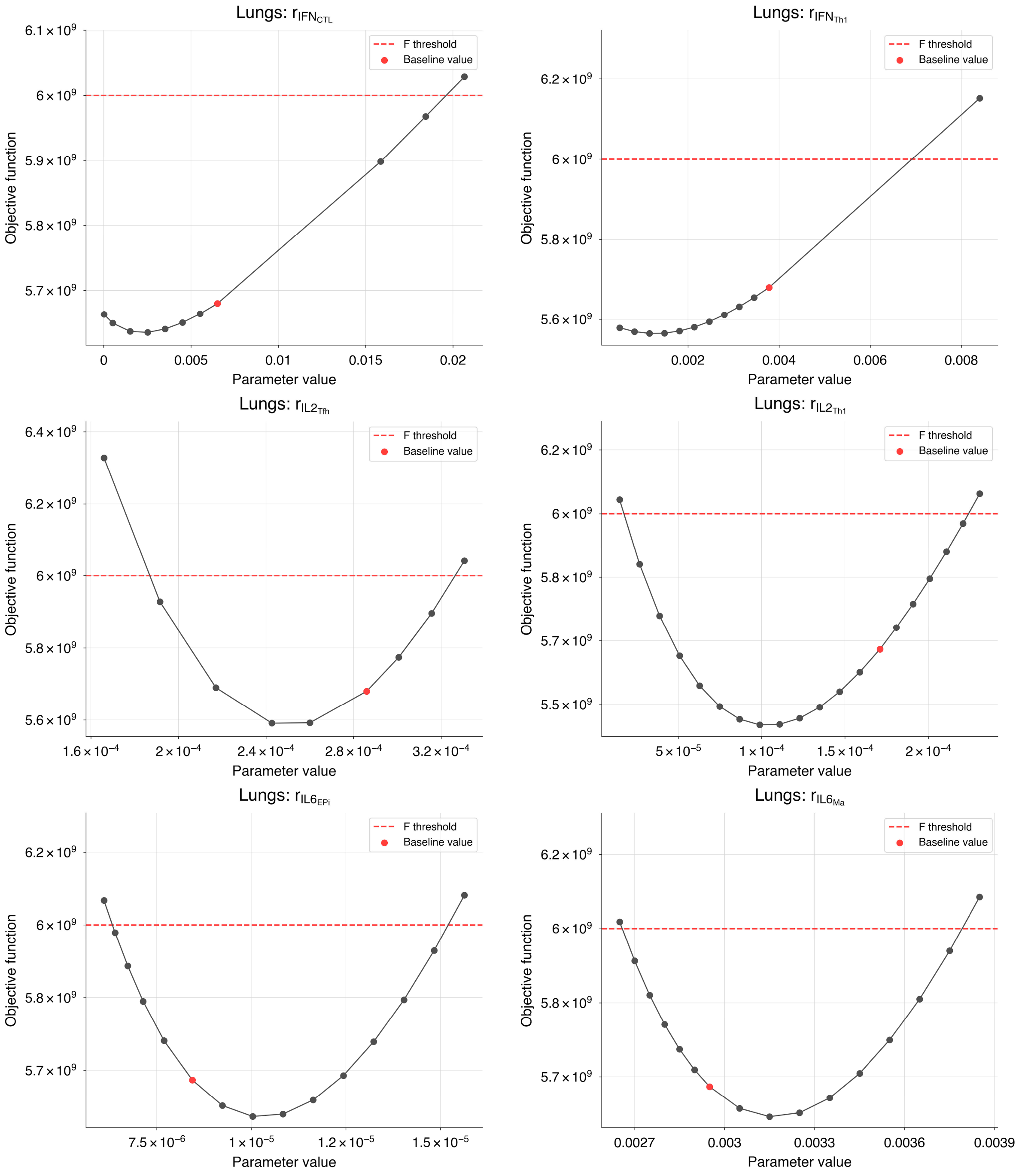

Lungs: $r_{IFN_{CTL}}$
Lungs: $r_{IFN_{Th1}}$
Lungs: $r_{IL2_{Tfh}}$
Lungs: $r_{IL2_{Th1}}$
Lungs: $r_{IL6_{EPi}}$
Lungs: $r_{IL6_{Ma}}$
F threshold
Baseline value
Objective function
Parameter value
6.1×10^9
6×10^9
5.9×10^9
5.8×10^9
5.7×10^9
6.2×10^9
5.6×10^9
6.4×10^9
5.5×10^9
0
0.005
0.01
0.015
0.02
0.002
0.004
0.006
0.008
1.6×10^{-4}
2×10^{-4}
2.4×10^{-4}
2.8×10^{-4}
3.2×10^{-4}
5×10^{-5}
1×10^{-4}
1.5×10^{-4}
7.5×10^{-6}
1×10^{-5}
1.2×10^{-5}
1.5×10^{-5}
0.0027
0.003
0.0033
0.0036
0.0039

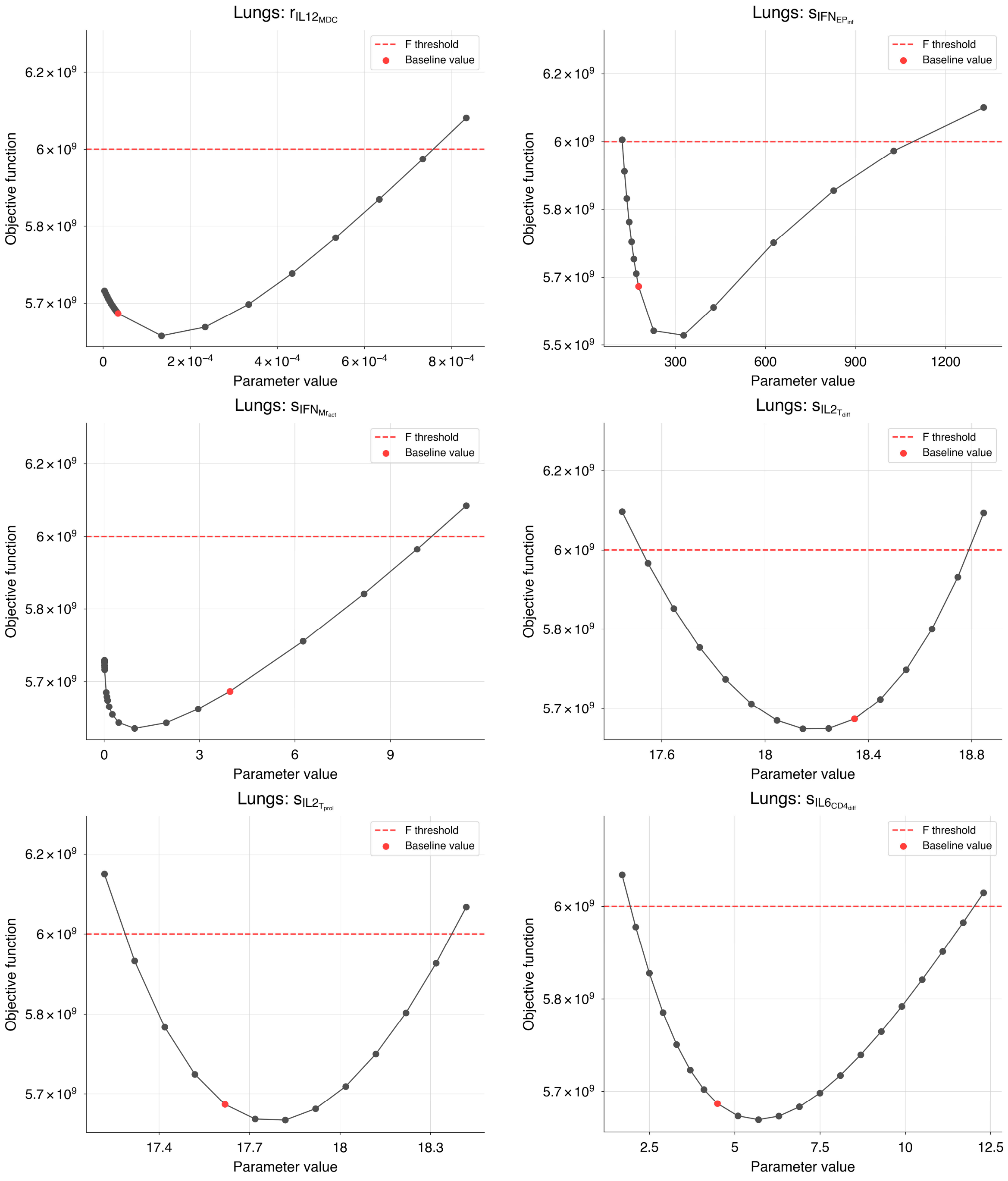

Lungs: $r_{IL12_{MDC}}$
F threshold
Baseline value
Objective function
6.2×10^9
6×10^9
5.8×10^9
5.7×10^9
0
2×10^{-4}
4×10^{-4}
6×10^{-4}
8×10^{-4}
Parameter value
Lungs: $s_{IFN_{EP_{inf}}}$
F threshold
Baseline value
Objective function
6.2×10^9
6×10^9
5.8×10^9
5.7×10^9
5.5×10^9
300
600
900
1200
Parameter value
Lungs: $s_{IFN_{M_{ract}}}$
F threshold
Baseline value
Objective function
6.2×10^9
6×10^9
5.8×10^9
5.7×10^9
0
3
6
9
Parameter value
Lungs: $s_{IL2_{T_{diff}}}$
F threshold
Baseline value
Objective function
6.2×10^9
6×10^9
5.8×10^9
5.7×10^9
17.6
18
18.4
18.8
Parameter value
Lungs: $s_{IL2_{T_{prol}}}$
F threshold
Baseline value
Objective function
6.2×10^9
6×10^9
5.8×10^9
5.7×10^9
17.4
17.7
18
18.3
Parameter value
Lungs: $s_{IL6_{CD4_{diff}}}$
F threshold
Baseline value
Objective function
6×10^9
5.8×10^9
5.7×10^9
2.5
5
7.5
10
12.5
Parameter value

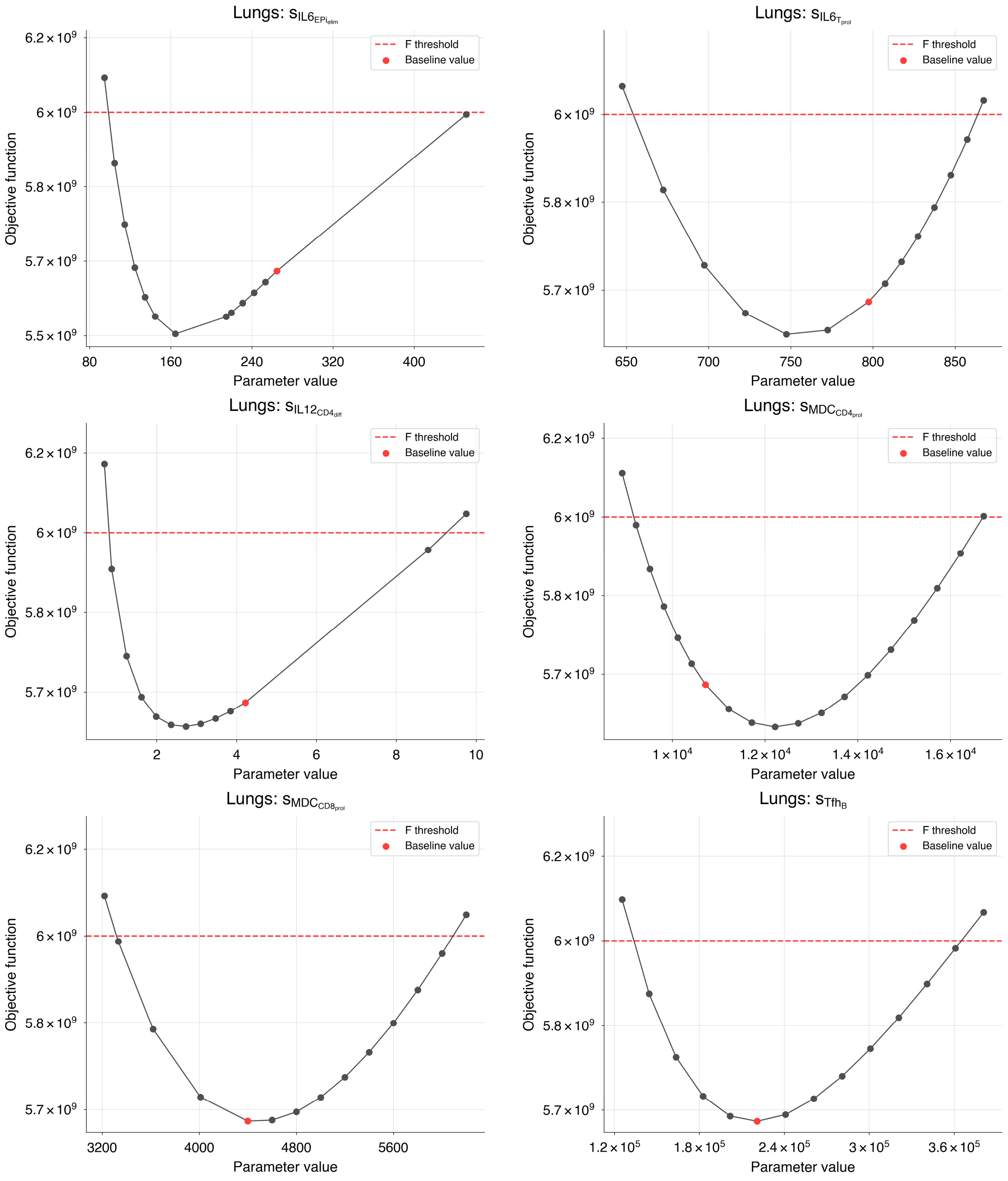

Lungs: $s_{IL6_{EPi_{elim}}}$
Lungs: $s_{IL6_{T_{prol}}}$
Lungs: $s_{IL12_{CD4_{diff}}}$
Lungs: $s_{MDC_{CD4_{prol}}}$
Lungs: $s_{MDC_{CD8_{prol}}}$
Lungs: s_{Tfh_B}
F threshold
Baseline value
Objective function
Parameter value
5.5×10^9
5.7×10^9
5.8×10^9
6×10^9
6.2×10^9
80
160
240
320
400
650
700
750
800
850
2
4
6
8
10
1×10^4
1.2×10^4
1.4×10^4
1.6×10^4
3200
4000
4800
5600
1.2×10^5
1.8×10^5
2.4×10^5
3×10^5
3.6×10^5

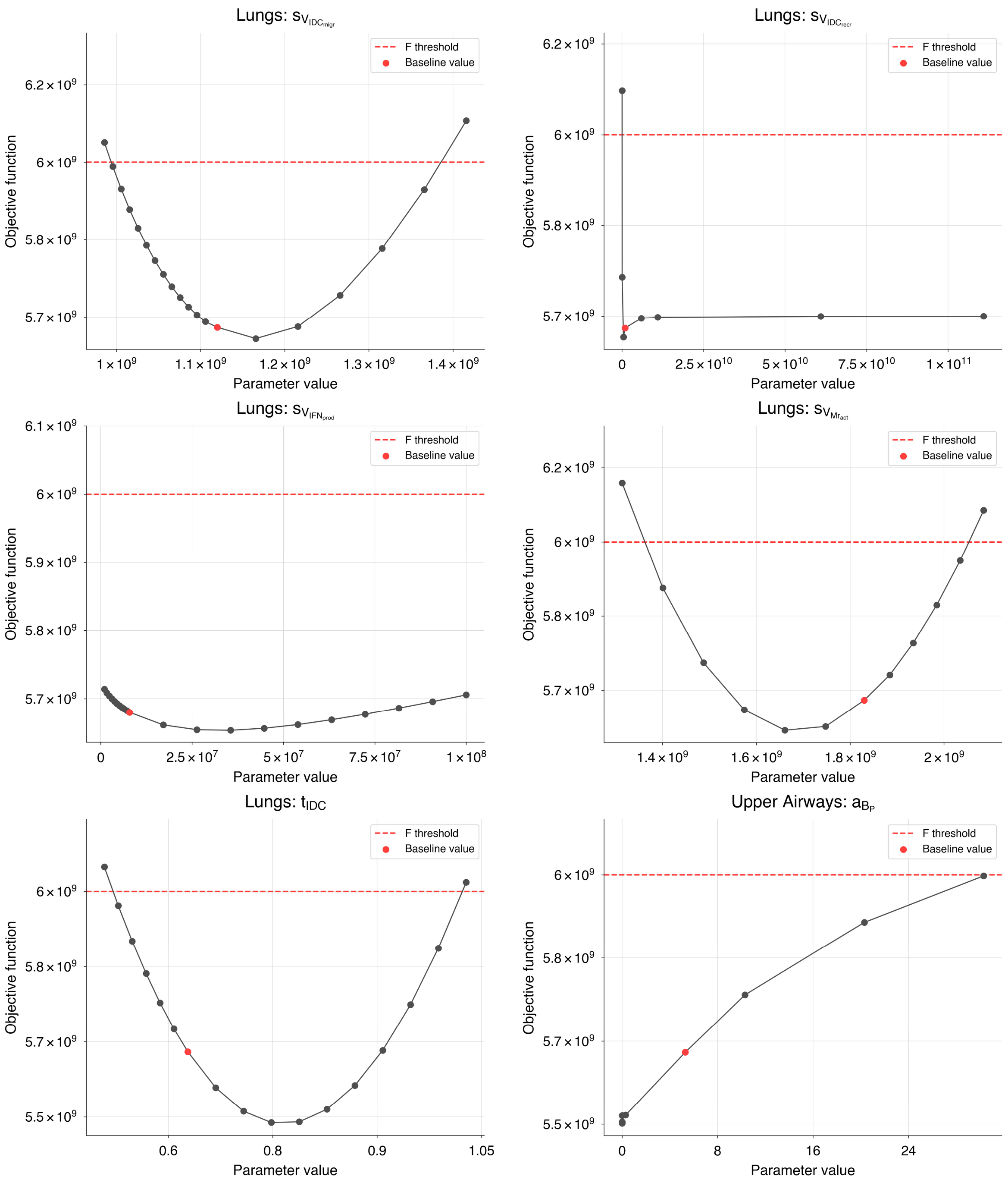

Lungs: $s_{V_{IDC_{migr}}}$
F threshold
Baseline value
Objective function
Parameter value
6.2×10^9
6×10^9
5.8×10^9
5.7×10^9
1×10^9
1.1×10^9
1.2×10^9
1.3×10^9
1.4×10^9
Lungs: $s_{V_{IDC_{recr}}}$
F threshold
Baseline value
Objective function
Parameter value
6.2×10^9
6×10^9
5.8×10^9
5.7×10^9
0
2.5×10^{10}
5×10^{10}
7.5×10^{10}
1×10^{11}
Lungs: $s_{V_{IFN_{prod}}}$
F threshold
Baseline value
Objective function
Parameter value
6.1×10^9
6×10^9
5.9×10^9
5.8×10^9
5.7×10^9
0
2.5×10^7
5×10^7
7.5×10^7
1×10^8
Lungs: $s_{V_{Mr_{act}}}$
F threshold
Baseline value
Objective function
Parameter value
6.2×10^9
6×10^9
5.8×10^9
5.7×10^9
1.4×10^9
1.6×10^9
1.8×10^9
2×10^9
Lungs: t_{IDC}
F threshold
Baseline value
Objective function
Parameter value
6×10^9
5.8×10^9
5.7×10^9
5.5×10^9
0.6
0.8
0.9
1.05
Upper Airways: a_{B_P}
F threshold
Baseline value
Objective function
Parameter value
6×10^9
5.8×10^9
5.7×10^9
5.5×10^9
0
8
16
24

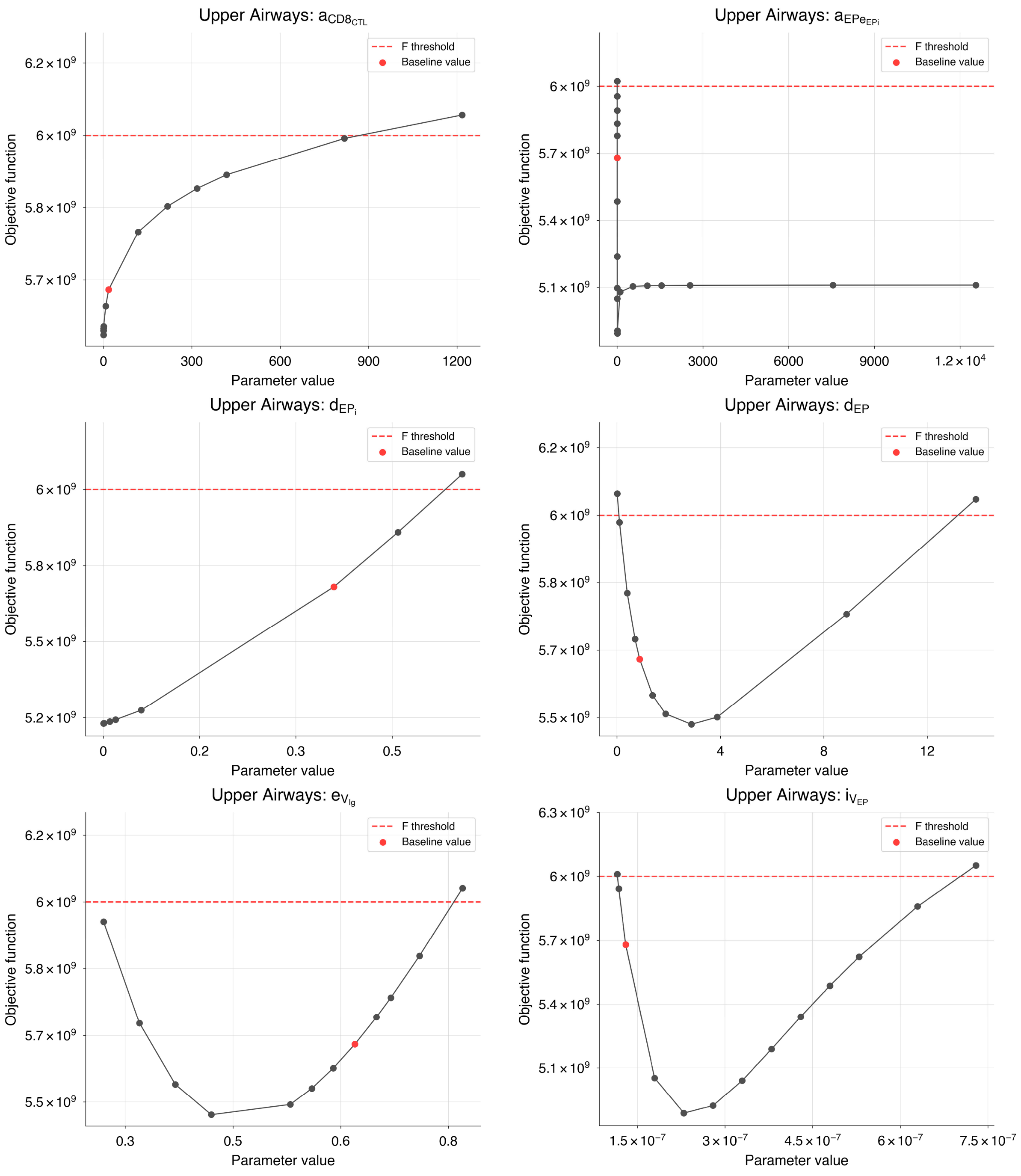

Upper Airways: $a_{CD8_{CTL}}$
Upper Airways: $a_{EPe_{EPi}}$
Upper Airways: d_{EP_i}
Upper Airways: d_{EP}
Upper Airways: $e_{V_{lg}}$
Upper Airways: $i_{V_{EP}}$
F threshold
Baseline value
Objective function
Parameter value
6.3×10^9
6.2×10^9
6×10^9
5.8×10^9
5.7×10^9
5.5×10^9
5.4×10^9
5.2×10^9
5.1×10^9
0
300
600
900
1200
3000
6000
9000
1.2×10^4
0.2
0.3
0.5
4
8
12
0.6
0.8
1.5×10^{-7}
3×10^{-7}
4.5×10^{-7}
6×10^{-7}
7.5×10^{-7}

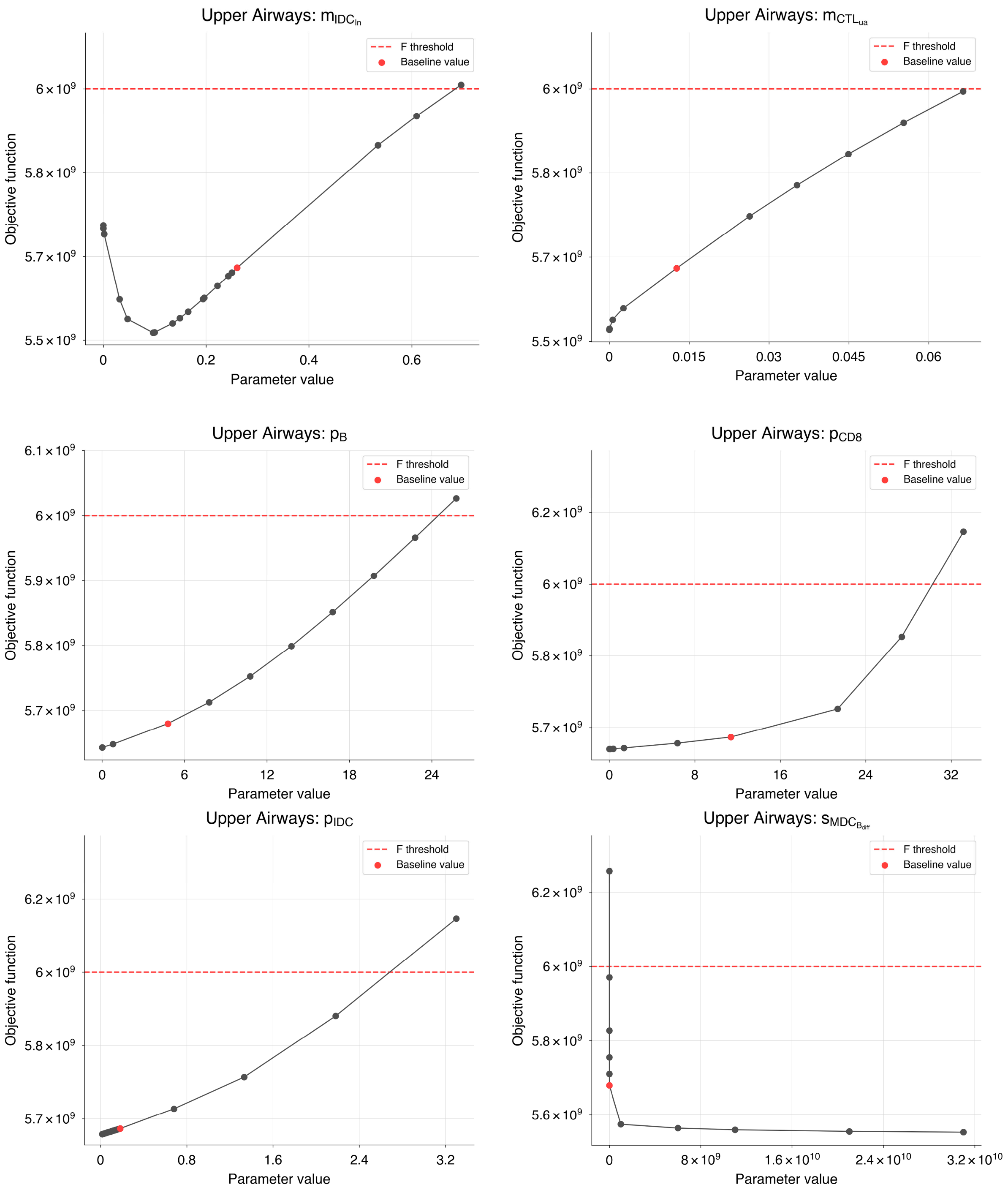

Upper Airways: $m_{IDC_{ln}}$
Upper Airways: $m_{CTL_{ua}}$
Upper Airways: p_B
Upper Airways: p_{CD8}
Upper Airways: p_{IDC}
Upper Airways: $s_{MDC_{B_{diff}}}$
F threshold
Baseline value
Objective function
Parameter value
5.5×10^9
5.6×10^9
5.7×10^9
5.8×10^9
5.9×10^9
6×10^9
6.1×10^9
6.2×10^9
0
0.2
0.4
0.6
0.015
0.03
0.045
0.06
6
12
18
24
8
16
32
0.8
1.6
2.4
3.2
8×10^9
1.6×10^{10}
2.4×10^{10}
3.2×10^{10}

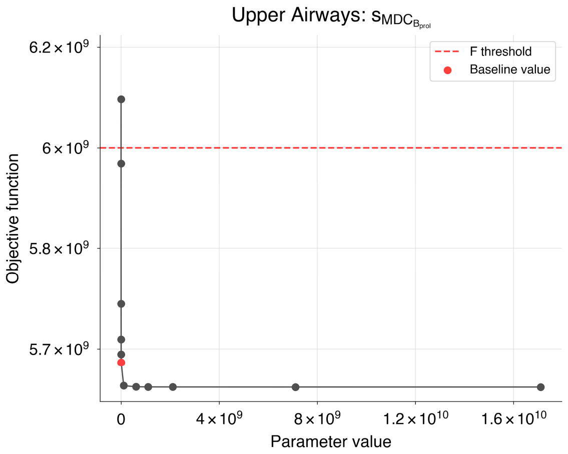
Upper Airways: $s_{MDC_{B_{prol}}}$
F threshold
Baseline value
Objective function
6.2×10^9
6×10^9
5.8×10^9
5.7×10^9
0
4×10^9
8×10^9
1.2×10^{10}
1.6×10^{10}
Parameter value

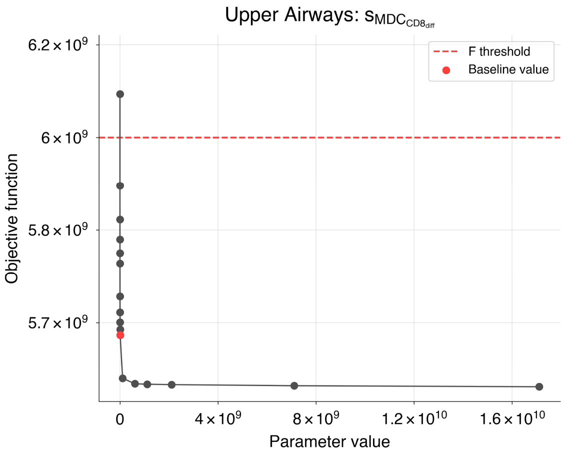
Upper Airways: $s_{MDC_{CD8_{diff}}}$
F threshold
Baseline value
Objective function
6.2×10^9
6×10^9
5.8×10^9
5.7×10^9
0
4×10^9
8×10^9
1.2×10^{10}
1.6×10^{10}
Parameter value

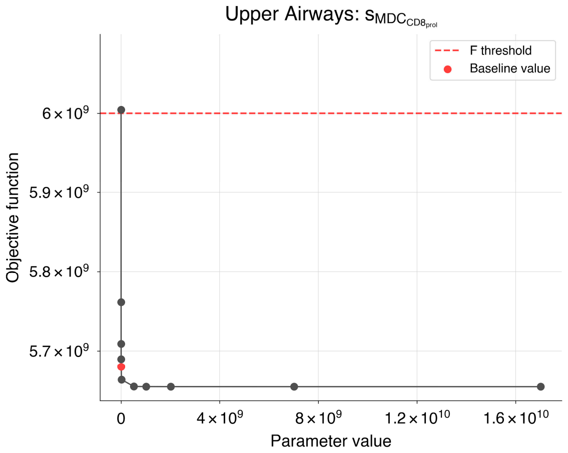
Upper Airways: $s_{MDC_{CD8_{prol}}}$
F threshold
Baseline value
Objective function
6×10^9
5.9×10^9
5.8×10^9
5.7×10^9
0
4×10^9
8×10^9
1.2×10^{10}
1.6×10^{10}
Parameter value

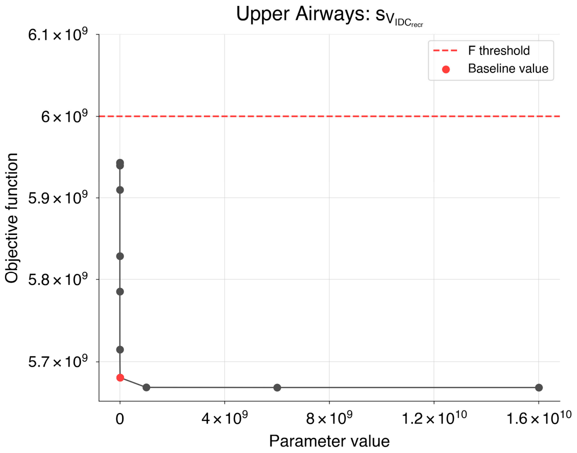
Upper Airways: $s_{V_{IDC_{recr}}}$
F threshold
Baseline value
Objective function
6.1×10^9
6×10^9
5.9×10^9
5.8×10^9
5.7×10^9
0
4×10^9
8×10^9
1.2×10^{10}
1.6×10^{10}
Parameter value

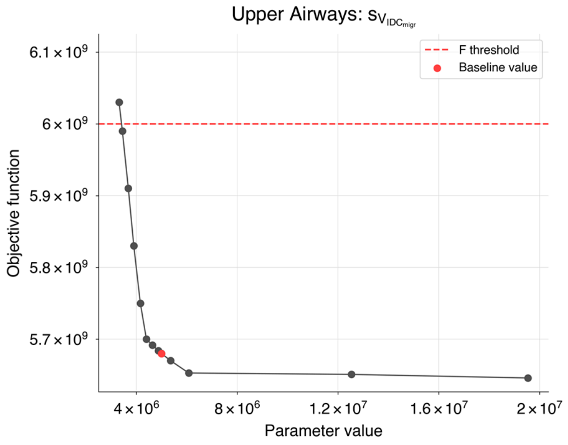
Upper Airways: $s_{V_{IDC_{migr}}}$
F threshold
Baseline value
Objective function
6.1×10^9
6×10^9
5.9×10^9
5.8×10^9
5.7×10^9
4×10^6
8×10^6
1.2×10^7
1.6×10^7
2×10^7
Parameter value
