# Supplementary material for: A Modular Mathematical Model of the Immune Response for Investigating the Pathogenesis of Infectious Diseases
Source: Viruses. 2025 Apr 22;17(5):589. doi: 10.3390/v17050589 (PMC12115727; doi:10.3390/v17050589)
Supplement: Supplementary file 1 [file viruses-17-00589-s001.zip › viruses-3549703-supplementary/Supplementary File S3. Model Simulations-tracked.pdf]

Baseline Solution for the Lung Compartment

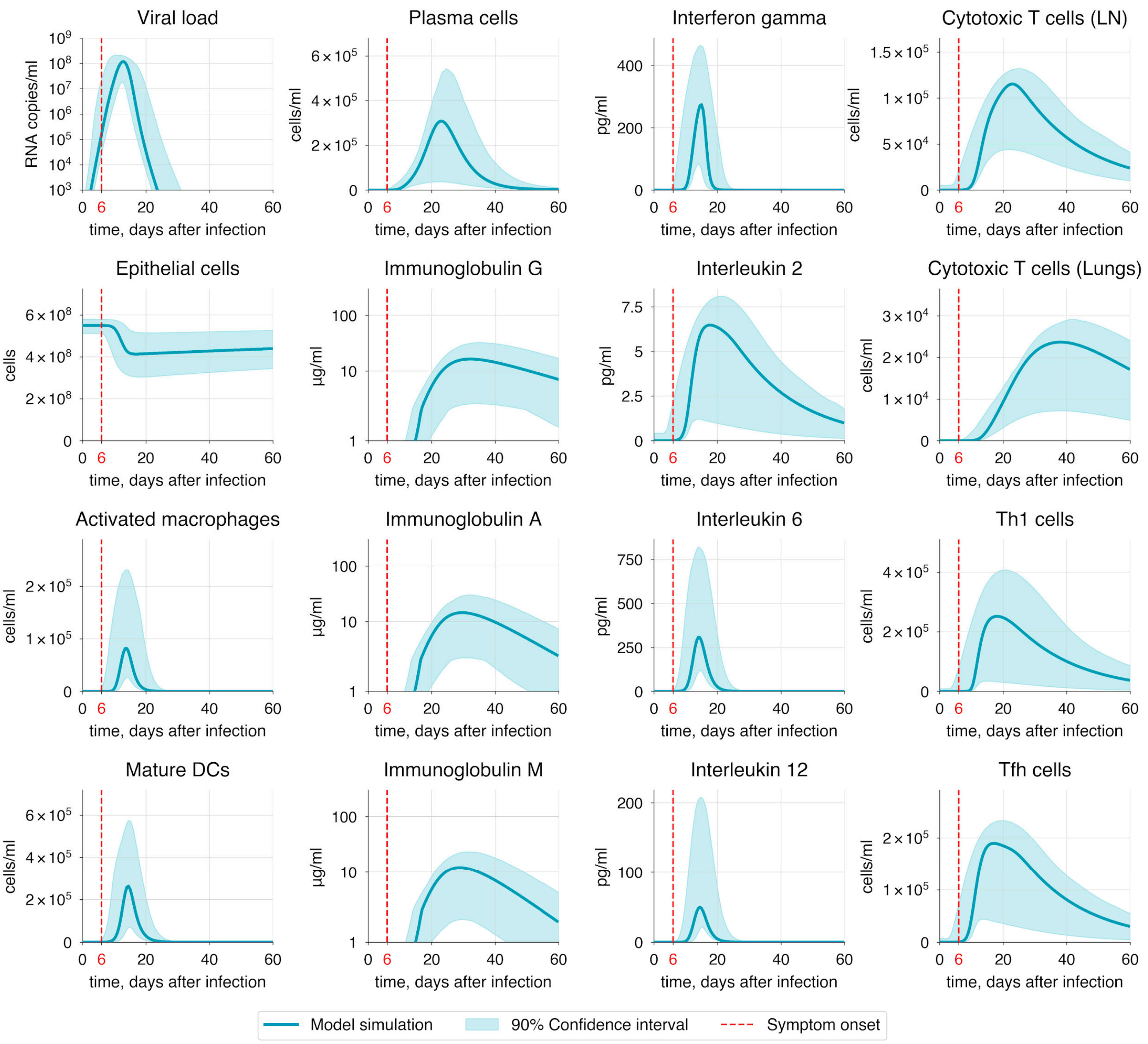


**Figure S1.** *Baseline solution for the lung compartment. The red line indicates the day of symptom onset. The blue shaded area represents the 90% confidence interval.*

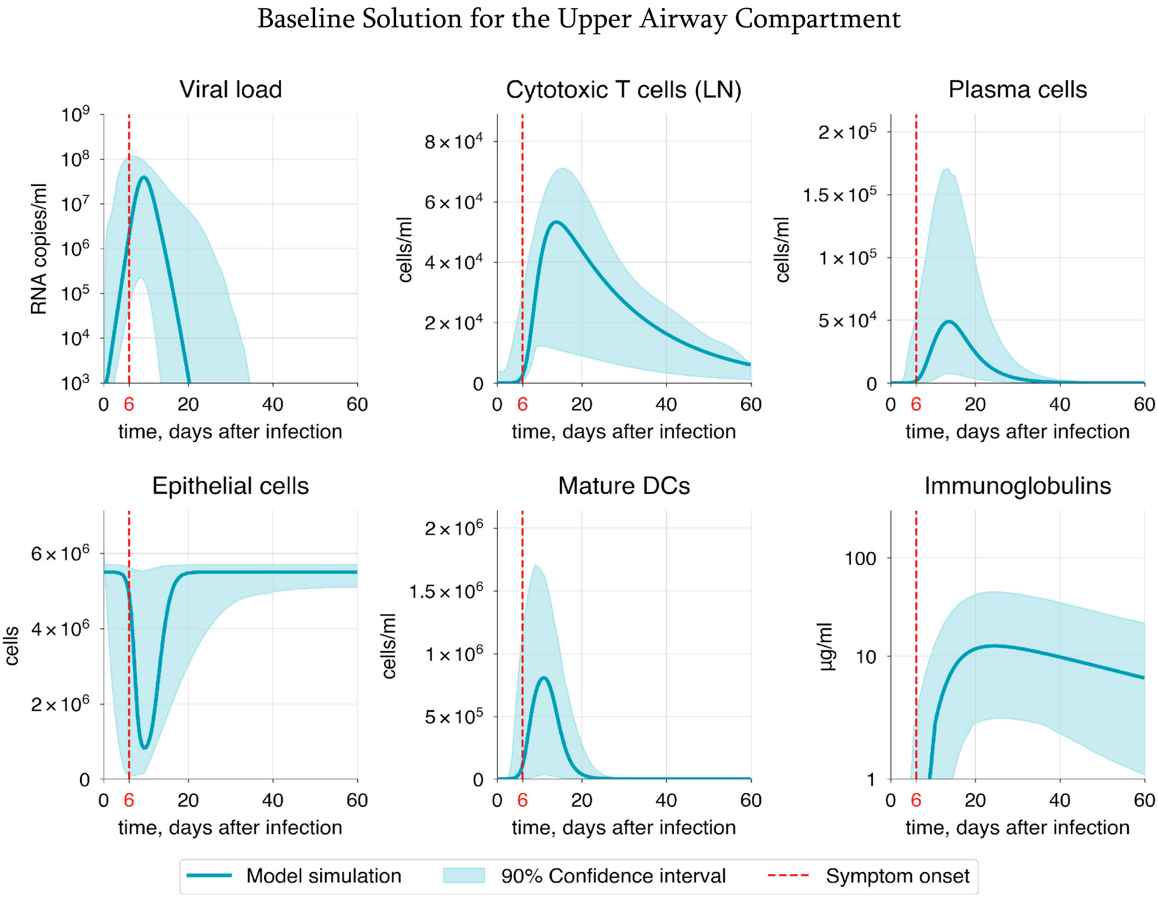


**Figure S2.** *Baseline solution for the upper airway compartment. The red line indicates the day of symptom onset. The blue shaded area represents the 90% confidence interval.*

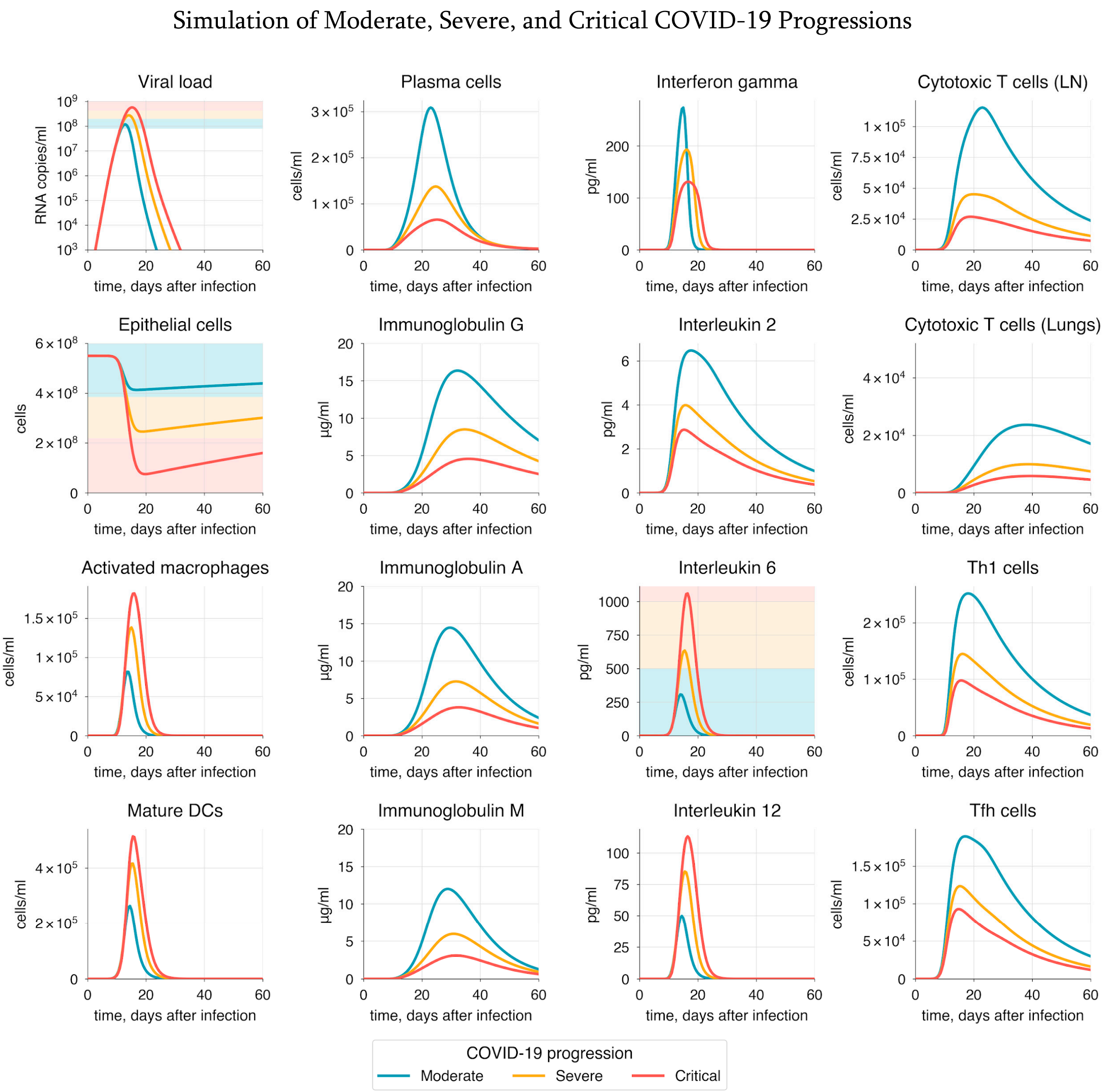


**Figure S3.** *Simulation of moderate, severe, and critical COVID-19 progressions. Shaded areas indicate severity levels: green for moderate, yellow for severe, and red for critical progression.*

# Baseline Model with Macrophage Hyperactivation

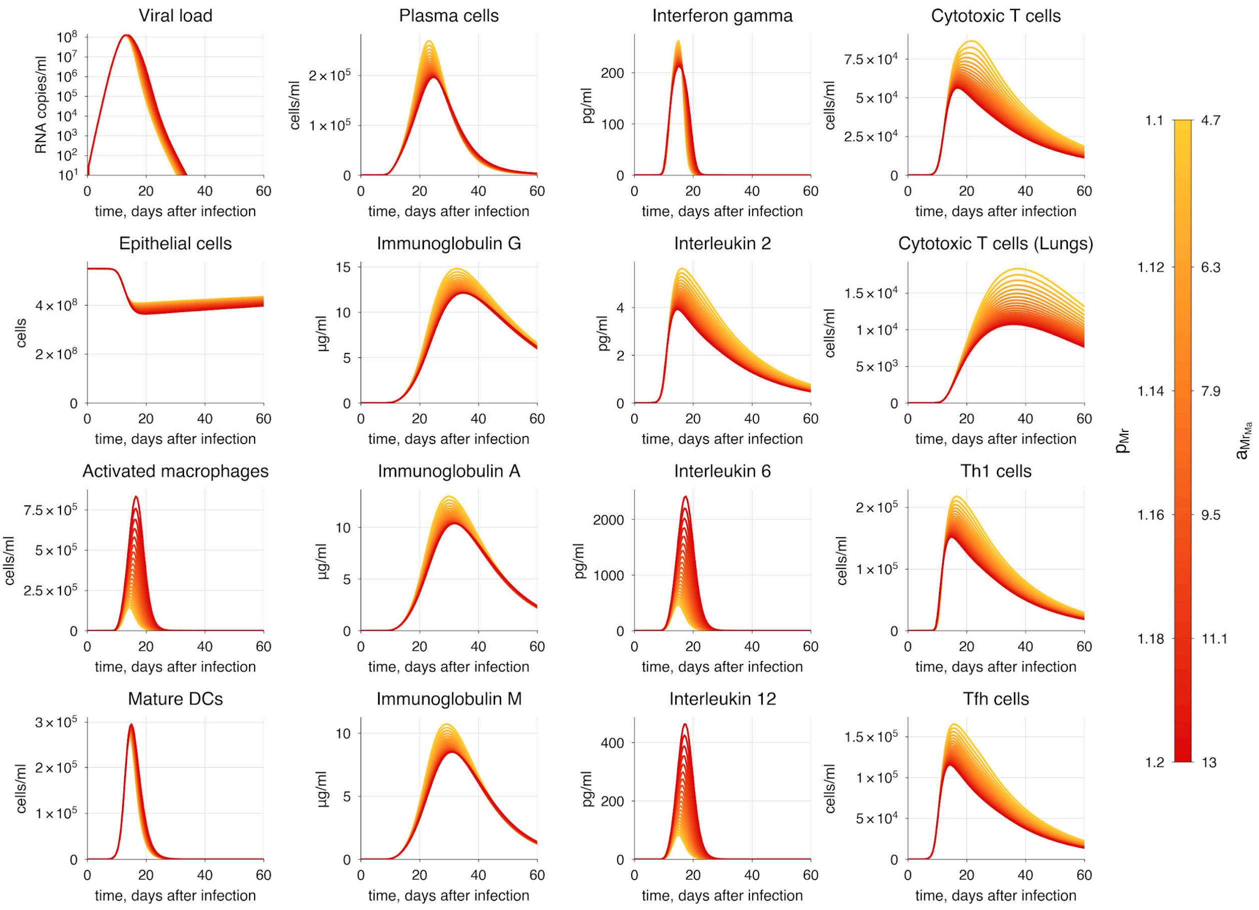


**Figure S4.** *Baseline model solution with macrophage hyperactivation. The varying parameters represent the rates of macrophage recruitment and activation. The transition from yellow to red indicates increasing severity of COVID-19.*

# Baseline Model with Innate Immune Response Delay

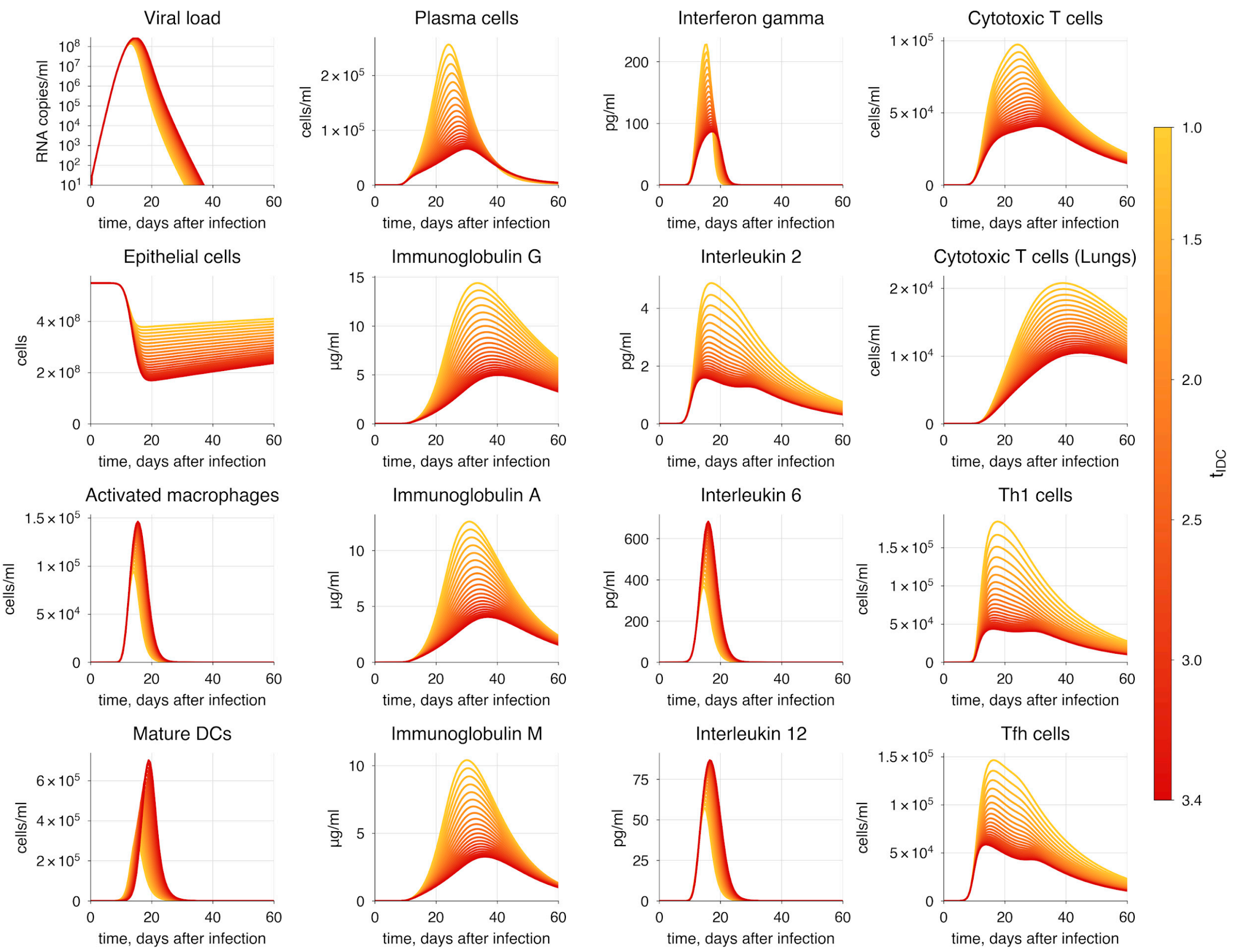


**Figure S5.** *Baseline model solution with innate immune response delay. The varying parameter represents the delay of dendritic cell maturation and migration to lymph nodes. The transition from yellow to red indicates increasing severity of COVID-19.*

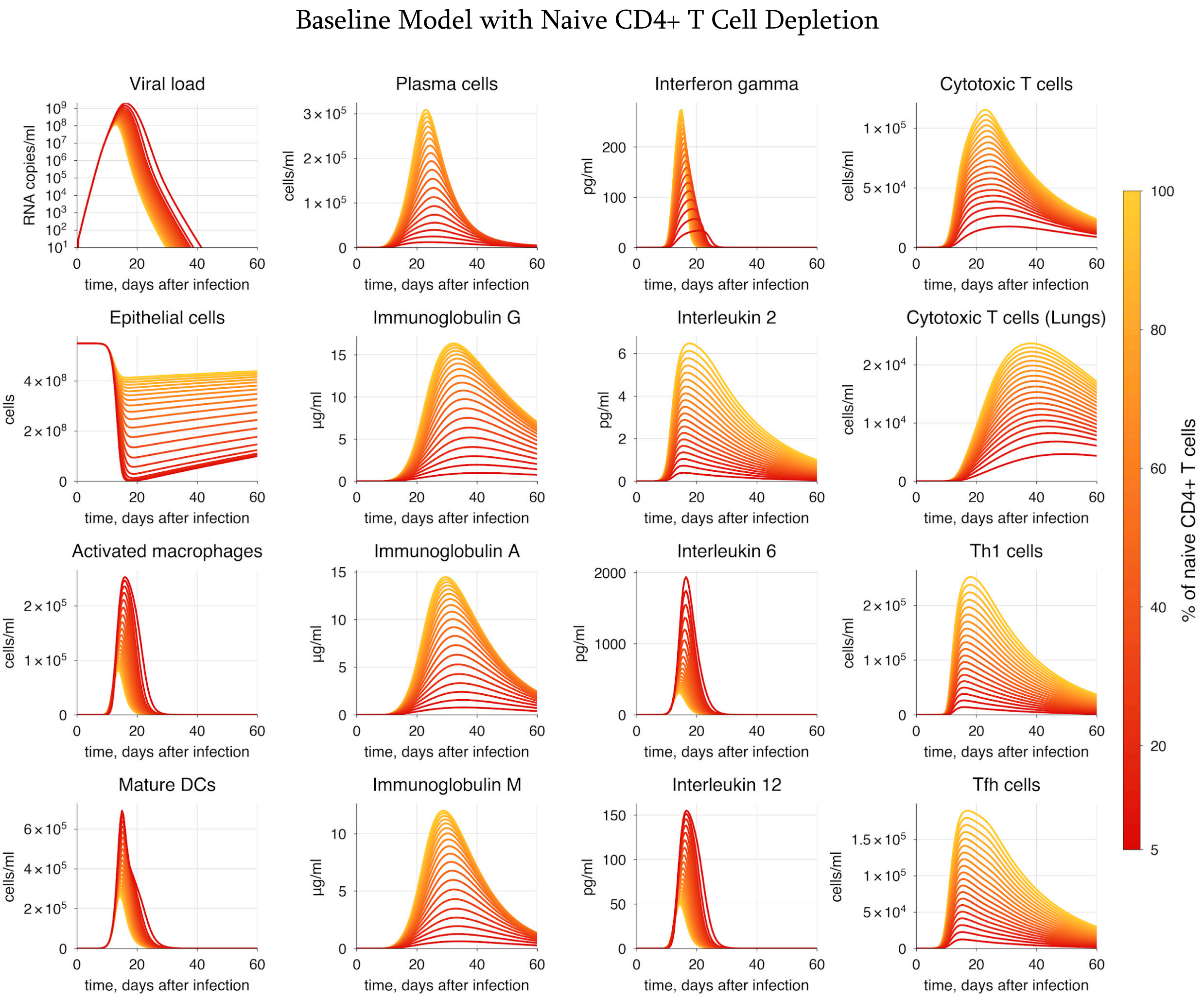


**Figure S6.** *Baseline model solution with naive CD4+ T cell depletion. The transition from yellow to red indicates increasing severity of COVID-19.*

# Baseline Model with Impaired T Cell Development Due to Immunosuppression

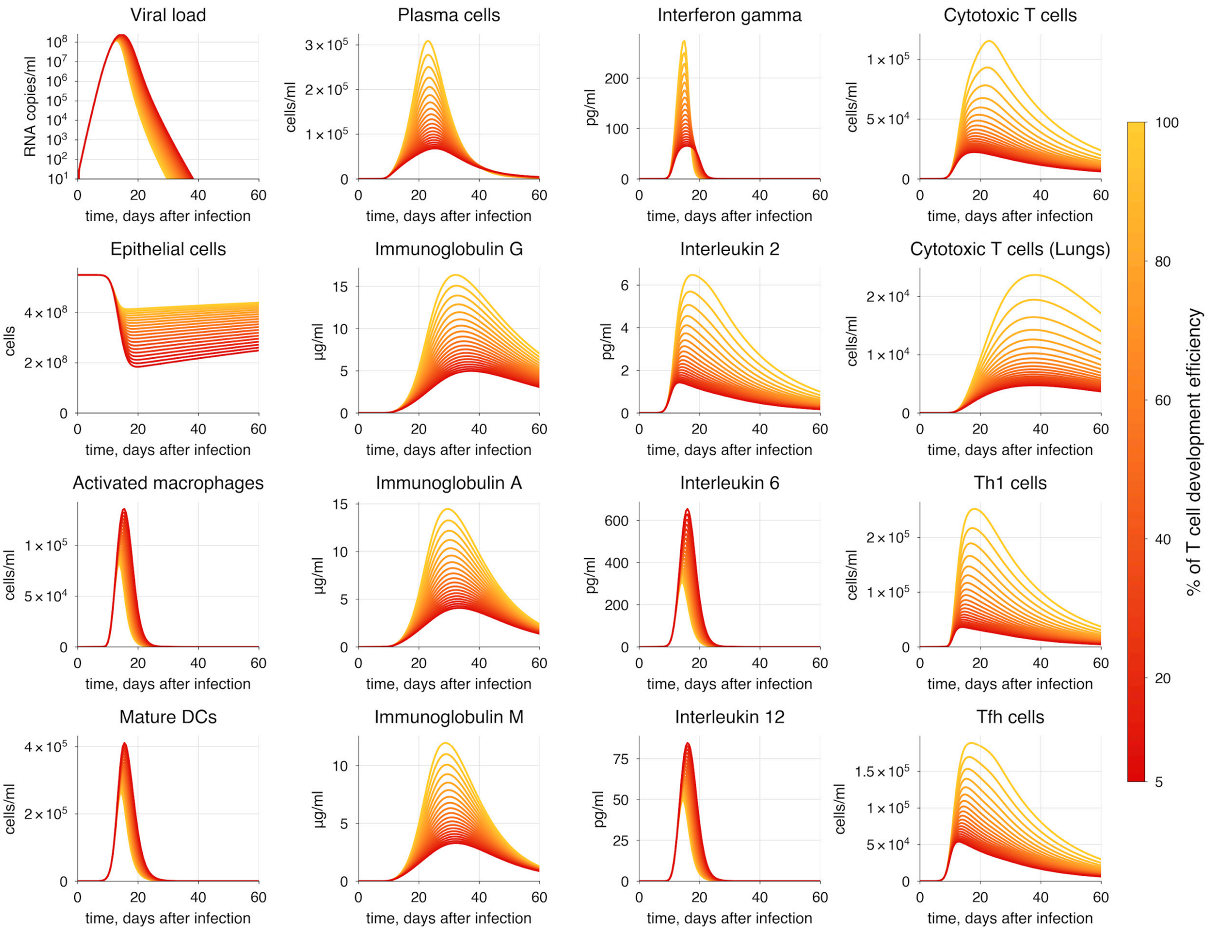


**Figure S7.** *Baseline model solution with impaired T cell development due to immunosuppression. The varying parameters represent the rates of CD4+ and CD8+ T cell proliferation. The transition from yellow to red indicates increasing severity of COVID-19.*

# Baseline Model with Enhanced Viral Infectivity and Immune Evasion

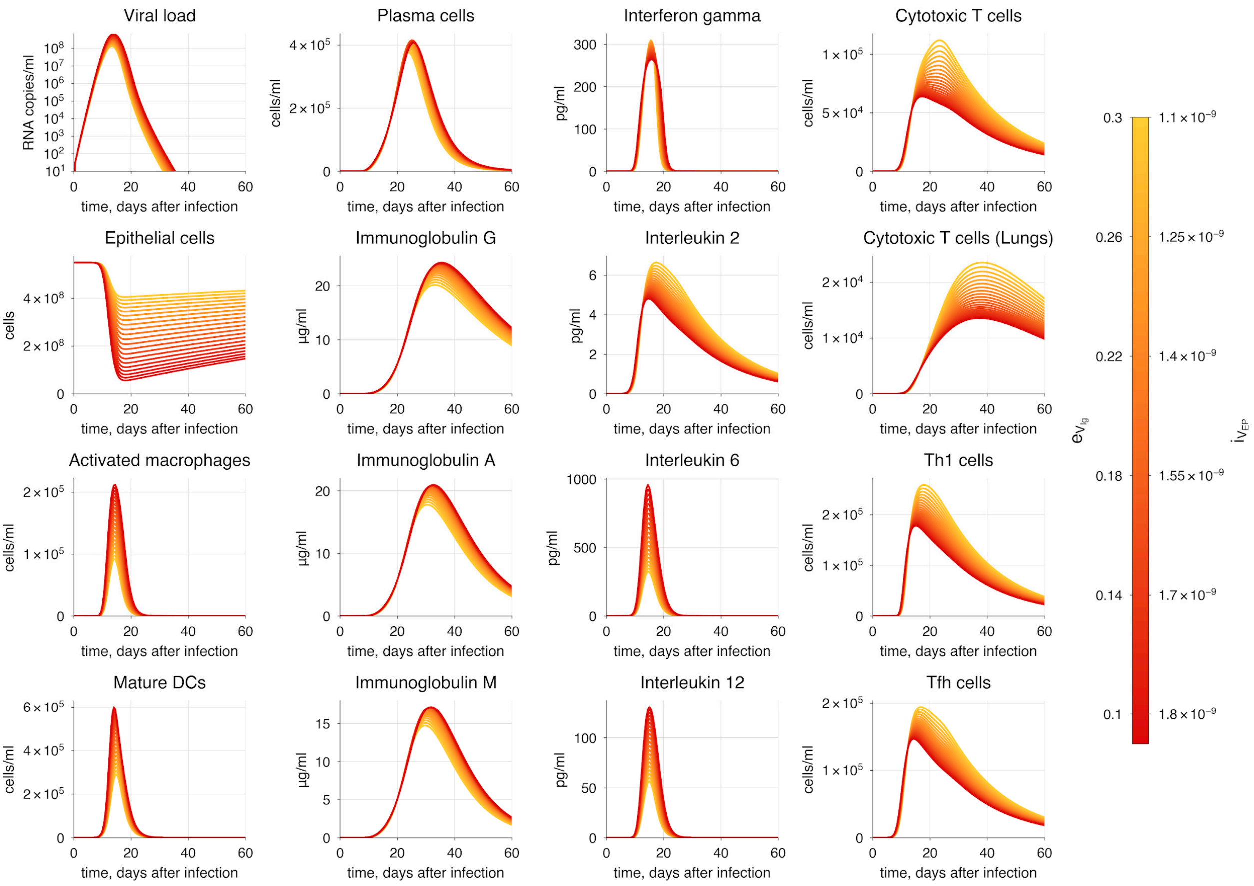


**Figure S8.** *Baseline model solution with enhanced viral infectivity and immune evasion. The varying parameters represent the rates of epithelial cell infection by the virus and virion neutralization by antibodies. The transition from yellow to red indicates increasing severity of COVID-19.*

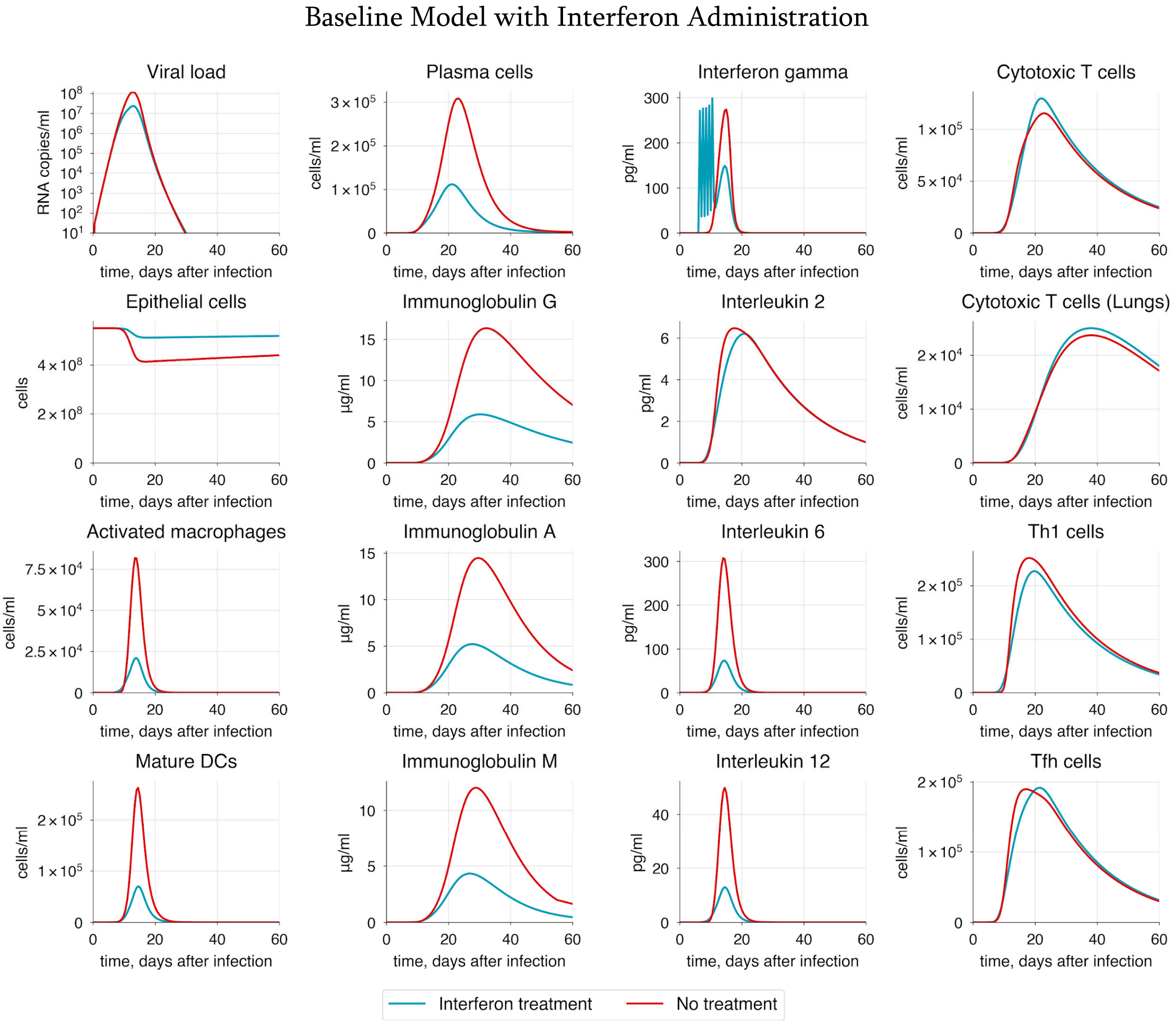


**Figure S9.** *Baseline model solution with interferon administration. Interferon is administered at a concentration of 2000 pg/mL daily for five days post symptom onset.*

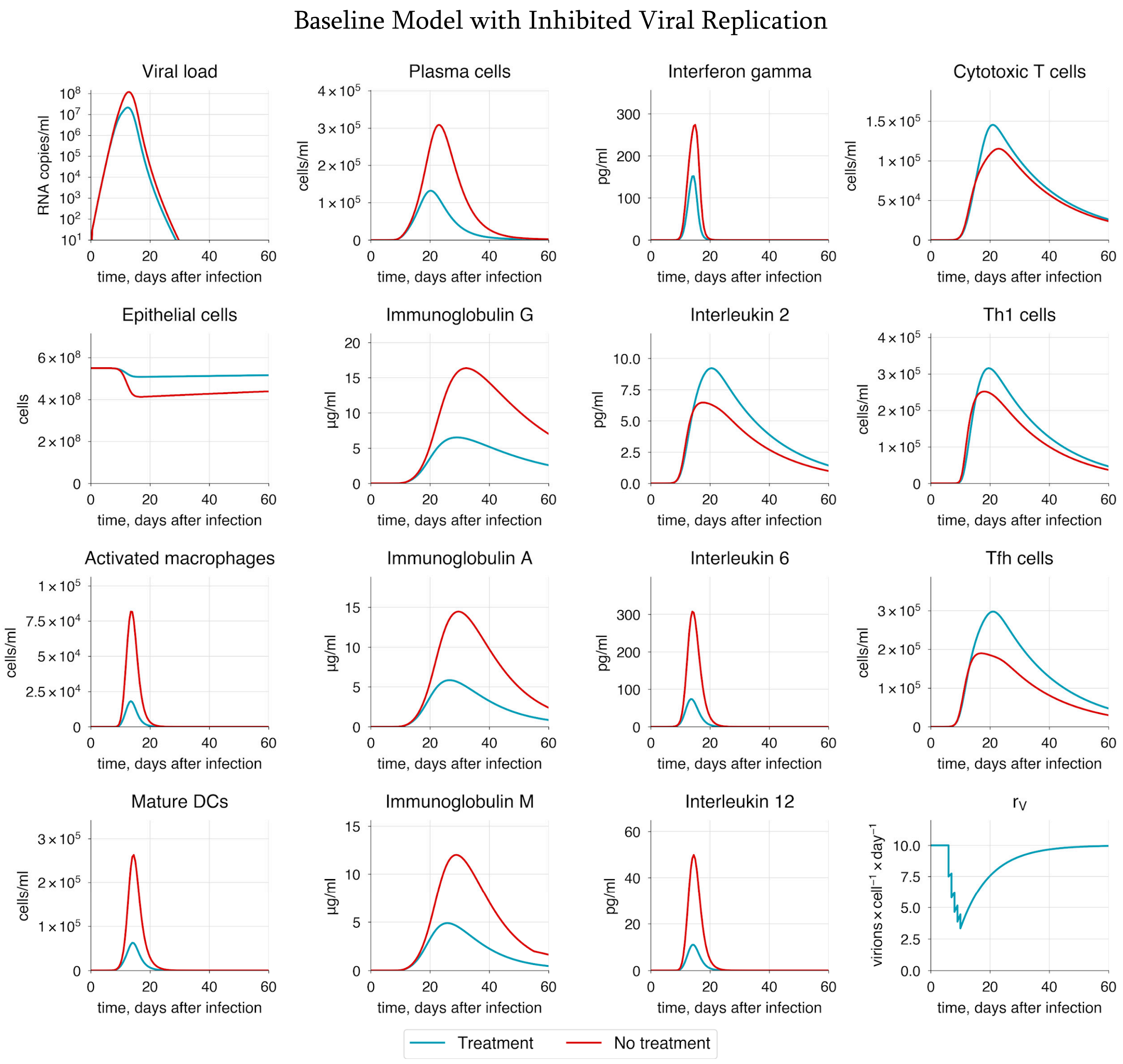


**Figure S10.** *Baseline model solution with inhibited viral replication. Drug is administered daily for five days post symptom onset.*
