# Supplementary material for: A Modular Mathematical Model of the Immune Response for Investigating the Pathogenesis of Infectious Diseases
Source: Viruses. 2025 Apr 22;17(5):589. doi: 10.3390/v17050589 (PMC12115727; doi:10.3390/v17050589)
Supplement: Supplementary file 1 [file viruses-17-00589-s001.zip › viruses-3549703-supplementary/Supplementary Table S1. Model Parameters-tracked.pdf]

Parameters taken from other mathematical models were labeled as “sourced,” with the corresponding references provided. If a parameter was estimated through model fitting to experimental data, we marked it as “estimated”; the data used for optimization are presented in Table S3. If a parameter was manually calculated based on constants from the literature, we labeled it as “calculated” and provided the corresponding references. The calculations are detailed in Section 2.2. Ranges of parameter values and identifiability analysis results for estimated parameters are given in the table.

In total, there are 112 parameters: 42 calculated, 59 estimated, and 11 sourced from other mathematical models.

Table S1. Model Parameters

**Upper Airways**					
**Notation**	**Parameter, Units**	**Value**	**Range**	**Identifiability**	**Reference**
$a_{EPe_{EPi}}$	Rate of epithelial cell transition from exposed to infected state, day^{-1}	1.52	(1.2, 10)	Left-identifiable	Estimated
d_{CTL}	Rate of natural decay for CTL, day^{-1}	0.05	(0.04, 0.06)	-	Calculated [1,2]
d_{EP}	Rate of natural decay for EP, day^{-1}	0.88	(0.1, 13)	Identifiable	Estimated
d_{EPi}	Rate of natural decay for EP_i, day^{-1}	0.36	(0.06, 0.5)	Right-identifiable	Estimated
d_{IDC}	Rate of natural decay for IDC, day^{-1}	0.13	(0.1, 0.15)	-	Calculated [3–5]
d_V	Rate of natural decay for V, day^{-1}	0.5	(0.4, 0.5)	-	Calculated [6]
$e_{EPi_{CTL}}$	Rate of infected epithelial cell elimination by CTL, $(cells/mL)^{-1}day^{-1}$	0.001	(0.0008, 0.0012)	-	Sourced [7]
$e_{V_{Ig}}$	Rate of free virion neutralization by immunoglobulins, $(\mu g/mL)^{-1}day^{-1}$	0.62	(0.3, 0.7)	Identifiable	Estimated
$i_{V_{EP}}$	Rate of epithelial cell infection by SARS-CoV-2, $(virions/mL)^{-1}day^{-1}$	1.3e-7	(1.15e-7, 7e-7)	Identifiable	Estimated
$m_{CTL_{ua}}$	Rate of CTL migration to the upper airways, day^{-1}	0.0125	(0.005, 0.06)	Right-identifiable	Estimated
p_{IDC}	Maximum rate of IDC recruitment due to infection, day^{-1}	0.18	(0.1, 3)	Right-identifiable	Estimated

$r_{V_{EPi}}$	Rate of virion production by EP_i, $(virions/mL)\ cells^{-1}day^{-1}$	10	(8, 12)	-	Calculated [8–12]
$s_{V_{IDC_{recr}}}$	Half-saturation of virions in IDC recruitment, $virions/m$L	7.65e6	(600, 1e7)	Left-identifiable	Estimated
t_{CTL}	Time delay in CTL migration to the upper airways, day	0.5	(0.4, 0.6)	-	Sourced [7]
EP_0	Initial number of EP, $cells$	5.5e6	(5.1e6, 5.8e6)	-	Calculated [13–15]
IDC_0	Initial number of IDC, $cells/m$L	2.5e6	(2.3e6, 2.66e6)	-	Calculated [16–19]

Upper Airways (Lymph Nodes)					
**Notation**	**Parameter, Units**	**Value**	**Range**	**Identifiability**	**Reference**
a_{B_P}	Maximum rate of B_n differentiation into plasma cells, day^{-1}	5.3	(0.5, 30)	Right-identifiable	Estimated
$a_{CD8_{CTL}}$	Maximum rate of T_n differentiation into CTLs, day^{-1}	17.65	(10, 800)	Right-identifiable	Estimated
d_B	Rate of natural decay for B_n, day^{-1}	0.008	(0.0064, 0.096)	-	Calculated [20]
d_{CD8}	Rate of natural decay for T_n, day^{-1}	0.008	(0.0064, 0.096)	-	Calculated [21]
d_{CTL}	Rate of natural decay for CTL, day^{-1}	0.05	(0.04, 0.06)	-	Calculated [1,2]
d_{Ig}	Rate of natural decay for Ig, day^{-1}	0.025	(0.02, 0.03)	-	Calculated [22]
d_{MDC}	Rate of natural decay for MDC, day^{-1}	0.5	(0.4, 0.6)	-	Sourced [7]
d_P	Rate of natural decay for P, day^{-1}	0.2	(0.16, 0.24)	-	Calculated [23,24]
$m_{IDC_{ln}}$	Maximum rate of IDC maturation and migration to lymph nodes, day^{-1}	0.26	(0.001, 0.65)	Identifiable	Estimated
p_B	Maximum rate of B_n proliferation, day^{-1}	4.78	(1, 23)	Right-identifiable	Estimated
p_{CD8}	Maximum rate of T_n proliferation, day^{-1}	11.4	(6, 30)	Right-identifiable	Estimated
r_{Ig}	Rate of immunoglobulin production, $(\mu g/ml)\ (cells/mL)^{-1} day^{-1}$	3e-5	(2.4e-5, 3.6e-5)	-	Calculated [25–34]
$s_{MDCB_{diff}}$	Half-saturation of MDC in B_n differentiation, $cells/m$L	7.35e6	(1.3e6, 1e7)	Left-identifiable	Estimated
$s_{MDCB_{prol}}$	Half-saturation of MDC in B_n proliferation, $cells/m$L	7.25e6	(1.2e6, 1e7)	Left-identifiable	Estimated
$s_{MDCCD8_{diff}}$	Half-saturation of MDC in T_n differentiation, $cells/m$L	8.85e6	(4e5, 1e7)	Left-identifiable	Estimated
$s_{MDCCD8_{prol}}$	Half-saturation of MDC in T_n proliferation, $cells/m$L	7e6	(1e6, 1e7)	Left-identifiable	Estimated
$s_{VIDC_{migr}}$	Half-saturation of virions in IDC maturation and migration, $virions/m$L	5e6	(3.3e6, 6e6)	Left-identifiable	Estimated
B_0	Initial number of B_n, $cells/m$L	3.3e4	(3.1e4, 3.5e4)	-	Calculated [35,36]
$CD8_0$	Initial number of T_n, $cells/m$L	1.6e4	(1.5e4, 1.7e4)	-	Calculated [35,37,38]

Lungs					
**Notation**	**Parameter, Units**	**Value**	**Range**	**Identifiability**	**Reference**
$a_{EPe_{EPi}}$	Rate of epithelial cell transition from exposed to infected state, day^{-1}	0.64	(0.52, 0.82)	Identifiable	Estimated
$a_{Ma_{Mr}}$	Rate of M_a deactivation, day^{-1}	0.36	(0.29, 0.43)	-	Sourced [39]
$a_{Mr_{Ma}}$	Maximum rate of M_r activation, day^{-1}	3.61	(3.3, 4.7)	Identifiable	Estimated
d_{CTL}	Rate of natural decay for CTL, day^{-1}	0.05	(0.04, 0.06)	-	Calculated [1,2]
d_{EP}	Rate of natural decay for EP, day^{-1}	0.005	(0.004, 0.006)	-	Sourced [7]
d_{EPi}	Rate of natural decay for EP_i, day^{-1}	0.5	(0.4, 0.6)	-	Sourced [40]
d_{IDC}	Rate of natural decay for IDC, day^{-1}	0.13	(0.1, 0.15)	-	Calculated [3–5]
d_{Ma}	Rate of natural decay for M_a, day^{-1}	0.3	(0.24, 0.36)	-	Calculated [41]
d_{Mr}	Rate of natural decay for M_r, day^{-1}	0.02	(0.016, 0.024)	-	Calculated [42]
d_V	Rate of natural decay for V, day^{-1}	0.5	(0.4, 0.6)	-	Calculated [6]
$e_{EPi_{CTL}}$	Maximum rate of infected epithelial cell elimination by CTL, $(cells/mL)^{-1}day^{-1}$	0.0039	(0.0024, 0.0045)	Identifiable	Estimated
$e_{V_{IgA}}$	Rate of free virion neutralization by IgA, $(\mu g/mL)^{-1}day^{-1}$	0.62	(0.3, 0.7)	Identifiable	Estimated
$e_{V_{IgG}}$	Rate of free virion neutralization by IgG, $(\mu g/mL)^{-1}day^{-1}$	0.62	(0.3, 0.7)	Identifiable	Estimated
$e_{V_{IgM}}$	Rate of free virion neutralization by IgM, $(\mu g/mL)^{-1}day^{-1}$	0.62	(0.3, 0.7)	Identifiable	Estimated
$i_{V_{EP}}$	Maximum rate of epithelial cell infection by SARS-CoV-2, $(virions/mL)^{-1}day^{-1}$	1.19e-9	(1.14e-9, 1.35e-9)	Identifiable	Estimated
$m_{CTL_{lungs}}$	Rate of CTL migration to the lungs, day^{-1}	0.018	(0.012, 0.021)	Identifiable	Estimated
$m_{V_{lungs}}$	Rate of V migration to the lungs, day^{-1}	0.075	(0.05, 2)	Identifiable	Estimated
p_{IDC}	Maximum rate of IDC recruitment due to infection, day^{-1}	0.145	(0.1, 2.2)	Right-identifiable	Estimated
p_{Mr}	Rate of M_r recruitment due to infection, day^{-1}	0.215	(0.15, 1.1)	Right-identifiable	Estimated

$r_{V_{EPi}}$	Rate of virion production by infected epithelial cells, $(virions/mL)\ cells^{-1}day^{-1}$	10	(8, 12)	-	Calculated [8–12,43]
$s_{IFN_{EP_{inf}}}$	Half-saturation of IFN_g in epithelial cell infection, pg/mL	180	(120, 1000)	Identifiable	Estimated
$s_{IFN_{Mr_{act}}}$	Half-saturation of IFN_g in macrophage activation, pg/mL	4	(0.1, 10)	Right-identifiable	Estimated
$s_{IL6_{EPi_{elim}}}$	Half-saturation of IL_6 in epithelial cell elimination, pg/mL	260	(100, 450)	Identifiable	Estimated
$s_{V_{IDC_{recr}}}$	Half-saturation of virions in IDC recruitment, $virions/mL$	9.3e8	(1e7, 6e9)	Left-identifiable	Estimated
$s_{V_{Mr_{act}}}$	Half-saturation of virions in M_r activation, $virions/mL$	1.8e9	(1.3e9, 2e9)	Identifiable	Estimated
t_{CTL}	Time delay in CTL migration to the lungs, day	0.5	(0.4, 0.6)	-	Sourced [7]
EP_0	Initial number of EP, $cells$	5.5e8	(5.1e8, 5.8e8)	-	Calculated [35,44–48]
IDC_0	Initial number of IDC, $cells/mL$	1.2e6	(1.1e6, 1.27e6)	-	Calculated [16,19,49–55]
M_0	Initial number of M_r, $cells/mL$	4e5	(3.7e5, 4.2e5)	-	Calculated [35,56,57]

Lungs (Lymph Nodes)					
Notation	Parameter, Units	Value	Range	Identifiability	Reference
a_{B_P}	Maximum rate of B_n differentiation into plasma cells, day^{-1}	0.82	(0.65, 1)	-	Sourced [40]
$a_{CD4_{Tfh}}$	Maximum rate of H_n differentiation into Tfh, day^{-1}	0.39	(0.22, 0.8)	Identifiable	Estimated
$a_{CD4_{Th1}}$	Maximum rate of H_n differentiation into Th1, day^{-1}	0.74	(0.6, 1.2)	Identifiable	Estimated
$a_{CD8_{CTL}}$	Maximum rate of T_n differentiation into CTL, day^{-1}	3.7	(3.6, 3.8)	Identifiable	Estimated
d_B	Rate of natural decay for B_n, day^{-1}	0.008	(0.0064, 0.0096)	-	Calculated [20]
d_{CTL}	Rate of natural decay for CTL, day^{-1}	0.05	(0.04, 0.06)	-	Calculated [1,2]
d_{IFN}	Rate of natural decay for IFN_g, day^{-1}	4	(3.2, 4.8)	-	Calculated [58]
d_{IL12}	Rate of natural decay for IL_{12}, day^{-1}	1.4	(1.12, 1.68)	-	Calculated [59]
d_{IL2}	Rate of natural decay for IL_2, day^{-1}	15	(12, 18)	-	Calculated [60]
d_{IL6}	Rate of natural decay for IL_6, day^{-1}	1	(0.8, 1.2)	-	Calculated [57]
d_{IgA}	Rate of natural decay for IgA, day^{-1}	0.08	(0.064, 0.096)	-	Calculated [61]
d_{IgG}	Rate of natural decay for IgG, day^{-1}	0.04	(0.032, 0.048)	-	Calculated [22]
d_{IgM}	Rate of natural decay for IgM, day^{-1}	0.1	(0.08, 0.12)	-	Calculated [62]
d_{MDC}	Rate of natural decay for MDC, day^{-1}	0.5	(0.4, 0.6)	-	Sourced [7]
d_P	Rate of natural decay for P, day^{-1}	0.2	(0.16, 0.24)	-	Calculated [23,24]
d_T	Rate of natural decay for H_n and T_n, day^{-1}	0.008	(0.0065, 0.0096)	-	Calculated [21]
d_{Tfh}	Rate of natural decay for Tfh, day^{-1}	0.05	(0.04, 0.06)	-	Calculated [1,2]
d_{Th1}	Rate of natural decay for Th1, day^{-1}	0.05	(0.04, 0.06)	-	Calculated [1,2]
$m_{IDC_{ln}}$	Maximum rate of IDC maturation and migration to lymph nodes, day^{-1}	1.94	(1.55, 2.15)	Identifiable	Estimated
p_B	Maximum rate of B_n proliferation, day^{-1}	1.39	(1.26, 1.6)	Identifiable	Estimated

p_{CD4}	Maximum rate of H_n proliferation, day^{-1}	1	(0.86, 1.07)	Identifiable	Estimated
p_{CD8}	Maximum rate of T_n proliferation, day^{-1}	3.94	(3.81, 4)	Identifiable	Estimated
$r_{IFN_{CTL}}$	Maximum rate of IFN_g production by CTL, $(pg/ml)\ (cells/mL)^{-1}day^{-1}$	0.0065	(0.005, 0.019)	Right-identifiable	Estimated
$r_{IFN_{Th1}}$	Maximum rate of IFN_g production by Th1, $(pg/ml)\ (cells/mL)^{-1}day^{-1}$	0.0037	(0.003, 0.0075)	Right-identifiable	Estimated
$r_{IL12_{MDC}}$	Rate of IL_{12} production by MDC, $(pg/ml)\ (cells/mL)^{-1}day^{-1}$	3.4e-5	(3e-5, 7.5e-4)	Right-identifiable	Estimated
$r_{IL12_{Ma}}$	Rate of IL_{12} production by M_a, $(pg/ml)\ (cells/mL)^{-1}day^{-1}$	8e-4	(6.4e-4, 9.6e-4)	-	Sourced [39]
$r_{IL2_{Tfh}}$	Rate of IL_2 production by Tfh, $(pg/mL)\ (cells/mL)^{-1}day^{-1}$	2.85e-4	(1.9e-4, 3.2e-4)	Identifiable	Estimated
$r_{IL2_{Th1}}$	Rate of IL_2 production by Th1, $(pg/mL)\ (cells/mL)^{-1}day^{-1}$	1.7e-4	(1.5e-5, 2.3e-4)	Identifiable	Estimated
$r_{IL6_{EPi}}$	Rate of IL_6 production by EP_i, $(pg/mL)\ cells^{-1}day^{-1}$	8.45e-6	(6.2e-6, 1.5e-5)	Identifiable	Estimated
$r_{IL6_{Ma}}$	Rate of IL_6 production by M_a, $(pg/mL)\ (cells/mL)^{-1}day^{-1}$	0.0029	(2.65e-3, 3.8e-3)	Identifiable	Estimated
r_{IgA_P}	Rate of IgA production, $(\mu g/mL)\ (cells/mL)^{-1}day^{-1}$	7e-6	(5.6e-6, 8.4e-6)	-	Calculated [26,28,32–34,38]
r_{IgG_P}	Rate of IgG production, $(\mu g/mL)\ (cells/mL)^{-1}day^{-1}$	6e-6	(4.8e-6, 7.2e-6)	-	Calculated [26,28,32–34,38]
r_{IgM_P}	Rate of IgM production, $(\mu g/mL)\ (cells/mL)^{-1}day^{-1}$	6.5e-6	(5.2e-6, 7.8e-6)	-	Calculated [26,28,32–34,38]
$s_{IL12_{CD4_{diff}}}$	Half-saturation of IL_{12} in H_n differentiation to Th1, pg/mL	4.22	(0.85, 9.7)	Identifiable	Estimated
$s_{IL2_{T_{diff}}}$	Half-saturation of IL_2 in T_n differentiation to CTL, pg/mL	18.35	(17.5, 18.8)	Identifiable	Estimated
$s_{IL2_{T_{prol}}}$	Half-saturation of IL_2 in T_n proliferation, pg/mL	17.6	(17.25, 18.35)	Identifiable	Estimated
$s_{IL6_{CD4_{diff}}}$	Half-saturation of IL_6 in H_n differentiation to Tfh, pg/mL	4.5	(2, 12)	Identifiable	Estimated
$s_{IL6_{T_{prol}}}$	Half-saturation of IL_6 in T_n proliferation, pg/mL	800	(660, 860)	Identifiable	Estimated
$s_{MDC_{B_{prol}}}$	Half-saturation of MDC in B_n proliferation, $cells/m$L	1.8e4	(1.44e4, 2.1e4)	-	Sourced [7]
$s_{MDC_{CD4_{prol}}}$	Half-saturation of MDC in H_n proliferation, $cells/m$L	1.07e4	(9e3, 1.67e4)	Identifiable	Estimated

$s_{MDC_{CD8_{prol}}}$	Half-saturation of MDC in T_n proliferation, $cells/m$L	4400	(3300, 6000)	Identifiable	Estimated
s_{Tfh_B}	Half-saturation of Tfh in B_n proliferation and differentiation to P, $cells/m$L	2.2e5	(1.4e5, 3.6e5)	Identifiable	Estimated
$s_{V_{IDC_{migr}}}$	Half-saturation of virions in IDC maturation and migration, $virions/m$L	1.12e9	(1e9, 1.4e9)	Identifiable	Estimated
$s_{V_{IFN_{prod}}}$	Half-saturation of virions in IFN_g production, $virions/m$L	7.9e6	-	Non-identifiable	Estimated
t_{IDC}	Time delay in IDC maturation and migration to the lymph nodes, day	0.63	(0.52, 0.1)	Identifiable	Estimated
B_0	Initial number of B_n cells, $cells/m$L	6e4	(5.6e4, 6.3e4)	-	Calculated [35,36]
$CD4_0$	Initial number of H_n, $cells/m$L	1e5	(9.4e4, 1.06e5)	-	Calculated [35,37,38]
$CD8_0$	Initial number of T_n, $cells/mL$	3.3e4	(3.1e4, 3.5e4)	-	Calculated [35,37,38]

References

1. McDonagh M, Bell EB. The survival and turnover of mature and immature CD8 T cells. Immunology. 1995 Apr;84(4):514–20.

2. Ogg GS, Jin X, Bonhoeffer S, Moss P, Nowak MA, Monard S, et al. Decay Kinetics of Human Immunodeficiency Virus-Specific Effector Cytotoxic T Lymphocytes after Combination Antiretroviral Therapy. J Virol. 1999 Jan;73(1):797–800.

3. Condon TV, Sawyer RT, Fenton MJ, Riches DWH. Lung dendritic cells at the innate-adaptive immune interface. Journal of Leukocyte Biology. 2011 Aug 1;90(5):883–95.

4. Dalod M, Chelbi R, Malissen B, Lawrence T. Dendritic cell maturation: functional specialization through signaling specificity and transcriptional programming. The EMBO Journal. 2014 May 16;33(10):1104–16.

5. Mildner A, Jung S. Development and Function of Dendritic Cell Subsets. Immunity. 2014 May;40(5):642–56.

6. Guang Y, Hui L. Determining half-life of SARS-CoV-2 antigen in respiratory secretion. Environ Sci Pollut Res. 2023 May 2;30(26):69697–702.

7. Lee HY, Topham DJ, Park SY, Hollenbaugh J, Treanor J, Mosmann TR, et al. Simulation and Prediction of the Adaptive Immune Response to Influenza A Virus Infection. J Virol. 2009 Jul 15;83(14):7151–65.

8. Sender R, Bar-On YM, Gleizer S, Bernshtein B, Flamholz A, Phillips R, et al. The total number and mass of SARS-CoV-2 virions. Proc Natl Acad Sci USA. 2021 Jun 22;118(25):e2024815118.

9. Chen HY, Di Mascio M, Perelson AS, Ho DD, Zhang L. Determination of virus burst size *in vivo* using a single-cycle SIV in rhesus macaques. Proc Natl Acad Sci USA. 2007 Nov 27;104(48):19079–84.

10. Czuppon P, Débarre F, Gonçalves A, Tenaillon O, Perelson AS, Guedj J, et al. Predicted success of prophylactic antiviral therapy to block or delay SARS-CoV-2 infection depends on the drug's mechanism of action [Internet]. 2020 [cited 2024 Jul 21]. Available from: http://medrxiv.org/lookup/doi/10.1101/2020.05.07.20092965

11. Einav T, Gentles LE, Bloom JD. SnapShot: Influenza by the Numbers. Cell. 2020 Jul;182(2):532-532.e1.

12. Sanjuán R. Collective properties of viral infectivity. Current Opinion in Virology. 2018 Dec;33:1–6.

13. Knight DA, Holgate ST. The airway epithelium: Structural and functional properties in health and disease. Respirology. 2003 Dec;8(4):432–46.

14. Mogensen C, Tos M. Quantitative histology of the maxillary sinus. Rhinology. 1977 Sep;15(3):129–40.

15. Chen M, Shen W, Rowan NR, Kulaga H, Hillel A, Ramanathan M, et al. Elevated ACE-2 expression in the olfactory neuroepithelium: implications for anosmia and upper respiratory SARS-CoV-2 entry and replication. Eur Respir J. 2020 Sep;56(3):2001948.

16. Venet F, Huang X, Chung CS, Chen Y, Ayala A. Plasmacytoid Dendritic Cells Control Lung Inflammation and Monocyte Recruitment in Indirect Acute Lung Injury in Mice. The American Journal of Pathology. 2010 Feb;176(2):764–73.

17. Villadangos JA, Young L. Antigen-Presentation Properties of Plasmacytoid Dendritic Cells. Immunity. 2008 Sep;29(3):352–61.

18. Kaiser ML, Rubinstein M, Vokes DE, Ridgway JM, Guo S, Gu M, et al. Laryngeal epithelial thickness: a comparison between optical coherence tomography and histology. Clinical Otolaryngology. 2009 Oct;34(5):460–6.

19. Smith TD, Corbin HM, King SEE, Bhatnagar KP, DeLeon VB. A comparison of diceCT and histology for determination of nasal epithelial type. PeerJ. 2021 Nov 3;9:e12261.

20. Allman D, Northrup DL. B-Cell Development*. In: Comprehensive Toxicology [Internet]. Elsevier; 2010 [cited 2024 Jul 22]. p. 35–52. Available from: https://linkinghub.elsevier.com/retrieve/pii/B9780080468846006035

21. Kaur A, Di Mascio M, Barabasz A, Rosenzweig M, McClure HM, Perelson AS, et al. Dynamics of T- and B-Lymphocyte Turnover in a Natural Host of Simian Immunodeficiency Virus. J Virol. 2008 Feb;82(3):1084–93.

22. Mankarious S, Lee M, Fischer S, Pyun KH, Ochs HD, Oxelius VA, et al. The half-lives of IgG subclasses and specific antibodies in patients with primary immunodeficiency who are receiving intravenously administered immunoglobulin. J Lab Clin Med. 1988 Nov;112(5):634–40.

23. Auner HW, Beham-Schmid C, Dillon N, Sabbattini P. The life span of short-lived plasma cells is partly determined by a block on activation of apoptotic caspases acting in combination with endoplasmic reticulum stress. Blood. 2010 Nov 4;116(18):3445–55.

24. Slifka M, Ahmed R. Long-term humoral immunity against viruses: revisiting the issue of plasma cell longevity. Trends in Microbiology. 1996 Oct;4(10):394–400.

25. Hu B, Guo H, Zhou P, Shi ZL. Characteristics of SARS-CoV-2 and COVID-19. Nat Rev Microbiol. 2021 Mar;19(3):141–54.

26. Zhang B, Yue D, Wang Y, Wang F, Wu S, Hou H. The dynamics of immune response in COVID-19 patients with different illness severity. Journal of Medical Virology. 2021 Feb;93(2):1070–7.

27. Corti D, Lanzavecchia A. Efficient Methods To Isolate Human Monoclonal Antibodies from Memory B Cells and Plasma Cells. Crowe Jr. JE, Boraschi D, Rappuoli R, editors. Microbiol Spectr. 2014 Sep 19;2(5):2.5.24.

28. Corti D, Voss J, Gamblin SJ, Codoni G, Macagno A, Jarrossay D, et al. A Neutralizing Antibody Selected from Plasma Cells That Binds to Group 1 and Group 2 Influenza A Hemagglutinins. Science. 2011 Aug 12;333(6044):850–6.

29. Eyer K, Doineau RCL, Castrillon CE, Briseño-Roa L, Menrath V, Mottet G, et al. Single-cell deep phenotyping of IgG-secreting cells for high-resolution immune monitoring. Nat Biotechnol. 2017 Oct;35(10):977–82.

30. Hibi T, Dosch H. Limiting dilution analysis of the B cell compartment in human bone marrow. Eur J Immunol. 1986 Jan;16(2):139–45.

31. Kometani K, Nakagawa R, Shinnakasu R, Kaji T, Rybouchkin A, Moriyama S, et al. Repression of the Transcription Factor Bach2 Contributes to Predisposition of IgG1 Memory B Cells toward Plasma Cell Differentiation. Immunity. 2013 Jul;39(1):136–47.

32. Lanzavecchia A. Dissecting human antibody responses: useful, basic and surprising findings. EMBO Mol Med. 2018 Mar;10(3):e8879.

33. Justiz Vaillant AA, Jamal Z, Patel P, Ramphul K. Immunoglobulin. In: StatPearls [Internet]. Treasure Island (FL): StatPearls Publishing; 2023 [cited 2023 Dec 4]. Available from: http://www.ncbi.nlm.nih.gov/books/NBK513460/

34. Ridley RG. Antibodies: A Laboratory Manual. Edited by Ed Harlow and David Lane. Cold Spring Harbor: Cold Spring Harbor Laboratory. New York. 1988. 726 pages. Paper $50.00. ISBN 0 87969 314 2. Genet Res. 1989 Oct;54(2):161–161.

35. Hatton IA, Galbraith ED, Merleau NSC, Miettinen TP, Smith BM, Shander JA. The human cell count and size distribution. Proc Natl Acad Sci USA. 2023 Sep 26;120(39):e2303077120.

36. Mahendra A, Haque A, Prabakaran P, Mackness BC, Fuller TP, Liu X, et al. Honing-in antigen-specific cells during antibody discovery: a user-friendly process to mine a deeper repertoire. Commun Biol. 2022 Oct 30;5(1):1157.

37. Janeway C, editor. Immunobiology: the immune system in health and disease ; [animated CD-ROM inside]. 5. ed. New York, NY: Garland Publ. [u.a.]; 2001. 732 p.

38. Punt J. Adaptive Immunity. In: Cancer Immunotherapy [Internet]. Elsevier; 2013 [cited 2024 Jul 21]. p. 41–53. Available from: https://linkinghub.elsevier.com/retrieve/pii/B978012394296800004X

39. Marino S, Kirschner DE. The human immune response to Mycobacterium tuberculosis in lung and lymph node. Journal of Theoretical Biology. 2004 Apr;227(4):463–86.

40. Zhou Z, Li D, Zhao Z, Shi S, Wu J, Li J, et al. Dynamical modelling of viral infection and cooperative immune protection in COVID-19 patients. Jenner AL, editor. PLoS Comput Biol. 2023 Sep 1;19(9):e1011383.

41. Zimmermann A, Hänsel R, Gemünden K, Kegel-Hübner V, Babel J, Bläker H, et al. In Vivo and In Vitro Characterization of Primary Human Liver Macrophages and Their Inflammatory State. Biomedicines. 2021 Apr 9;9(4):406.

42. Ginhoux F, Guilliams M. Tissue-Resident Macrophage Ontogeny and Homeostasis. Immunity. 2016 Mar;44(3):439–49.

43. Heider S, Metzner C. Quantitative real-time single particle analysis of virions. Virology. 2014 Aug;462–463:199–206.

44. Yao Y, Wang H, Liu Z. Expression of ACE2 in airways: Implication for COVID-19 risk and disease management in patients with chronic inflammatory respiratory diseases. Clin Experimental Allergy. 2020 Dec;50(12):1313–24.

45. Hönzke K, Obermayer B, Mache C, Fatykhova D, Kessler M, Dökel S, et al. Human lungs show limited permissiveness for SARS-CoV-2 due to scarce ACE2 levels but virus-induced expansion of inflammatory macrophages. Eur Respir J. 2022 Dec;60(6):2102725.

46. Lamers MM, Haagmans BL. SARS-CoV-2 pathogenesis. Nat Rev Microbiol. 2022 May;20(5):270–84.

47. Bakhshandeh B, Sorboni SG, Javanmard AR, Mottaghi SS, Mehrabi M reza, Sorouri F, et al. Variants in ACE2; potential influences on virus infection and COVID-19 severity. Infection, Genetics and Evolution. 2021 Jun;90:104773.

48. Jia HP, Look DC, Shi L, Hickey M, Pewe L, Netland J, et al. ACE2 Receptor Expression and Severe Acute Respiratory Syndrome Coronavirus Infection Depend on Differentiation of Human Airway Epithelia. J Virol. 2005 Dec 15;79(23):14614–21.

49. Ahlfors EE, Laksson PÅ, Bergstresser PR. Langerhans cell surface densities in rat oral mucosa and human buccal mucosa. J Oral Pathology Medicine. 1985 May;14(5):390–7.

50. Jahnsen FL, Gran E, Haye R, Brandtzaeg P. Human Nasal Mucosa Contains Antigen-Presenting Cells of Strikingly Different Functional Phenotypes. Am J Respir Cell Mol Biol. 2004 Jan;30(1):31–7.

51. Schon-Hegrad MA, Oliver J, McMenamin PG, Holt PG. Studies on the density, distribution, and surface phenotype of intraepithelial class II major histocompatibility complex antigen (Ia)-bearing dendritic cells (DC) in the conducting airways. The Journal of experimental medicine. 1991 Jun 1;173(6):1345–56.

52. Prestin S, Rothschild SI, Betz CS, Kraft M. Measurement of epithelial thickness within the oral cavity using optical coherence tomography. Head & Neck. 2012 Dec;34(12):1777–81.

53. Thurlbeck WM. The internal surface area of nonemphysematous lungs. Am Rev Respir Dis. 1967 May;95(5):765–73.

54. Hogan J, Smith P, Heath Peter Harris D. The thickness of the alveolar capillary wall in the human lung at high and low altitude. British Journal of Diseases of the Chest. 1986 Jan;80:13–8.

55. Bowden D. H. BF. Measurement of the thickness of the bronchial epithelium. 1989;22(16):75.

56. Saetta M, Di Stefano A, Maestrelli P, Ferraresso A, Drigo R, Potena A, et al. Activated T-Lymphocytes and Macrophages in Bronchial Mucosa of Subjects with Chronic Bronchitis. Am Rev Respir Dis. 1993 Feb;147(2):301–6.

57. Weber M, Wehrhan F, Baran C, Agaimy A, Büttner-Herold M, Öztürk H, et al. Malignant transformation of oral leukoplakia is associated with macrophage polarization. J Transl Med. 2020 Dec;18(1):11.

58. Miyakawa N, Nishikawa M, Takahashi Y, Ando M, Misaka M, Watanabe Y, et al. Prolonged Circulation Half-life of Interferon γ Activity by Gene Delivery of Interferon γ–Serum Albumin Fusion Protein in Mice. Journal of Pharmaceutical Sciences. 2011 Jun;100(6):2350–7.

59. Bajetta E, Del Vecchio M, Mortarini R, Nadeau R, Rakhit A, Rimassa L, et al. Pilot study of subcutaneous recombinant human interleukin 12 in metastatic melanoma. Clin Cancer Res. 1998 Jan;4(1):75–85.

60. Pol JG, Caudana P, Paillet J, Piaggio E, Kroemer G. Effects of interleukin-2 in immunostimulation and immunosuppression. Journal of Experimental Medicine. 2020 Jan 6;217(1):e20191247.

61. De Sousa-Pereira P, Woof JM. IgA: Structure, Function, and Developability. Antibodies. 2019 Dec 5;8(4):57.

62. Keyt BA, Baliga R, Sinclair AM, Carroll SF, Peterson MS. Structure, Function, and Therapeutic Use of IgM Antibodies. Antibodies. 2020 Oct 13;9(4):53.
