# Supplementary material for: A Modular Mathematical Model of the Immune Response for Investigating the Pathogenesis of Infectious Diseases
Source: Viruses. 2025 Apr 22;17(5):589. doi: 10.3390/v17050589 (PMC12115727; doi:10.3390/v17050589)
Supplement: Supplementary file 1 [file viruses-17-00589-s001.zip › viruses-3549703-supplementary/Supplementary Table S2. Initial Values-tracked.pdf]

**Table S2. Initial Values**

| Upper Airways | | |
|---|---|---|
| **Notation** | **Variable, Units** | **Value** |
| $EP$ | Healthy epithelial cells, cells | $5.5 \times 10^6$ |
| $IDC$ | Immature dendritic cells, cells/mL | $2.5 \times 10^6$ |
| $V$ | Initial viral load, virions/mL | $1000$ |

| Upper Airways (Lymph Nodes) | | |
|---|---|---|
| **Notation** | **Variable, Units** | **Value** |
| $B_n$ | Naive B cells, cells/mL | $3.3 \times 10^4$ |
| $T_n$ | Naive T cells, cells/mL | $1.6 \times 10^4$ |

| Lungs | | |
|---|---|---|
| **Notation** | **Variable, Units** | **Value** |
| $EP$ | Healthy epithelial cells, cells | $5.5 \times 10^8$ |
| $IDC$ | Immature dendritic cells, cells/mL | $1.2 \times 10^6$ |
| $M_r$ | Resting macrophages, cells/mL | $4.0 \times 10^5$ |

| Lungs (Lymph Nodes) | | |
|---|---|---|
| **Notation** | **Variable, Units** | **Value** |
| $B_n$ | Naive B cells, cells/mL | $6.0 \times 10^4$ |
| $H_n$ | Naive CD4+ T cells, cells/mL | $1.0 \times 10^5$ |
| $T_n$ | Naive CD8+ T cells, cells/mL | $3.3 \times 10^4$ |
